# FLI1 induces erythroleukemia through opposing effects on UBASH3A and UBASH3B expression

**DOI:** 10.1186/s12885-024-12075-2

**Published:** 2024-03-09

**Authors:** Jie Wang, Chunlin Wang, Anling Hu, Kunlin Yu, Yi Kuang, Babu Gajendran, Eldad Zacksenhaus, Klarke Michael Sample, Xiao Xiao, Wuling Liu, Yaacov Ben-David

**Affiliations:** 1 State Key Laboratory for Functions and Applications of Medicinal Plants, Guizhou Medical University, Guiyang-550014, Guizhou, People’s Republic of China; 2 Natural Products Research Center of Guizhou Province, High Tech Zone, Province Science City, Baiyun District, Guiyang, 550014 China; 3 School of Pharmaceutical Sciences, Guizhou Medical University, Guizhou Province, Guiyang, 550025 People’s Republic of China; 4 Department of Medicine, University of Toronto, Toronto, ON Canada; 5 Division of Advanced Diagnostics, Toronto General Research Institute, University Health Network, Toronto, ON Canada; 6 eBond Pharmaceutical Technology Ltd, Chengdu, Sichuan People’s Republic of China

**Keywords:** FLI1, Transcriptional regulation, UBASH3A, UBASH3B, HSPA1B, AP1, SYK, Leukemia proliferation, Oncogene, Tumor suppressor

## Abstract

**Background:**

FLI1 is an oncogenic transcription factor that promotes diverse malignancies through mechanisms that are not fully understood. Herein, FLI1 is shown to regulate the expression of Ubiquitin Associated and SH3 Domain Containing A/B (*UBASH3A/B*) genes. UBASH3B and UBASH3A are found to act as an oncogene and tumor suppressor, respectively, and their combined effect determines erythroleukemia progression downstream of FLI1.

**Methods:**

Promoter analysis combined with luciferase assays and chromatin immunoprecipitation (ChIP) analysis were applied on the *UBASH3A/B* promoters. RNAseq analysis combined with bioinformatic was used to determine the effect of knocking-down UBASH3A and UBASH3B in leukemic cells. Downstream targets of UBASH3A/B were inhibited in leukemic cells either via lentivirus-shRNAs or small molecule inhibitors. Western blotting and RT-qPCR were used to determine transcription levels, MTT assays to assess proliferation rate, and flow cytometry to examine apoptotic index.

**Results:**

Knockdown of FLI1 in erythroleukemic cells identified the* UBASH3A/B* genes as potential downstream targets. Herein, we show that FLI1 directly binds to the *UBASH3B* promoter, leading to its activation and leukemic cell proliferation. In contrast, FLI1 indirectly inhibits *UBASH3A* transcription via GATA2, thereby antagonizing leukemic growth. These results suggest oncogenic and tumor suppressor roles for UBASH3B and UBASH3A in erythroleukemia, respectively. Mechanistically, we show that *UBASH3B* indirectly inhibits *AP1* (*FOS* and *JUN*) expression, and that its loss leads to inhibition of apoptosis and acceleration of proliferation. UBASH3B also positively regulates the *SYK* gene expression and its inhibition suppresses leukemia progression. High expression of *UBASH3B* in diverse tumors was associated with worse prognosis. In contrast, *UBASH3A* knockdown in erythroleukemic cells increased proliferation; and this was associated with a dramatic induction of the *HSP70* gene, *HSPA1B*. Accordingly, knockdown of *HSPA1B* in erythroleukemia cells significantly accelerated leukemic cell proliferation. Accordingly, overexpression of *UBASH3A* in different cancers was predominantly associated with good prognosis. These results suggest for the first time that *UBASH3A* plays a tumor suppressor role in part through activation of HSPA1B.

**Conclusions:**

FLI1 promotes erythroleukemia progression in part by modulating expression of the oncogenic *UBASH3B* and tumor suppressor *UBASH3A*.

**Supplementary Information:**

The online version contains supplementary material available at 10.1186/s12885-024-12075-2.

## Introduction

UBASH3A (STS-2/TULA/ CLIP4) and UBASH3B (STS-1/TULA-2) belong to the ubiquitin-associated and Src-homology 3 domain-containing (UBASH3) family [1]. While *UBASH3B* is ubiquitously expressed [2], *UBASH3A* expression is restricted to lymphoid tissues [3]. *UBASH3A* is a negative regulator of T-cell activation and function through regulation of ZAP70 and TCR activation [2, 4]. Genetic variants in this gene are associated with several distinct autoimmune diseases [5–8], including type 1 diabetes, and rheumatoid anthesis, although the underlying mechanism is still unknown. UBASH3A has three structural domains: 1) N-terminal UBA (ubiquitin-associated), 2) SH3 (Src homology 3), and 3) PGM (phosphoglycerate mutase-like/C-terminal histidine phosphatase) domain [8]. The UBA domain can bind to mono-ubiquitin and lysine-63 and methionine-1-linked polyubiquitin chains. UBASH3A has four known ubiquitination sites at lysine residues 15, 202, 309, and 358. Monoubiquitination at Lys 202 causes UBASH3A to adopt a closed conformation, which prevents the binding of the UBA domain to substrates [9]. The SH3 domain interacts with dynamin [10] (which is required for endocytosis) and with CBL [11] (an E3 ubiquitin ligase). The PGM domain mediates self-dimerization [12], which exhibits very weak, possibly acid-dependent, phosphatase activity (despite its structural similarity to the more active PGM domain in UBASH3B) [3, 13].

UBASH3B has similar structural domains to UBASH3A and some overlapping functions. However, UBASH3B suppresses T-cell receptor (TCR) signaling by dephosphorylating ZAP-70 and Syk, two key molecules involved in the amplification of TCR-triggered signals [3, 14, 15]. UBASH3A and UBASH3B knockout mice exhibit no obvious phenotype until the T cell receptor (TCR) is stimulated. Upon stimulation, T cells from UBASH3A / UBASH3B double deficient mice are hyper-proliferative and produce more IL-2 and IFNγ than wild-type T cells [3], underscoring the vital role UBASH3A/B in T cell regulation and autoimmunity. UBASH3B expression is implicated in various cancers through its ability to bind CBL and block its ubiquitination activity [16, 17].

We have previously shown that Protein Kinase C Delta (PKCẟ) downregulation in TPA (4b-12-O-tetradecanoylphobol-13-acetate)-resistant cell lines, can inhibit erythroleukemia when paired with *UBASH3B* knockdown [18]. Interestingly, TPA and several PKCẟ agonists have previously been reported to activate the transcription factor FLI1 through increased protein phosphorylation [19–21]. We hypothesized that this could be due to FLI1 regulation of UBASH3B. The ETS transcription factor Fli-1 was first identified as a target of retroviral insertional activation in erythroleukemia induced by the Friend virus [22, 23]. Human FLI1 oncogene was later implicated in various types of cancers through translocations or overexpression [24–37]. Indeed, Fli-1 transcriptional activation affects several hallmarks of cancer, including proliferation, survival, differentiation, angiogenesis, genomic instability, and immune surveillance [37]. In this study, we show that leukemias expressing high FLI1 produce either higher UBASH3B or lower UBASH3A. We also show that FLI1 controls the expression of these ubiquitin associated ligase genes in erythroleukemic cells. These results suggest that FLI1 promotes erythroleukemia and possibly progression of other cancers in part by balancing oncogenic effect of UBASH3B and tumor suppressor activity of UBASH3A.

## Materials and methods

### Cells, culture conditions and drug therapy

The human leukemia (HEL 92.1.7, K562) and epithelial-like HEK293T (CRL-3216) cell lines were obtained from ATCC (US) and tested negative for mycoplasma. These cell lines were cultured and maintained in Dulbecco’s Modified Eagle Medium supplemented with HyClone 5% fetal bovine serum (GE Healthcare, US).

For drug treatment, cells were treated with Camptothecin (MedChemExpress, CN), T5224 (APExBIO, CN) and R406 (Beyotime Biotechnology, CN) for indicated times and used for cell proliferation analysis. Generation of K562-fli1 cells was previously described [21]. For FLI1 induction, cells were treated with 5μM of doxycycline (Solarbio, CN).

### RNA preparation and RT-qPCR

Total RNA was extracted using Trizol reagent (Thermo Fisher Scientific, US), cDNA was synthesis using the PrimeScript RT Reagent Kit (Takara Bio, CN) and RT-qPCR analysis using the FastStart Universal SYBR Green Master Mix (Roche, CH) on a Step One Plus Real-time PCR system (Applied Biosystems/Thermo Fisher Scientific, US). The expression of the test genes was given as relative to *β Actin*. Three biological replicates in triplicate (*n* = 3) were performed for each gene. The primers sequences were listed in the Table 1.

**Table 1:** Primers sequences used for RT-qPCR

**Gene**		**Sequence(5’-3’)**
UBASH3A	sense	GGTGCAAATCGTCAACACCT
	antisense	GCAAAATCCCCACATTCCCG
UBASH3B	sense	ACCATCAAGCATGGATCGGC
	antisense	CCGACATGGGAGAATAACCAGT
FLI1	sense	CAGCCCCACAAGATCAACCC
	antisense	CACCGGAGACTCCCTGGAT
HSPA1B	sense	TTTGAGGGCATCGACTTCTACA
	antisense	CCAGGACCAGGTCGTGAATC
FOS	sense	CCGGGGATAGCCTCTCTTACT
	antisense	CCAGGTCCGTGCAGAAGTC
JUN	sense	TCCAAGTGCCGAAAAAGGAAG
	antisense	CGAGTTCTGAGCTTTCAAGGT
SYK	sense	TGCACTATCGCATCGACAAAG
	antisense	CATTTCCCTGTGTGCCGATTT
GAPDH	sense	GCCAGTAGAGGCAGGGATGATGTTC
	antisense	CCATGTTCGTCATGGGTGTGAACCA

### Promoter analysis and luciferase assays

The *UBASH3A* and *UBASH3B* promoter regions (see Figs. 2A and 3A) were amplified by PCR, cloned into the luciferase reporter vector pGL3-basic (Promega, US), and used in a luciferase activity assay, as previously described [38]. Briefly, 2.5 μg of the indicated promoter was co-transfected with either MigR1 (2.5 μg) or MigR1-FLI1 (2.5 μg) using a Lipofectamine 2000 kit (Thermo Fisher Scientific) into epithelial HEK-293T cells which seeded onto 6-well plates one day before, according to the manufacturer’s protocol. Renilla luciferase (Promega, US) was used as an internal control for transfection efficiency.

### ShRNA and siRNA expression

The construction of sh*FLI1* cells has been previously described [38]. sh*UBASH3A*, sh*UBASH3B*, and sh*HSPA1B* and scrambled control vectors were generated by inserting the corresponding shRNA sequence containing oligonucleotides and scrambled DNAs into the restriction enzyme sites BcuI within the PLent-GFP expression vector (obtained from Vigene Bioscience, US). The lentivirus particles were generated by co-transfecting shRNA PLent-GFPs (10 µg) with packaging plasmids psPAX2 (5 µg) and pMD2G (10 µg) (Addgene plasmid #12,259 & #12,260) into HEK293T cells, using lipofectamine 2000. The supernatants were collected two days after transfection to transduce HEL cells. The positive cells were then selected via incubation with a medium containing puromycin (5 µg/ml; Solarbio, CN). ShRNA sequences are listed in Table 2.

**Table 2:** ShRNA sequences

**shRNA**	**Sequence (5’-3’)**
shUBASH3A1	GCTGCATGATCATTGCAATTTCAAGAGAATTGCAATGATCATGCAGCTTTTTT
shUBASH3A2	GGGATCAAAGACTTTGAAATTCAAGAGATTTCAAAGTCTTTGATCCCTTTTTT
shUBASSH3A3	CGAGTGGAACCTGGAATCTTTCAAGAAAAGATTCCAGGTTCCACTCGTTTTTT
shHSPA1B1	GCTGACCAAGATGAAGGAGATTTCAAGAGAATCTCCTTCATCTTGGTCAGCTTTTTT
shHSPA1B2	GCGCAACGTGCTCATCTTTGTTCAAGAGACAAAGATGAGCACGTTGCGCTTTTTT
shHSPA1B3	GGGCCATGACGAAAGACAATTCAAGAGATTGTCTTTCGTCATGGCCCTTTTTT
shUBASH3B1	GCGGCAGTATGAAGATCAAGGTTCAAGAGACCTTGATCTTCATACTGCCGCTTTTTT
shUBASH3B2	GGTGAAGCCTTGTTAGAAAGTTTCAAGAGAACTTTCTAACAAGGCTTCACCTTTTTT
shUBASH3B3	GCGTTCAGACTGCACATAATATTCAAGAGATATTATGTGCAGTCTGAACGCTTTTTT
shUBASH3B4	GGATACCTCCATCAGAGTTAGTTCAAGAGACTAACTCTGATGGAGGTATCCTTTTTT
Scrambled	TTCTCCGAACGTGTCACGTTTCAAGAGAACGTGACACGTTCGGAGAATTTTTT

*UBASH3A* siRNAs and negative control were purchased from GenePharma (CN). The *UBASH3A* siRNA was transfected into sh*UBASH3B* cells using Lipofectamine 2000. Two days after transfection, cells were collected, RNA was extracted, and RT-qPCR was used to detect *UBASH3A*. For proliferation analysis, cells were transfected with siRNA for 48 h and assessed using an MTT assay every day for three days. SiRNA sequences listed in Table 3.

**Table 3:** SiRNA sequences

**siRNA**		**Sequence(5’-3’)**
Negative control	sense	UUCUCCGAACGUGUCACGUTT’
	antisense	ACGUGACACGUUCGGAGAATT’
GAPDH Positive control	sense	UGACCUCAACUACAUGGUUTT
	antisense	AACCAUGUAGUUGAGGUCATT
siUBASH3A1	sense	GCUGCAUGAUCAUUGCAAUTT
	antisense	AUUGCAAUGAUCAUGCAGCTT
siUBASH3A2	sense	GAGCCCUAUUCCAGUACAATT
	antisense	UUGUACUGGAAUAGGGCUCTT
siUBASH3A3	sense	CCACUCCUGAUGGGAAAUATT
	antisense	UAUUUCCCAUCAGGAGUGGTT
siUBASH3A4	sense	CGGGUGUCAUCCUAAUUGUTT
	antisense	ACAAUUAGGAUGACACCCGTT

### RNAseq analysis and bioinformatics

Total RNA samples isolated from designated cells and appropriate controls were sent for RNAseq at BGI genomics (CN). BGI also performed the data preprocessing, which we used to analyze the gene expression profiles between shRNA-mediate knocked downed genes and the scrambled control group. Differentially expressed genes (DEGs) were determined by the condition (log2FoldChange ≤ -1 or ≥ 1, padj < 0.05) and then analyzed by Kyoto Encyclopedia of Genes and Genomes (KEGG) pathway enrichment. Differentially expressed genes (DEGs) for UBASH3B and UBASH3A were shown in Supplementary Tables 1 and 2, respectively. The TCGA data analysis was obtained using GEPIA2 resources (http://gepia2.cancer-pku.cn/).

### Western blotting

Total protein from cell lines was extracted using RIPA buffer (Beyotime Institute of Biotechnology, CN) containing 1:100 PMSF (Solarbio, CN). The protein concentration was determined using a BCA kit (Solarbio, CN) according to the manufacturer's protocol. Load equal amounts of protein into the wells of the SDS-PAGE gel and transferred to PVDF membrane. The membrane was blocked using non-fat milk for 1 h at room temperature. Incubate the membrane with primary antibody in blocking buffer overnight at 4°C. After washed by TBST (Beyotime Institute of Biotechnology, CN) at room temperature for three times, the membrane was incubated with Anti-rabbit IgG (H + L) DyLight™ 800 4X PEG Conjugated secondary antibody (5151s, Cell Signaling Technology, US) in blocking buffer at room temperature for 1 h. The following primary antibodies were used: anti-FLI1 (ab133485, Abcam, UK), anti-UBASH3A (15,823–1-AP, Proteintech, DE), anti-UBASH3B (19,563–1-AP, Proteintech, DE), polyclonal rabbit test primary antibodies; anti-GAPDH (G9545, Sigma Aldrich, US). Antibody dilution was conducted according to the manufacturer’s instructions. The Odyssey system (LICOR Biosciences) was used for western blot membrane imaging and analysis.

### Apoptosis

Cells were incubated with compounds or vehicle for 24 h, as previously described [29]. Treated cells were washed by PBS, stained by Annexin V and PI apoptosis detection kit (BD Biosciences, US), following the kit guidelines and analyzed by flow cytometer.

### Chromatin immunoprecipitation (ChIp) analysis

The ChIp analysis was performed, as previously published [29]. In brief, formaldehyde was used to crosslink erythroleukemia HEL cells before they were centrifuged and the pellet was then resuspended in Magna ChIp A/G kit lysis solution (Sigma-Aldrich, US). The fixed pellet was sonicated using a Sonics Vibra VCX150 (Ningbo Scientz Biotechnology, CN). A small aliquot of the chromatin was taken out to serve as an input control. Protein G Sepharose beads (Cell Signaling Technology, US) were added to the chromatin and incubated for one hour at room temperature. The immunoprecipitations were performed overnight at 4°C with 1 μg of ChIp grade anti-FLI1 antibody (ab15289, Abcam, UK) and the negative control rabbit immunoglobulin G (IgG) antibody (Cell Signaling Technology, US). After centrifugations, the chromatin precipitates were washed and reverse crosslinked. The precipitated chromatins were then incubated with proteinase K at 56°C for two hours, DNA purified with one phenol chloroform extraction and resuspended in TE buffer. RT-qPCR was performed using this DNA to determine the amount of FLI1 binding within the promoter region. The percentage of input was calculated as previously described [29]. Amplified DNAs were also resolved on a 2% agarose gel. The ChIp was performed at least in three independent experiments. The primer sequences for the ChIp PCRs are as follows. Forward: GTCCTGGAAGAGCATTCTGCA; Reverse: AGCAGGGAGATAAGACAGCT.

### Statistical analysis

The statistical analysis was performed using a two-tailed Student t-test or a one-way ANOVA with Tukey’s post hoc test, using Prism 9 software (GraphPad Software Inc, US). The P values were indicated within the figures using a standard scheme, *P* < 0.05 (*), *P* < 0.01 (**), *P* < 0.001 (***), and *P* < 0.0001 (****). Where appropriate, the data were displayed using the mean (± SEM) from at least three independent experiments.

## Results

### FLI1 regulates UBASH3B positively and UBASH3A negatively in leukemic cells

**Fig. 1:**
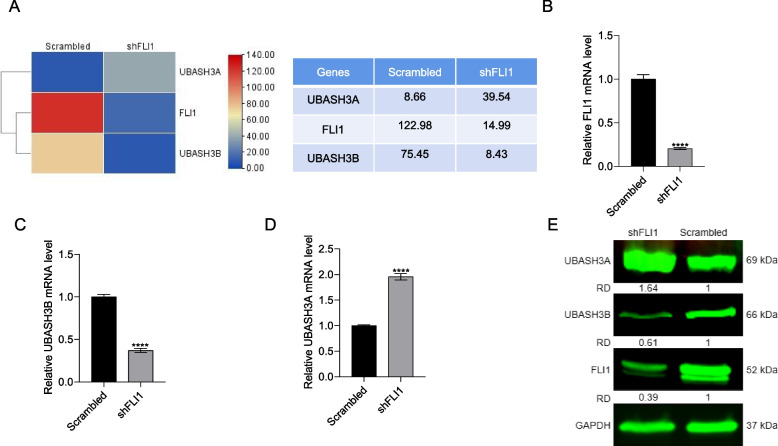
FLI1 regulates *UBASH3A* and *UBASH3B* transcription in leukemic cells. **A** Heatmap of *UBASH3A* and *UBASH3B* expression following *FLI1* knockdown (sh*FLI1*) versus control leukemia cells. **B**-**D** RT-qPCR analysis for expression of *FLI1* (**B**), *UBASH3B* (**C**), and *UBASH3A* (**D**) in sh*FLI1* cells versus scrambled control leukemic cells. **E** Western blot analysis for FLI1, UBASH3A, and UBASH3B compared to the loading control GAPDH in sh*FLI1* versus scrambled control HEL cells. *P* < 0.001 (***). Relative density (Rd) determined by densitometer is shown. The full-length blots/gels for Fig. 1E are presented in Supplementary Fig. 9

While FLI1 is known to promote the initiation and progression of leukemias and other cancers [37], the underlying mechanism is not fully understood. To uncover its downstream targets, RNAseq analysis was used to identify genes whose expression is modulated in response to shRNA knockdown of FLI1 (sh*FLI1)* in leukemic HEL cells [38]. Knockdown of FLI1 in HEL cells was previously shown to slow down proliferation, alter the cell cycle and induce apoptosis [29, 37]. Among the affected genes, *UBASH3B* expression was strongly downregulated in sh*FLI1* versus scrambled controlled HEL cells, whereas *UBASH3A* was elevated (Fig. 1A). These results raised the possibility that the *UBASH3AB/A* variants may affect erythroleukemia progression through opposing functions. First, we confirmed these results by RT-qPCR, where reduced *FLI1* expression (Fig. 1B) in sh*FLI1* cells was indeed associated with a decreased *UBASH3B* (Fig. 1C) and increased *UBASH3A* (Fig. 1D). Western blotting further confirmed this expression pattern (Fig. 1E). In K562-fli1 cells, expression of FLI1 resulted in upregulation of UBASH3B and downregulation of UBASH3A (Supplemental Fig. 1).

**Fig. 2:**
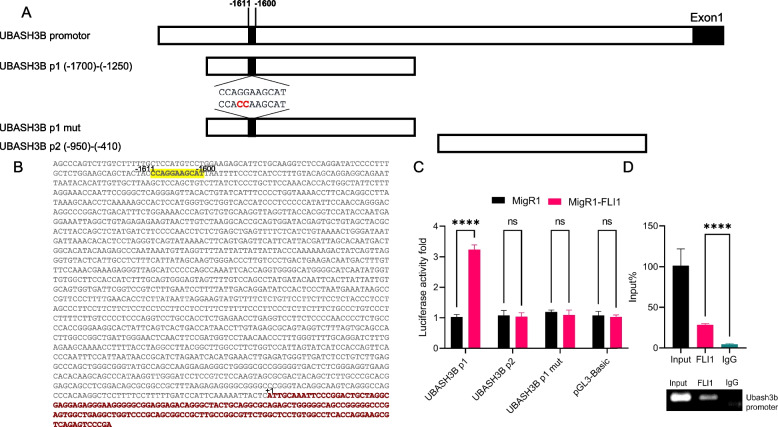
FLI1 binds to the *UBASH3B* promoter and activates its expression. **A** The genomic structure of the *UBASH3B* promoter its indicated derivatives *UBASH3B* P1, *UBASH3B* P2, and *UBASH3B* P1-mut, which were subcloned upstream from the PGL3 luciferase reporter plasmid. **B** The *UBASH3B* promoter sequence and its potential FLI1 binding site. **C** Luciferase activity in HEK293T cells transfected with the *UBASH3B* P1/P2 and *UBASH3B* P1-mut luciferase vectors transfected with either *FLI1* expression vector MigR1-*Fli1* or control plasmid MigR1. **D** Chromatin immunoprecipitation (ChIp) analysis of the human *UBASH3B* promoter in HEL erythroleukemic cells for binding to FLI1 by RT-qPCR (top panel). The lower panel shows the gel image for the immunoprecipitated PCR-amplified band relative to the input. *P* < 0.0001 (****). The full-length gel for Fig. 2D are presented in Supplementary Fig. 10

To determine whether the differential effect of FLI1 on *UBASH3A* and *UBASH3B* is mediated by direct transcriptional regulation, we performed an in vitro FLI1 promoter binding assays. Figure 2A depicts a schematic of the *UBASH3B* promoter (P1 and P2), which contains a putative FLI1 binding site at position -1611 to -1600 in P1 (Fig. 2B). The FLI1 binding site is absent in the *UBASH3B*-P2 promoter, which was used as a negative control (Fig. 2A). Transfection of these luciferase reporter plasmids into HEK293T cells alongside either the *FLI1* expression vector (MigR1-*FLI1*) or vector control (MigR1) resulted in significantly higher luciferase activity for the P1 promoter when co-transfected with MigR1-*FLI1*. In contrast, the P2 promoter was refractory to FLI1 expression. Mutation within the FLI1 binding site on the P1 promoter (*UBASH3B* P1 mut, Fig. 2A) did not affect basal gene expression but conferred resistance to FLI1 over-expression (Fig. 2C). FLI1 binding to the *UBASH3B* promoter was further confirmed by Chromatin Immunoprecipitation (ChIp) (Fig. 2D), in which significantly higher binding was observed using FLI1 antibody versus control IgG. Moreover, in ChIPseq in GEO database, FLI1 strongly binds to promoter of the *UBASH3B* gene (Supplemental Fig. 2). These results demonstrate direct regulation of the *UBASH3B* expression by FLI1.

**Fig. 3:**
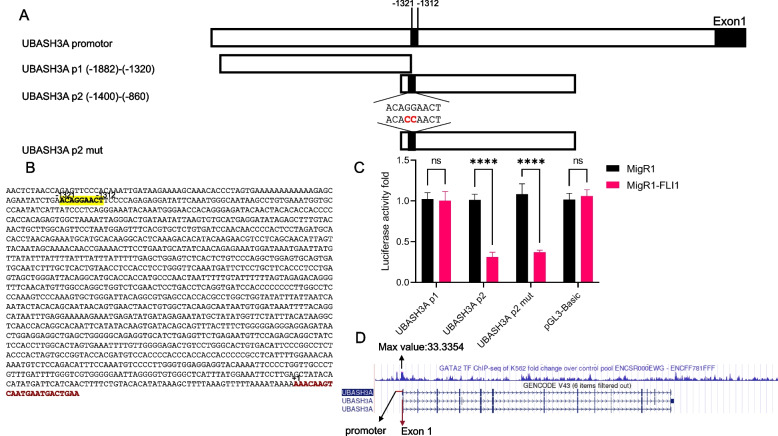
FLI1 interacts with the *UBASH3A* promoter and reduces its expression. **A** The genomic structure of the human *UBASH3A* promoter and its sub-derivatives *UBASH3A* P1 and *UBASH3A* P2 as well as their derivative mutant DNA subcloned downstream of the pGL3-basic luciferase reporter plasmid. **B** The sequence of the *UBASH3A* promoter and its potential FLI1 binding site. **C** HEK293T cells were co-transfected with the *UBASH3A* P1/P2 and mutant (UBASH3A P2-mut) luciferase vectors and either MigR1-*Fli1* or control plasmid MigR1. Luciferase activity was determined, as described in materials and methods. **D** ENCODE data showing the binding of GATA2 to the *UBASH3A* promoter region—Maximum fold change: 33.3354

A similar strategy was used to generate the plasmids containing *UBASH3A*-P1 and P2 promoters (Fig. 3A); the latter contained FLI1 binding site at positions -1321 to -1312 (Fig. 3B). Both the P1 and P2 promoters were associated with similar activation when co-transfected with the MigR1 expression vector (Fig. 3C), but MigR1-*FLI1* inhibited luciferase activity supporting the negative regulation of *UBASH3A* by FLI1. Interestingly, MigR1-*FLI1* also inhibited luciferase activity when the FLI1 binding site within the P2 promoter was mutated (*UBASH3A* P2 mut, Fig. 3A and C). In the ChIp assay, FLI1 failed to bind the putative binding site identified within the *UBASH3A* promoter (data not shown). These results suggest that FLI1 indirectly regulates *UBASH3A* expression through another site or transcription factor. Indeed, strong binding between the GATA2 transcription factor within the *UBASH3A* promoter has been identified in the ENCODE database [39] (Fig. 3D). GATA2 is regulated by FLI1[38] and thus may mediate the negative effect of FLI1 on *UBASH3A* expression in leukemic cells.

### UBASH3A and UBASH3B downregulation affects leukemia cell proliferation

**Fig. 4:**
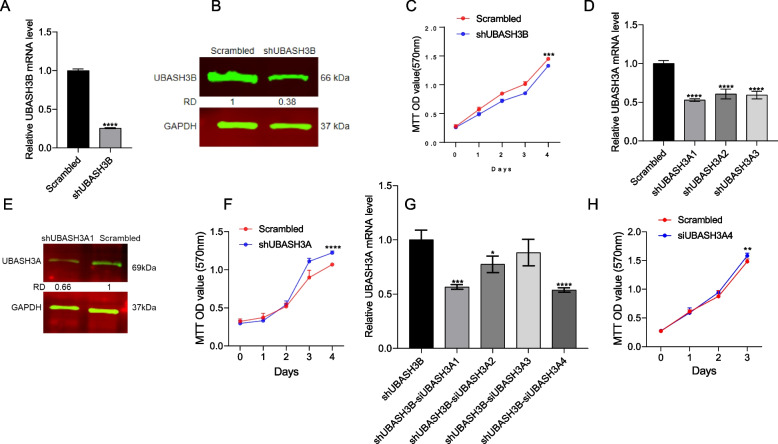
Control of cell proliferation by *UBASH3A* and *UBASH3B*. **A**, **B** The expression of *UBASH3B* by RT-qPCR (**A**) and western blot (**B**) in sh*UBASH3B* and scrambled control cells. **C** The cell proliferation rate of sh*UBASH3B* versus scrambled control cells. **D** Expression of *UBASH3A* in lentivirus transduced sh*UBASH3A*1-A3 cells by RT-qPCR. **E** UBASH3A levels in sh*UBASH3A*1 cells by western blot. **F** The cell proliferation rate for sh*UBASH3A*1 versus the scrambled control. **G** Knockdown of *UBASH3A* in sh*UBASH3B* cells via siRNA (siU*BASH3A*1-si*UBASH3A*4), as detected via RT-qPCR. **H** The proliferation of sh*UBASH3B* cells after treatment with si*UBASH3A*4. *P* < 0.05 (*), *P* < 0.01 (**), *P* < 0.001 (***), and *P* < 0.0001 (****). The full-length blots/gels for Fig. 4B and 4E are presented in Supplementary Fig. 11

As *FLI1* knockdown blocks leukemia cell proliferation [38], we next examined impact of *UBASH3A* and *UBASH3B* on cell growth. To this end, *UBASH3B* was knocked down in HEL cells using lentivirus vectors containing four shRNAs, which resulted in reduced mRNA expression (Fig. 4A) and protein levels (Fig. 4B). Reduced expression of sh*UBASH3B* resulted in significant growth suppression compared to the control scrambled cells (Fig. 4C). The expression of FLI1 was also lightly reduced (possibly due to a positive feedback) in sh*UBASH3B* cells (Supplemental Fig. 3A and B). These results suggest an oncogenic role for UBASH3B in leukemia progression.

Three lentiviruses (sh*UBASH3A*1-3) were also used to knock down *UBASH3A* in HEL cells (Fig. 4D), resulting in reduced mRNA expression (Fig. 4D) and protein levels (Fig. 4E). Unlike the effect of UBASH3B knockdown, *UBASH3A* depletion increased cell proliferation compared to scrambled control cells (Fig. 4F), suggesting an inhibitory role for this protein in erythroleukemic cells. As *UBASH3B* knockdown inhibited cell proliferation, we examined whether UBASH3A knockdown moderated this suppressive effect. Indeed, inhibition of *UBASH3A* in sh*UBASH3B* cells using siRNA (Fig. 4G) significantly reduced growth inhibition compared to the control (Fig. 4H). The expression of the *FLI1* oncogene was increased in shUBASH3A1 cells (Supplemental Fig. 3C and D). These results indicate that UBASH3A/B have opposing effects on leukemic cell proliferation.

### UBASH3A and UBASH3B regulate the expression of common and unique genes

**Fig. 5:**
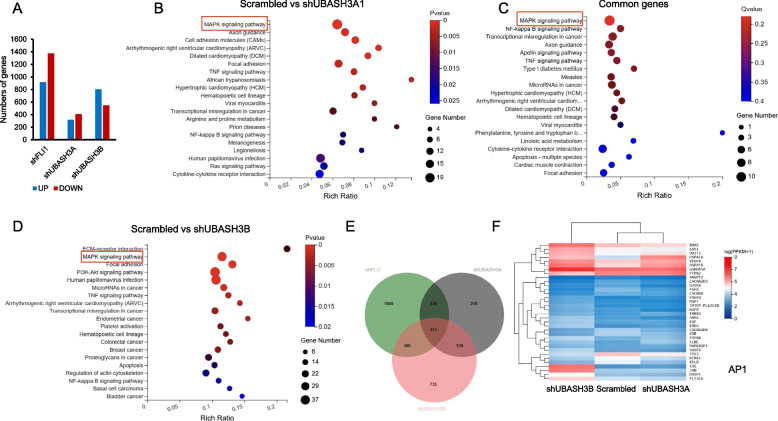
Regulation of the MAP Kinase pathway via *UBASH3A* and *UBASH3B*. **A** Compared to scrambled controls, many genes were upregulated or downregulated in shFLI1, shUBASH3A1 and shUBASH3B cells. **B**, **C** KEGG pathway enrichment analysis for sh*UBASH3A* (**B**) and sh*UBASH3B* cells (**C**). **D** KEGG pathway enrichment analysis for DEGs commonly affected by both *UBASH3A* and *UBASH3B* genes belonging to the MAP Kinase pathway. **E** Number of common or unique DEGs in shFLI1, sh*UBASH3A* and *UBASH3B* cells. **F** Heatmap showing the differentially expressed MAP Kinase genes in sh*UBASH3A*1 and sh*UBASH3B* cells

To uncover the mechanisms underlying the effect of *UBASH3A* and *UBASH3B* on leukemia progression, both sh*UBASH3B* and sh*UBASH3A* cells were assessed using RNAseq. Results from the Differentially Expressed Gene (DEG) analysis after *UBASH3B* knockdown revealed 800 genes with increased expression and 547 genes with decreased expression (Fig. 5A; Supplemental Table 2). Similarly, the DEGs in sh*UBASH3A*1 versus scrambled control cells uncovered 317 DEGs were increased and 407 decreased (Fig. 5A; Supplemental Table 1). In comparison, in shFLI1 RNAseq data [38], we identified 1373 downregulated and 916 upregulated genes (Fig. 5A). A KEGG (Kyoto Encyclopedia of Genes and Genomes) pathway enrichment analysis for both the sh*UBASH3A* (Fig. 5B) and sh*UBASH3B* (Fig. 5D) regulated genes revealed significant changes associated with the MAP Kinase pathway (Fig. 5F), indicative of overlapping gene regulation. The MAP Kinase pathway genes were also altered in the shFLI1 RNAseq data (Supplemental Fig. 4A). In this analysis, 113 DEGs were common between shFLI1, shUBASH3A and shUBASH3B cells (Fig. 5E). In addition to DEGs observed in both UBASH3A and UBASH3B cells, we identified DEGs unique to one of the two ubiquitin associated ligases (Fig. 5E). A comparison between upregulated or downregulated DEGS from UBASH3B and UBASH3A cells is shown in Supplemental Fig. 5A-D. This analysis reveals upregulation of MAP Kinase pathway genes in UBASH3B and downregulation in UBASH3A cells. The common DEGs in the MAP Kinase pathway and expression variation between the sh*UBASH3A*, sh*UBASH3B* and *shFLI1* effected genes are shown as a heatmap (Fig. 5F and Supplemental Fig. 4B). These changes may partially account for the suppressive and oncogenic differences between these UBASH3 isoforms in leukemia cells.

### UBASH3B ablation activates the AP1(FOS-JUN) pathway and blocks expression of SYK to inhibit leukemia proliferation

**Fig. 6:**
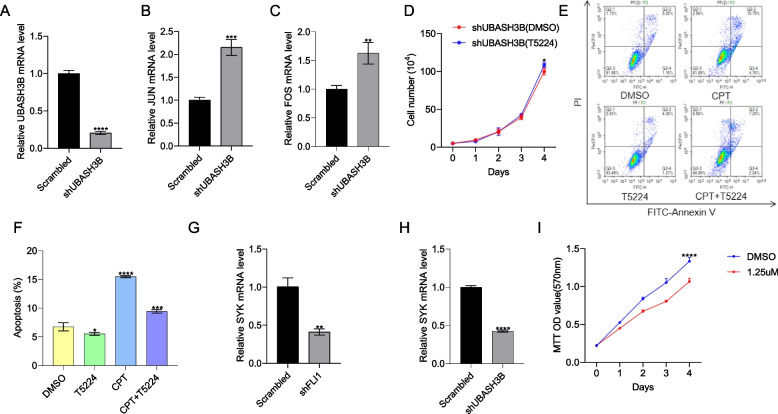
*AP1 /SYK* are regulated by *UBASH3B*. **A**-**C** Expression of *UBASH3B* (**A**), *JUN* (**B**), and *FOS* (**C**) was assessed by RT-qPCR in sh*UBASH3B* cells. **D** The proliferation of sh*UBASH3B* cells treated with the selective AP1 inhibitor T5224 (10μM) compared to vehicle-treated (DMSO) cells. **E** HEL cells were treated with 10nM camptothecin (CPT) (a FLI1 inhibitor) in combination with either DMSO or T5224 for 24 h; apoptosis was measured using flow cytometry. **F** The data is presented using the average from three experiments. **G**, **H** The expression of *SYK* in shFLI1 (**G**) and shUBASH3B (**H**) versus control cells, via RT-qPCR. **I** The proliferation of HEL cells treated with the SYK inhibitor R406 compared to vehicle-treated (DMSO). *P* < 0.05 (*), *P* < 0.01 (**), *P* < 0.001 (***), and *P* < 0.0001 (****)

The MAP Kinase pathway (Fig. 5F) heatmap revealed that *FOS* and *JUN* expression was elevated in sh*UBASH3B* knock-down cells (compared to sh*UBASH3A* and scrambled control cells), and this was further confirmed by RT-qPCR (Fig. 6A-C). The AP1 genes were also induced in shFLI1 cells (Supplemental Fig. 4B). Moreover, overexpression of FLI1 in K562 (K562-fli1) cells resulted in downregulation of both FOS and JUN (Supplemental Fig. 6A-C). Since elevated *FOS* and *JUN* expression was associated with growth suppression in shUBASH3B cells, we treated sh*UBASH3B* cells with the selective AP1 inhibitor T5224 [40], which significantly accelerated their proliferation (Fig. 6D). Moreover, T5224 significantly inhibited camptothecin (CPT, an anti-FLI1 compound [20, 41, 42])-induced HEL apoptosis in culture (Fig. 6E, F).

RNAseq analyses in shFLI1 identified drastic downregulation in expression of the spleen tyrosine kinase SYK that was also detected in shUBASH3B cells, suggesting regulation of these genes by FLI1 [36]. The *SYK* gene has been previously linked to leukemia progression [43]. Indeed, RT-qPCR analysis confirmed downregulation of SYK in shFLI1 and shUBASH3B cells (Fig. 6G, H). Treatment of HEL cells with SYK inhibitor R406 [44] significantly suppressed growth in culture (Fig. 6I). These results suggest that UBASH3B may partially exert its oncogenic activity by suppressing AP1 and activating other oncogenic factors.

### HSPA1B suppression by UBASH3A accelerates leukemia cell proliferation

**Fig. 7:**
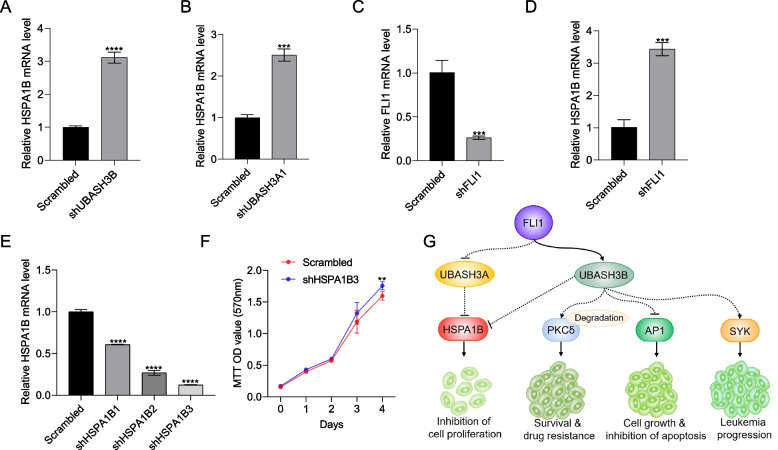
Negative regulation of the *HSPA1B* by UBASH3A controls cell proliferation. **A**, **B** Expression of HSPA1B in shUBASH3B (**A**) and shUBASH3A1 (**B**) cells, via RT-qPCR. **C**, **D** Expression of *FLI1* (**C**) and *HSPA1B* (**D**) in sh*FLI1* cells via RT-qPCR. **E** lentivirus-mediated downregulation of *HSPA1B* in HEL cells using the sh*HSPA1B*1-3 expression vector, as determined via RT-qPCR. **F** The proliferation of sh*HSPA1B*3 and scrambled control cells for the indicated days was assessed using an MTT assay. *P* < 0.01 (**), *P* < 0.001 (***). **G** Model showing the effect of *FLI1* on *UBASH3A* and *UBASH3B* expression as well as erythroleukemia progression. *UBASH3B* induction via *FLI1* overexpression suppresses PKCẟ and increased cell survival as well as drug resistance. Higher *UBASH3B* transcription following *FLI1* overexpression also causes inhibition of AP1, which would otherwise suppress leukemia progression. In addition, UBASH3B controls the expression of *SYK* and partially contributes to erythroleukemia progression. Suppression of *UBASH3A* transcription via *FLI1* overexpression increases the expression of leukemia growth suppressor *HSPA1B*, which blocks proliferation. On the other hand, activation of UBASH3B by FLI1 further decreases HSPA1B expression, causing acceleration of cell proliferation. Dotted lines represent indirect regulation

Interestingly, expression of both Heat Shock Protein Family A (Hsp70) Member 1A (*HSPA1A)* and 1B (HSPA1B) increased in sh*UBASH3A and* sh*UBASH3B* cells relative to controls (Fig. 5F). The induction of HSPA1B in shUBASH3A1 and shUBASH3B was confirmed by RT-qPCR (Fig. 7A and B). Likewise, *HSPA1B* expression was significantly induced in sh*FLI1* cells (Fig. 7C, D and Supplemental Fig. 4B). Moreover, overexpression of FLI1 in K562 (K562-fli1) cells resulted in downregulation of HSPA1B (Supplemental Fig. 6A and D) suggesting a tumor suppressor role for this gene. To determine whether *HSPA1B* is involved in UBASH3A/B mediated tumor suppression, *HSPA1B* was then knocked-down in HEL cells using three shRNA (shHSPA1B-3; Fig. 7E). The proliferation of sh*HSPA1B-*3 cells was significantly higher than in scrambled control cells (Fig. 7F). Thus, HSPA1B may mediate suppressive activity of FLI1. Since UBASH3A is induced in shFLI1, the level of HSPA1B expected to be lower causing cell growth acceleration. In contrast, lower UBASH3B expression in shFLI1 cells caused higher expression of HSPA1B, leading to growth deceleration. In the schematic in Fig. 7G, we propose that the oncogenic activity of FLI1 through UBASH3B activation may be partly mediated through AP1 suppression in erythroleukemic cells. Previously, we showed that UBASH3B upregulation increases PKCẟ degradation, which increased drug resistance and leukemia cell survival [18]. UBASH3B also activates the oncogene SYK to promote leukemia growth. Since HSPA1B is negatively regulated by both UBASH3A and UBASH3B, its tumor suppressor activity is dependent upon balance between the level of these UBASH3 proteins, negatively and positively regulated by FLI1, respectively. The balance between oncogenic and tumor suppressor activity of UBASH3B and UBASH3A, respectively, likely contributes to FLI1-induced leukemia cell proliferation.

### Correlation between FLI1 and the UBASH3A/B gene expression in other malignancies and prognostic impact

**Fig. 8:**
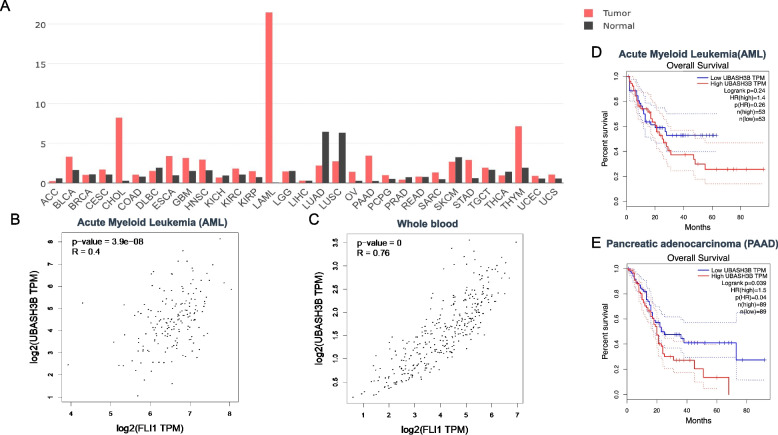
Correlation between *FLI1* and *UBASH3B* expression in various tumors. **A** Relative expression of UBASH3B in various tumor in comparison to normal cells. Adenoid Cystic Carcinoma (ACC), Bladder Urothelial Carcinoma (BLCA), Breast Invasion Carcinoma (BRCA), Cervical Squamous Cell Carcinoma and Endocervical Adenocarcinoma (CESE), Cholangiocarcinoma (CHOL), Colonadenocarcinoma (COAD), Lymphoid Neoplasm Diffuse Large B-Cell Lymphoma (DLBC), Esophageal Carcinoma (ESCA), Glioblastoma Multiform (GBM), Head and Neck Squamous Cell Carcinoma (HNSC), Kidney Chromophobe (KICH), Kidney Renal Clear Cell Carcinoma (KIRC), Kidney Renal Papillary Cell Carcinoma (KIRP), Acute Myeloid Leukemia (LAML), Brain Lower Grade Glioma (LGG), Liver Hepatocellular Carcinoma (LIHC), Lung Adenocarcinoma (LUAD), Lung Squamous Cell Carcinoma (LUSC), Ovarian Serous Cystadenocarcinoma (OV), Pancreatic Adenocarcinoma (PAAD), Pheochromocytoma and Paraganglioma (PCPG), Prostate Adenocarcinoma (PRAD), Rectum Adenocarcinoma (READ), Sarcoma (SARC), Skin Cutaneous Melanoma (SKCM), Stomach Adenocarcinoma (STAD), Testicular Germ Cell Tumors Thyroid Adenocarcinoma (TGCT), (THCA), Thymoma (THYM), Uterine Corpus Endometrial Carcinoma (UCEC), Uterine Carcinosarcoma (UCS). **B**, **C** Relative expression of human *FLI1* and *UBASH3B* in AML (**B**) and whole blood cells (**C**). **D**, **E** Overall survival rate of high and low *UBASH3B* expression in AML (**D**) and PAAD (**E**) tumors. Abbreviations n(high) and n(low) shows the number of patients in *UBASH3B* high and low expressing tumors

The aforementioned results demonstrated a positive and negative correlation between FLI1 and the *UBASH3B* and *UBASH3A* genes in erythroleukemia cell lines, respectively. To examine a broader role of these *UBASH* genes in cancer, we examined the correlation between FLI1 and UBASH3A or UBASH3B in the TCGA database by GEPIA2. In most tumors, expression analysis revealed a higher level of UBASH3B versus normal samples (Fig. 8A). In Acute Myeloid Leukemia (AML) and whole blood cells, the expression of *FLI1* was significantly correlated with the level of *UBASH3B* (Fig. 8B, C). Higher *UBASH3B* in AML, Pancreatic Adenocarcinoma, Brain lower grade glioma, Pancreatic adenocarcinoma and Lung squamous cell carcinoma were also correlated with worse prognosis (Fig. 8D, E and Supplementary Fig. 7A-C). These results further support the oncogenic function of UBASH3B in different tumors.

**Fig. 9:**
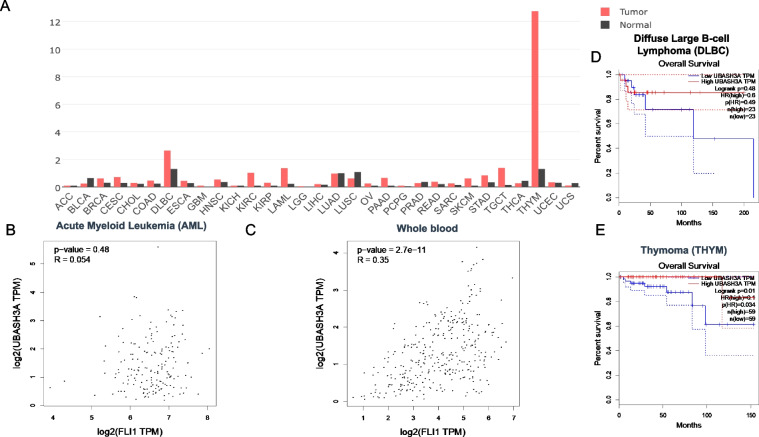
Correlation between *FLI1* and *UBASH3A* expression in various tumors. **A** Relative expression of *UBASH3A* in the indicated tumors in comparison to normal cells. **B**, **C** Relative expression of human *FLI1* and *UBASH3A* in AML (**B**) and whole blood cells (**C**). **D**, **E** Overall survival rate of high and low expression of *UBASH3A* in DLBC (**D**) and PAAD (**E**) tumors

A positive and negative correlations between FLI1 and UBASH3A were observed in various tumors (Fig. 9A). Interestingly, positive correlation between *FLI1* and *UBASH3A* were seen in AML and whole blood cells (Fig. 9B and C). However, higher expression of *UBASH3A* had a better prognosis outcome in diffuse large B-cell lymphoma, Breast invasive Carcinoma, Colon adenocarcinoma, Head and neck squamous cell carcinoma, Liver hepatocellular carcinoma and Skin cutaneous melanoma (Fig. 9D and Supplementary Fig. 8A-E). Thymoma was the only tumor in which higher *UBASH3A* was significantly associated with worse patient outcome (Fig. 9E). These results suggest a tumor specific dependent suppressor function for *UBASH3A*.

## Discussion

The ETS oncogene FLI1 is a major driver of tumor initiation and progression of diverse types of malignancies [38]. FLI1 regulated genes have been identified to control various cancer hallmarks including cell proliferation, differentiation, apoptosis, genomic stability, and immunity [37]. The combined effect of these downstream effectors contributes to robust oncogenic activity associated with FLI1 overexpression. Herein, we show that both *UBASH3A* and *UBASH3B* are strong downstream targets of FLI1. *UBASH3B* was found to be a direct target of FLI1, and its activation promotes erythroleukemia growth. In contrast, *UBASH3A* is indirectly downregulated by FLI1 through GATA2 or possibly other transcription factors and likely acts as an inhibitor of erythroleukemic cell proliferation. RNAseq analysis identified distinct and overlapping downstream pathways for UBASH3A and UBASH3B that likely contribute to their suppressive and oncogenic activity, respectively. This study provides novel insights into the role of these factors in leukemia progression.

In Acute Myeloid Leukemia (AML) induced by the oncogene AML-ETO, UBASH3B inactivates CBL, which is predicted to inhibit the ubiquitination of its downstream effectors responsible for leukemogenesis [16]. Similarly, in triple negative breast cancer, higher expression of UBASH3B promotes dephosphorylation and inactivation of CBL, which in turn loses ability to ubiquitinate and induce degradation of the epidermal growth factor receptor (EGFR), leading to accelerated cancer progression [17]. We also previously identified PKCẟ as one of its downstream targets of UBASH3B [18]. Interaction between UBASH3B and PKCẟ accelerated ubiquitination of this kinase, resulting in leukemia cell survival and drug resistance. Moreover, a positive correlation between *FLI1/UBASH3B* was observed in several cancer types associated with worse prognosis. These results confirm oncogenic activity of UBASH3B in erythroleukemia and likely other cancers.

In a previous study [45], we reported regulation of FOS and JUN by FLI1 in leukemic cells. Herein, we showed that loss of FLI1 and consequently its downstream target UBASH3B in leukemia cells increased AP1 expression, leading to proliferation suppression and increased apoptosis. While AP1 is shown here to function as a tumor suppressor gene downstream of UBASHB, this transcription factor is also known to function as an oncogene in various cancers [46]. Like TGF signaling, the AP1 function in cancer could go both ways [47]. In our study, AP1(FOS and JUN) expression is negatively regulated during leukemia progression. Indeed, JUNB and JUNA are found critical downstream effectors of the tumor suppressor activity of another ETS gene family SPI1/PU.1, and that reduced expression of JUNB shown to be a common feature of acute myeloid leukemogenesis [48]. Since FLI1 knockdown or overexpressing cells exhibit increased or decreased expression of the *AP1* genes, respectively [45], we propose a tumor suppressor role for AP1 in erythroleukemia. In addition to AP1, we identified the activation of the *SYK* gene by FLI1 through UBASH3B. Dephosphorylation of SYK and SAP70 by UBASH3B, two main factors involved in TCR signaling, was previously reported [3, 14, 15]. However, SYK kinase activation is also implicated in leukemia progression [43]. Thus, SYK activation likely contributed to the oncogenic activity of FLI1 through UBASH3B. The mechanisms by which UBASH3B suppresses AP1 transcription and activates SYK has yet to be determined. However, the interaction between UBASH3B and CBL or downregulation of PKCẟ may modify FOS/JUN and SYK regulation. This notion remains to be investigated in future studies.

Despite critical involvement in autoimmunity, the connection between UBASH3A and cancer has not yet been established. In contrast to *UBASH3B*, knockdown of FLI1 in erythroleukemia cells upregulates *UBASH3A* expression, raising the possibility of a tumor suppressor function for this variant. In support of this observation, ablation of UBASH3A in high FLI1 expressing erythroleukemic cells significantly accelerated cell proliferation in culture. Interestingly, UBASH3A expression was both induced and reduced relative to normal cells in various cancers. However, higher expression of UBASH3A was found to be a good prognosis marker for patient survival in most tumors, further supporting its anti-cancer activity. FLI1 indirectly controls the transcription of UBASH3A, likely through GATA2, which may warrant further investigation in future studies.

RNAseq analysis of UBASH3A and UBASH3B knocked-down cells revealed the highest effects on the MAP Kinase pathway. Specifically, expression of *HSPA1A* and *HSPA1B* increased in both shUBASH3B and shUBASH3A cells. Knockdown of HSPA1B in leukemia cells accelerated leukemogenesis indicating a role for these genes as negative regulators of leukemic cell growth. Interestingly, higher *HSPA1A* and *HSPA1B* expression was previously linked to poor survival in colon cancer. In hepatocellular carcinoma (HCC), expression of HSPA1B increased through Hepatitis B virus-mediated activation of ATF7, which accelerated cell proliferation by inhibiting apoptosis [49]. In contrast to solid tumors, the data presented herein suggest an inhibitory role for *HSPA1B* in leukemia progression, whose expression depend upon the level of UBASH3A and UBASH3B.

Finally, *UBASH3A* and *UBASH3B* knockdown affected similar as well as unique genes, as shown here for AP1, SYK and HSPA1B. Thus, the combined oncogenic and tumor suppressor activities of UBASH3A and UBASH3B and their downstream effectors influence leukemogenesis. Examining other genes regulated by UBASH3A and UBASH3B could further determine their role in leukemogenesis, and uncover additional therapeutic targets.

## Conclusions

FLI1 is shown in this study to promote erythroleukemia progression by inhibiting *UBASH3A* and expression and inducing *UBASH3B* expression. UBASH3B acts as an oncogene to block the AP1 pathway and activate other genes, whereas *UBASH3A* transcriptional inactivation by FLI1 suppressed expression of *HSPA1B*. These results uncover critical roles of UBASH3A and UBASH3B in FLI1-driven leukemias.

### Supplementary Information

**Supplementary Materials 1.**

**Supplementary Materials 2.**

**Supplementary Materials 3.**

## Data Availability

The datasets generated and/or analysed during the current study are available in the Sequence Read Archive (SRA) repository, https://www.ncbi.nlm.nih.gov/bioproject/1014802.

## Abbreviations

FLI1: Friend leukemia integration 1
UBASH3A: Ubiquitin Associated and SH3 Domain Containing A
UBASH3B: Ubiquitin Associated and SH3 Domain Containing B
SYK: Spleen tyrosine kinase
HSPA1B: Heat shock protein family A (Hsp70) member 1B
ChIP: Chromatin immunoprecipitation
KEGG: Kyoto Encyclopedia of Genes and Genomes
DMSO: Dimethyl sulfate
PKC: Protein Kinase C
AP1: Activator protein 1
MAPK: Mitogen-Activated Protein Kinase
PGM: Phosphoglycerate mutase-like/C-terminal histidine phosphatase
