# Supplementary material for: FLI1 induces erythroleukemia through opposing effects on UBASH3A and UBASH3B expression
Source: BMC Cancer. 2024 Mar 9;24:326. doi: 10.1186/s12885-024-12075-2 (PMC10925000; doi:10.1186/s12885-024-12075-2)
Supplement: Supplementary file 1 — Supplementary Materials 1. [file 12885_2024_12075_MOESM1_ESM.pdf]

Supplemental Table 1. Differential expression of genes in Scrambled and shUBASH3A1

	Gene ID	Gene Symbol	Scrambled FPKM	shUBASH3A FPKM	log2FoldChange	Padj
1	8363	H4C11	0	5.83	9.1873521	0.002460748
2	100529063	BCL2L2-PABPN1	0	4.56	8.83289	5.60E-36
3	58530	LY6G6D	0	3.92	8.6147098	0.014928517
4	632	BGLAP	0	2.6	8.0223678	0.008165034
5	6757	SSX2	0	1.27	6.9886847	9.48E-06
6	728689	EIF3CL	0	1.1	6.7813597	5.31E-11
7	102723680	CT45A9	0	0.82	6.357552	0.001345342
8	1159	CKMT1B	0	0.8	6.3219281	1.14E-04
9	390066	OR52D1	0	0.65	6.0223678	0.01492568
10	24150	TP53TG3	0	0.46	5.523562	0.002460174
11	2826	CCR10	0	0.43	5.4262648	0.004495456
12	106821730	BUB1B-PAK6	0	0.39	5.2854022	5.08E-06
13	1179	CLCA1	0	0.38	5.2479275	1.14E-04
14	347051	SLC10A5	0	0.33	5.0443941	0.048313502
15	374907	B3GNT8	0	0.32	5	0.002461323
16	359	AQP2	0.02	0.51	4.6724253	1.28E-07
17	140711	TLDC2	0	0.22	4.4594316	0.048290144
18	100526832	PHOSPHO2-KLHL23	0	0.2	4.3219281	0.001345007
19	84699	CREB3L3	0	0.17	4.0874628	0.048297928
20	64850	ETNPPL	0.04	0.56	3.8073549	2.85E-04
21	353376	TICAM2	0	0.14	3.8073549	0.048305714
22	102724219	LOC102724219	0.21	2.78	3.7266237	4.78E-38
23	163732	CITED4	0.06	0.62	3.3692338	0.023551908
24	92211	CDHR1	0.01	0.1	3.3219281	0.023543452
25	280658	SSX7	0.07	0.69	3.3011695	0.013867209
26	115827	RAB3C	0.02	0.19	3.2479275	1.31E-04
27	56001	NXF2	0.03	0.28	3.2223924	0.03948774
28	85319	BAGE2	0.11	1.02	3.2129937	2.98E-05
29	201633	TIGIT	0.01	0.09	3.169925	5.52E-07
30	90506	LRRC46	0.08	0.68	3.0874628	0.00226723
31	102724473	GAGE10	0.69	5.57	3.0130091	3.69E-04
32	4744	NEFH	0.98	7.71	2.9758772	1.38E-68
33	11189	CELF3	0.06	0.42	2.8073549	3.41E-07
34	7757	ZNF208	0.01	0.07	2.8073549	0.023547679
35	5649	RELN	0.03	0.18	2.5849625	7.35E-05
36	7373	COL14A1	0	0.06	2.5849625	0.027039228
37	3303	HSPA1A	12.43	74.56	2.5845756	0
38	2348	FOLR1	0.41	2.45	2.5790859	4.50E-04
39	29106	SCG3	0.07	0.38	2.4405726	0.007168977
40	26872	STEAP1	0.14	0.75	2.4214638	0.044022265
41	79541	OR2A4	0.14	0.73	2.3824696	0.007167499
42	346673	STRA8	0.12	0.62	2.3692338	0.027528884
43	3050	HBZ	101.47	515.62	2.345255	0
44	64478	CSMD1	0.03	0.15	2.3219281	0.001192952
45	92558	BICDL1	0.11	0.54	2.2954559	0.004947172
46	10683	DLL3	0.08	0.39	2.2854022	0.044015038
47	28984	RGCC	0.68	3.29	2.2744809	9.14E-05
48	3399	ID3	1.78	8.55	2.2640472	4.12E-11
49	728215	FAM155A	0.1	0.48	2.2630344	3.86E-05
50	339456	TMEM52	1.31	6.22	2.2473478	5.06E-08
51	7022	TFAP2C	0.1	0.47	2.2326608	0.007569883
52	54913	RPP25	0.19	0.89	2.2278059	5.44E-04
53	101059906	LOC101059906	0.11	0.51	2.2129937	0.018287264
54	2104	ESRRG	0.05	0.23	2.2016339	0.004465517
55	347735	SERINC2	0.12	0.51	2.0874628	0.044368745
56	445582	POTEE	0.17	0.72	2.0824622	1.53E-05
57	84528	RHOXF2	1.89	7.64	2.0151864	4.72E-32
58	57520	HECW2	0.02	0.08	2	0.018283872

59	54970	TTC12	0.22	0.87	1.9835119	0.004705637
60	51083	GAL	15.88	61.63	1.9564219	1.75E-44
61	8927	BSN	0.15	0.58	1.9510904	3.24E-14
62	28999	KLF15	0.12	0.46	1.9385995	0.04255609
63	256227	STEAP1B	0.59	2.25	1.9311381	3.61E-04
64	11187	PKP3	1.95	7.35	1.9142701	3.59E-28
65	113835	ZNF257	0.11	0.41	1.8981204	0.007394137
66	644815	FAM83G	0.31	1.15	1.8912937	6.68E-09
67	50512	PODXL2	2.11	7.78	1.8825272	7.11E-22
68	793	CALB1	0.45	1.64	1.8656989	4.65E-06
69	197342	EME2	0.72	2.62	1.863498	1.52E-04
70	3748	KCNC3	0.51	1.84	1.8511366	1.55E-16
71	92421	CHMP4C	0.17	0.61	1.8432745	0.042563115
72	3040	HBA2	389.37	1396.65	1.8427569	0
73	79918	SETD6	0.6	2.14	1.8345764	9.08E-05
74	84253	GARNL3	0.09	0.32	1.830075	0.028176443
75	351	APP	1.23	4.37	1.828975	2.12E-14
76	7164	TPD52L1	1.54	5.44	1.8206763	6.53E-06
77	4113	MAGEB2	1	3.51	1.811471	4.82E-07
78	55287	TMEM40	0.14	0.49	1.8073549	0.027533722
79	157285	PRAG1	0.12	0.42	1.8073549	0.001994658
80	1903	S1PR3	0.4	1.39	1.797013	2.28E-08
81	107987373	LOC107987373	6.44	22.01	1.7730266	6.05E-14
82	27242	TNFRSF21	0.52	1.77	1.7671658	3.38E-06
83	166929	SGMS2	0.08	0.27	1.7548875	0.007025075
84	26468	LHX6	0.33	1.11	1.7500217	1.19E-04
85	8313	AXIN2	0.09	0.3	1.7369656	0.026732153
86	4072	EPCAM	1.58	5.24	1.7296423	3.34E-09
87	116211	TM4SF19	0.4	1.32	1.722466	0.042549068
88	645051	GAGE13	12.8	42.12	1.7183616	8.20E-15
89	6899	TBX1	0.77	2.51	1.704757	3.24E-06
90	57477	SHROOM4	0.08	0.26	1.7004397	0.005557601
91	7368	UGT8	0.34	1.09	1.6807215	5.85E-06
92	10953	TOMM34	10.22	32.72	1.6787776	7.93E-69
93	55150	C19orf73	0.86	2.75	1.6770231	0.024795113
94	23678	SGK3	0.27	0.86	1.6713773	5.43E-05
95	116832	RPL39L	2.49	7.92	1.6693547	4.74E-05
96	10893	MMP24	0.34	1.08	1.6674247	2.64E-06
97	112268271	LOC112268271	0.06	0.19	1.662965	0.016735658
98	5621	PRNP	0.96	3.03	1.6582115	1.53E-08
99	79605	PGBD5	0.21	0.66	1.6520767	2.77E-09
100	641455	POTEM	0.07	0.22	1.6520767	0.022656893
101	102723631	CT45A10	2.28	7.09	1.6367518	2.43E-08
102	55630	SLC39A4	12	37.13	1.6295509	8.35E-20
103	110354863	ZNF660-ZNF197	0.54	1.67	1.6288168	7.51E-22
104	8187	ZNF239	0.45	1.39	1.627088	2.84E-04
105	956	ENTPD3	0.14	0.43	1.6189098	0.011881103
106	25907	TMEM158	7.05	21.48	1.6072988	5.97E-39
107	27440	HDHD5	2.03	6.17	1.6037908	1.96E-11
108	5575	PRKAR1B	1.43	4.32	1.5950162	1.97E-11
109	114108587	ATF7-NPFF	0.49	1.48	1.5947435	0.003828406
110	23413	NCS1	0.51	1.54	1.5943612	2.06E-09
111	7166	TPH1	0.1	0.3	1.5849625	0.032859222
112	80070	ADAMTS20	0.09	0.27	1.5849625	0.014164299
113	7227	TRPS1	0.08	0.24	1.5849625	0.002413872
114	7262	PHLDA2	2.79	8.35	1.5815111	9.93E-07
115	120071	LARGE2	0.49	1.46	1.5751147	3.13E-04
116	55600	ITLN1	17.64	52.34	1.5690634	5.91E-54
117	84634	KISS1R	2.78	8.24	1.5675595	6.34E-13
118	2100	ESR2	0.19	0.56	1.5594274	0.032507286
119	9615	GDA	0.66	1.94	1.5555187	2.99E-07

120	130340	AP1S3	0.16	0.47	1.5545889	0.012845651
121	5727	PTCH1	0.16	0.47	1.5545889	0.002770947
122	79887	PLBD1	1.03	3	1.5423182	3.38E-06
123	268	AMH	0.39	1.13	1.5347767	0.012843162
124	547	KIF1A	2.08	6.01	1.5307815	4.06E-57
125	2260	FGFR1	0.17	0.49	1.527247	0.004090594
126	5744	PTHLH	0.25	0.72	1.5260688	0.028171503
127	282763	OR51B5	1.36	3.91	1.523562	1.23E-05
128	389432	SAMD5	0.59	1.69	1.5182364	1.16E-11
129	81552	VOPP1	1.24	3.55	1.5174789	8.23E-11
130	4685	NCAM2	0.6	1.71	1.5109619	1.70E-04
131	100533467	BIVM-ERCC5	0.53	1.51	1.5104843	1.83E-08
132	643988	FNDC10	0.72	2.04	1.5025003	1.12E-04
133	10332	CLEC4M	0.6	1.7	1.5025003	0.001265603
134	221711	SYCP2L	0.36	1.02	1.5025003	0.00126592
135	23034	SAMD4A	0.12	0.34	1.5025003	0.006453695
136	142683	ITLN2	4.07	11.5	1.4985332	2.21E-11
137	23224	SYNE2	0.34	0.96	1.4974997	4.12E-04
138	57211	ADGRG6	0.11	0.31	1.4947647	0.007209423
139	1829	DSG2	1.36	3.79	1.4785912	1.28E-20
140	8412	BCAR3	0.18	0.5	1.4739312	0.029423595
141	83987	CCDC8	0.84	2.33	1.4718687	8.69E-08
142	10360	NPM3	25.82	71.5	1.4694542	9.66E-44
143	730394	GTF2H2C_2	2.51	6.94	1.4672483	6.65E-11
144	145282	MIPOL1	0.17	0.47	1.467126	0.004452168
145	54734	RAB39A	0.46	1.27	1.4651227	0.007207938
146	254528	MEIOB	3.95	10.9	1.4644036	3.47E-18
147	29948	OSGIN1	0.89	2.45	1.4609045	9.13E-05
148	4585	MUC4	0.04	0.11	1.4594316	0.007188376
149	7291	TWIST1	0.69	1.89	1.453718	0.002577578
150	286077	FAM83H	1.44	3.94	1.4521268	1.03E-21
151	729428	GAGE12B	53.59	146.21	1.4480063	2.14E-27
152	55605	KIF21A	1.22	3.32	1.4443021	3.55E-20
153	246721	POLR2J2	1.76	4.73	1.4262648	1.84E-08
154	2543	GAGE1	34.34	92.18	1.4245637	1.63E-28
155	6583	SLC22A4	0.44	1.18	1.4232114	0.012933174
156	221002	RASGEF1A	1.06	2.84	1.4218267	9.72E-08
157	7205	TRIP6	4.15	11.11	1.4206756	9.38E-16
158	10874	NMU	12.99	34.68	1.4167025	5.68E-18
159	2195	FAT1	0.26	0.69	1.4080847	4.10E-10
160	79969	ATAT1	0.34	0.9	1.4043903	0.039246241
161	8612	PLPP2	1.13	2.99	1.4038227	0.001837649
162	3039	HBA1	1673.45	4418.79	1.4008259	0
163	666	BOK	0.3	0.79	1.3968902	0.018422047
164	57105	CYSLTR2	11.54	30.38	1.3964786	3.21E-127
165	6640	SNTA1	1	2.63	1.3950628	1.48E-05
166	284358	MAMSTR	0.59	1.55	1.3934814	0.035213263
167	8153	RND2	0.16	0.42	1.3923174	0.026194858
168	284129	SLC26A11	0.21	0.55	1.3890423	0.022660979
169	11135	CDC42EP1	4.58	11.99	1.3884122	1.49E-21
170	115290	FBXO17	0.83	2.17	1.3865118	1.04E-04
171	642778	NPIPA3	2.67	6.98	1.3863873	5.20E-13
172	79012	CAMKV	0.23	0.6	1.3833286	0.02619022
173	10045	SH2D3A	0.51	1.33	1.3828571	0.005767774
174	27141	CIDEB	3.38	8.81	1.3821188	2.29E-11
175	1690	COCH	3.86	10.04	1.3790865	2.86E-23
176	23428	SLC7A8	2.57	6.66	1.3737538	1.32E-17
177	3293	HSD17B3	1.69	4.37	1.37061	4.63E-05
178	388722	C1orf53	3.06	7.91	1.370146	0.004570392
179	220992	ZNF485	0.45	1.16	1.3661279	0.005608911
180	50940	PDE11A	0.07	0.18	1.3625701	0.026185584

181	131540	ZDHHC19	0.96	2.45	1.3516754	0.012935677
182	56675	NRIP3	0.49	1.25	1.3510744	5.17E-05
183	6297	SALL2	0.69	1.76	1.3509072	1.15E-07
184	91522	COL23A1	0.55	1.4	1.3479233	4.12E-04
185	440590	ZYG11A	0.57	1.45	1.3470191	4.74E-06
186	1595	CYP51A1	3.61	9.15	1.3417729	7.79E-24
187	54478	PIMREG	0.53	1.34	1.3381687	0.011416532
188	55228	PNMA8A	1.22	3.08	1.3360492	6.45E-10
189	134549	SHROOM1	2.75	6.92	1.3313404	1.68E-18
190	150221	RIMBP3C	0.1	0.25	1.3219281	0.03286487
191	729447	GAGE2A	49.92	124.41	1.3174126	2.39E-28
192	187	APLNR	1.69	4.21	1.316797	1.20E-13
193	126626	GABPB2	0.51	1.27	1.3162593	0.042091437
194	222234	FAM185A	0.51	1.27	1.3162593	0.005145813
195	79746	ECHDC3	0.99	2.46	1.3131579	0.002442021
196	2318	FLNC	2.45	6.08	1.3112896	7.43E-47
197	94122	SYTL5	0.27	0.67	1.3112017	0.002280113
198	114132	SIGLEC11	0.21	0.52	1.3081223	0.04675624
199	54898	ELOVL2	0.38	0.94	1.3066613	0.002933759
200	63917	GALNT11	1.4	3.46	1.3053452	9.23E-08
201	1675	CFD	1.53	3.78	1.3048546	0.001129631
202	100996928	FMC1-LUC7L2	3.67	9.04	1.3005427	1.61E-19
203	7133	TNFRSF1B	1.08	2.66	1.3003949	4.28E-08
204	388591	RNF207	0.24	0.59	1.2976805	0.011418774
205	51260	PBDC1	2.57	6.31	1.2958716	1.42E-05
206	225689	MAPK15	0.38	0.93	1.2912313	0.01642121
207	653067	XAGE1B	2.29	5.59	1.2875007	0.02265281
208	23753	SDF2L1	36.74	89.6	1.2861471	9.08E-41
209	84258	SYT3	0.57	1.39	1.2860511	0.001282878
210	107984449	LOC107984449	0.55	1.34	1.2847295	7.70E-04
211	126075	CCDC159	1.45	3.53	1.2836153	0.001449306
212	23026	MYO16	0.53	1.29	1.2833068	7.66E-08
213	4199	ME1	2.64	6.42	1.2820354	2.44E-15
214	79948	PLPPR3	0.63	1.53	1.2801079	0.001449665
215	9027	NAT8	0.82	1.98	1.2718046	0.044840734
216	5241	PGR	0.22	0.53	1.2684888	3.34E-06
217	9659	PDE4DIP	13.88	33.41	1.2672724	6.25E-41
218	1645	AKR1C1	4.2	10.1	1.2658941	1.16E-37
219	255738	PCSK9	7.28	17.49	1.2645199	3.95E-48
220	3046	HBE1	22.53	53.74	1.254149	3.87E-15
221	105379417	LOC105379417	0.39	0.93	1.2537566	0.036821221
222	645073	GAGE12G	27.04	64.34	1.2506208	4.68E-15
223	121355	GTSF1	21.43	50.98	1.2502995	1.16E-21
224	138428	PTRH1	2.59	6.16	1.2499783	2.26E-04
225	2519	FUCA2	4.16	9.86	1.2450041	5.43E-17
226	56159	TEX11	2.2	5.2	1.2410081	8.44E-13
227	9256	TSPOAP1	0.22	0.52	1.2410081	7.66E-04
228	139135	PASD1	0.64	1.51	1.2384047	3.78E-04
229	202243	CCDC125	0.94	2.2	1.2267709	5.28E-06
230	23322	RPGRIP1L	0.71	1.66	1.2252923	0.01853975
231	6795	AURKC	0.93	2.17	1.2223924	0.03520727
232	4703	NEB	0.06	0.14	1.2223924	7.16E-04
233	10815	CPLX1	2.84	6.59	1.2143875	8.37E-10
234	323	APBB2	0.44	1.02	1.2129937	1.64E-04
235	727940	RHOXF2B	4.6	10.66	1.2125017	1.26E-08
236	22843	PPM1E	0.19	0.44	1.2115041	0.009586954
237	2000	ELF4	1.12	2.59	1.2094534	2.37E-07
238	1728	NQO1	4.62	10.64	1.2035334	4.85E-18
239	668	FOXL2	1.37	3.15	1.2011759	6.38E-07
240	59277	NTN4	0.64	1.47	1.1996723	1.96E-04
241	4897	NRCAM	1.13	2.59	1.1966293	7.86E-13

242	26010	SPATS2L	2.55	5.84	1.1954711	9.45E-09
243	27077	B9D1	0.97	2.22	1.194503	0.040455062
244	114793	FMNL2	0.74	1.69	1.1914261	2.09E-07
245	84617	TUBB6	18.86	43.07	1.1913536	7.21E-50
246	1958	ERG1	28.75	65.46	1.1870516	5.35E-138
247	7580	ZNF32	1.06	2.41	1.1849689	0.01391072
248	57713	SFMBT2	0.3	0.68	1.1805722	1.50E-04
249	56999	ADAMTS9	0.36	0.81	1.169925	6.68E-05
250	5169	ENPP3	3	6.74	1.1677861	2.48E-14
251	7082	TJP1	0.74	1.66	1.1655861	1.03E-09
252	203859	ANO5	0.37	0.83	1.1655861	2.04E-04
253	27443	CECR2	0.33	0.74	1.1650592	2.22E-05
254	220929	ZNF438	0.59	1.32	1.1617511	0.0124293
255	2017	CTTN	6.25	13.92	1.1552311	2.45E-26
256	5010	CLDN11	2.21	4.92	1.1546119	3.14E-09
257	4920	ROR2	1.79	3.98	1.1528088	7.91E-14
258	3170	FOXA2	0.94	2.09	1.1527703	6.43E-04
259	1016	CDH18	0.63	1.4	1.1520031	1.47E-04
260	57698	SHTN1	1.13	2.51	1.1513646	2.93E-08
261	6723	SRM	110.29	244.85	1.1505962	6.97E-166
262	348645	C22orf34	0.82	1.82	1.1502426	5.73E-04
263	140690	CTCFL	6.15	13.64	1.1491853	3.76E-23
264	3856	KRT8	16.97	37.63	1.1488967	9.82E-40
265	5802	PTPRS	1.24	2.74	1.1438358	8.12E-14
266	65268	WNK2	0.44	0.97	1.1404812	6.73E-06
267	54873	PALMD	1.98	4.35	1.135515	8.40E-07
268	64856	VWA1	0.21	0.46	1.1312445	0.040461796
269	727837	SSX2B	1.12	2.45	1.129283	0.014548264
270	3304	HSPA1B	31.6	69	1.1266718	8.40E-105
271	400916	CHCHD10	89.88	195.95	1.1244135	5.01E-55
272	51700	CYB5R2	2.28	4.97	1.124212	0.00198757
273	84261	FBXW9	3.17	6.9	1.1221135	3.22E-07
274	4053	LTBP2	0.3	0.65	1.1154772	3.28E-04
275	57085	AGTRAP	5.22	11.28	1.1116454	3.70E-07
276	348	APOE	70.25	151.56	1.1093189	1.82E-88
277	1982	EIF4G2	108.17	232.92	1.1065341	0
278	3858	KRT10	9.57	20.58	1.1046522	1.28E-25
279	55502	HES6	14.14	30.32	1.1004876	7.20E-22
280	65263	PYCR3	3.36	7.18	1.0955226	9.85E-15
281	79339	OR51B4	2.74	5.85	1.0942607	0.003147524
282	163154	PRR22	0.85	1.81	1.090455	0.046858298
283	8572	PDLIM4	1.53	3.24	1.0824622	1.07E-04
284	7923	HSD17B8	1.9	4.02	1.0811961	0.01497693
285	4301	AFDN	2.02	4.26	1.0764981	1.14E-20
286	91523	PCED1B	3.8	8	1.0740006	4.81E-10
287	9096	TBX18	1.21	2.54	1.0698214	9.02E-05
288	8091	HMGA2	2.32	4.86	1.0668315	3.33E-08
289	89874	SLC25A21	0.95	1.99	1.066769	0.006960269
290	145781	GCOM1	0.22	0.46	1.0641303	0.019106651
291	55805	LRP2BP	0.22	0.46	1.0641303	0.017109409
292	10553	HTATIP2	5.26	10.96	1.0591131	4.54E-08
293	653220	XAGE1A	29.94	62.25	1.0559996	8.43E-09
294	102724231	C3orf86	3.47	7.21	1.0550636	0.005524984
295	655	BMP7	0.39	0.81	1.0544478	0.009735833
296	80303	EFHD1	1.52	3.15	1.0512805	4.34E-04
297	26270	FBXO6	1.66	3.44	1.0512253	0.005463119
298	5578	PRKCA	0.83	1.72	1.0512253	1.54E-09
299	6708	SPTA1	2.64	5.47	1.0510029	3.50E-26
300	3880	KRT19	15.06	31.19	1.0503618	2.43E-21
301	10675	CSPG5	0.57	1.18	1.049753	0.035369836
302	2247	FGF2	0.29	0.6	1.0489096	0.002369635

303	81848	SPRY4	1.03	2.13	1.0482091	1.03E-06
304	10321	CRISP3	3.25	6.7	1.0437214	2.99E-08
305	54587	MXRA8	1.21	2.49	1.0411387	0.002158932
306	9143	SYNGR3	3.79	7.79	1.0394255	9.71E-09
307	56288	PARD3	1.32	2.71	1.0377549	4.51E-06
308	150223	YDJC	55.78	114.17	1.0333638	3.88E-60
309	347733	TUBB2B	0.88	1.8	1.0324215	0.012992565
310	63035	BCORL1	0.27	0.55	1.0264722	0.002738216
311	92840	REEP6	48.95	99.63	1.0252714	1.67E-62
312	79366	HMGN5	17.07	34.71	1.0238883	6.87E-37
313	114804	RNF157	0.85	1.72	1.0168738	3.35E-05
314	378708	CENPS	9.29	18.74	1.0123705	2.12E-07
315	200879	LIPH	2.94	5.89	1.0024515	2.16E-11
316	84883	AIFM2	1.24	2.48	1	6.09E-05
317	653269	POTEI	0.17	0.34	1	0.046531213
318	121268	RHEBL1	7.6	3.8	-1	1.17E-04
319	81794	ADAMTS10	1.32	0.66	-1	0.002879208
320	57524	CASKIN1	0.8	0.4	-1	0.011336306
321	57480	PLEKHG1	0.34	0.17	-1	0.033948014
322	9379	NRXN2	7.21	3.6	-1.002002	2.80E-20
323	126014	OSCAR	4.05	2.02	-1.003567	1.07E-04
324	26051	PPP1R16B	16.52	8.23	-1.005249	1.78E-56
325	5629	PROX1	1.43	0.71	-1.010124	4.95E-07
326	143162	FRMPD2	1.17	0.58	-1.012384	0.049325482
327	57835	SLC4A5	1.07	0.53	-1.013547	0.006482409
328	25791	NGEF	2.04	1.01	-1.014214	0.004616914
329	79187	FSD1	11.15	5.52	-1.014304	1.30E-10
330	10867	TSPAN9	19.05	9.43	-1.014461	1.30E-44
331	114783	LMTK3	1.54	0.76	-1.018859	4.49E-05
332	339327	ZNF546	1.54	0.76	-1.018859	1.28E-07
333	8302	KLRC4	2.76	1.36	-1.021062	0.048219075
334	10809	STARD10	10.01	4.93	-1.021782	1.06E-10
335	26579	MYEOV	65.53	32.27	-1.021962	2.52E-82
336	2206	MS4A2	4.41	2.17	-1.023084	2.65E-08
337	2701	GJA4	6.77	3.33	-1.023634	4.76E-06
338	388536	ZNF790	1.83	0.9	-1.023847	0.001567682
339	3695	ITGB7	4.01	1.97	-1.025407	1.26E-06
340	80763	SPX	16.53	8.11	-1.027313	4.68E-20
341	102	ADAM10	94.47	46.25	-1.030403	7.03E-275
342	6503	SLA	4.23	2.07	-1.031027	2.04E-07
343	55890	GPRC5C	2.21	1.08	-1.033015	0.011870379
344	941	CD80	1.72	0.84	-1.033947	0.002358482
345	2115	ETV1	9.96	4.86	-1.035189	3.63E-27
346	93589	CACNA2D4	6.98	3.4	-1.037692	2.72E-22
347	2047	EPHB1	2.71	1.32	-1.037755	7.86E-08
348	348094	ANKDD1A	2.32	1.13	-1.037802	1.04E-04
349	7473	WNT3	1.87	0.91	-1.0391	3.93E-04
350	30817	ADGRE2	15.31	7.45	-1.039162	9.42E-50
351	56901	NDUFA4L2	5.2	2.53	-1.039374	0.001105993
352	145864	HAPLN3	27.36	13.26	-1.044987	1.80E-27
353	3430	IFI35	8.72	4.22	-1.047085	1.17E-05
354	6571	SLC18A2	22.43	10.81	-1.053063	2.12E-49
355	64098	PARVG	28.34	13.62	-1.057113	1.63E-28
356	7305	TYROBP	12.62	6.05	-1.060705	0.00274731
357	3990	LIPC	46.25	22.16	-1.061495	1.35E-55
358	54498	SMOX	18.08	8.63	-1.066962	5.65E-21
359	84502	JPH4	2.18	1.04	-1.067745	2.50E-06
360	285852	TREML4	1.95	0.93	-1.068172	0.001451167
361	58472	SQOR	1.7	0.81	-1.069541	0.018509149
362	28514	DLL1	1.47	0.7	-1.070389	4.13E-04
363	8997	KALRN	36.21	17.24	-1.070628	1.65E-77

364	283248	RCOR2	3.93	1.87	-1.071491	2.51E-07
365	26960	NBEA	5.26	2.5	-1.073135	1.47E-11
366	10501	SEMA6B	2.76	1.31	-1.075101	3.51E-07
367	23109	DDN	3.9	1.85	-1.075949	2.72E-09
368	79966	SCD5	6.79	3.22	-1.076351	5.94E-12
369	342184	FMN1	0.55	0.26	-1.08092	9.59E-05
370	974	CD79B	3.96	1.87	-1.082462	5.01E-04
371	26047	CNTNAP2	0.36	0.17	-1.082462	3.57E-05
372	8854	ALDH1A2	1.95	0.92	-1.083768	6.70E-05
373	597	BCL2A1	4.06	1.91	-1.087907	0.032976146
374	10791	VAMP5	6.74	3.17	-1.088266	0.0152608
375	2041	EPHA1	13.23	6.2	-1.093473	8.88E-27
376	4613	MYCN	26.9	12.6	-1.094182	2.97E-32
377	285590	SH3PXD2B	2.78	1.3	-1.096573	2.41E-14
378	11174	ADAMTS6	0.77	0.36	-1.096862	3.24E-04
379	2781	GNAZ	3.13	1.46	-1.100194	1.34E-05
380	81849	ST6GALNAC5	1.61	0.75	-1.102098	1.35E-06
381	81603	TRIM8	130.12	60.48	-1.105313	6.83E-216
382	11077	HSF2BP	1.12	0.52	-1.106915	0.027171255
383	84915	FAM222A	4.61	2.14	-1.107156	1.37E-10
384	10156	RASA4	4.01	1.86	-1.1083	8.37E-12
385	164045	HFM1	0.69	0.32	-1.108524	0.009750869
386	80237	ELL3	1.23	0.57	-1.109624	0.048211299
387	84249	PSD2	1.23	0.57	-1.109624	2.52E-04
388	5731	PTGER1	3.41	1.58	-1.109847	0.002026709
389	1043	CD52	45.82	21.22	-1.110553	4.54E-07
390	3215	HOXB5	2.77	1.28	-1.113742	0.001149389
391	26038	CHD5	6.48	2.99	-1.115848	4.59E-42
392	10202	DHRS2	25.99	11.96	-1.119739	1.51E-24
393	51761	ATP8A2	0.5	0.23	-1.120294	9.47E-04
394	90427	BMF	6.67	3.06	-1.124155	1.52E-20
395	50615	IL21R	17.07	7.83	-1.124379	6.28E-53
396	23098	SARM1	0.24	0.11	-1.125531	0.022437205
397	221468	TMEM217	8.67	3.97	-1.126893	1.09E-07
398	79844	ZDHHC11	2.6	1.19	-1.12755	6.21E-05
399	338761	C1QL4	1.64	0.75	-1.128733	0.007576382
400	6425	SFRP5	1.75	0.8	-1.129283	0.009184843
401	4987	OPRL1	0.79	0.36	-1.133856	0.016332199
402	8340	H2BC13	10.3	4.68	-1.138064	0.035239087
403	85508	SCRT2	6.78	3.08	-1.138355	2.14E-14
404	7852	CXCR4	11.38	5.16	-1.141058	8.47E-12
405	3566	IL4R	23.27	10.54	-1.142596	2.20E-50
406	3356	HTR2A	0.84	0.38	-1.14439	0.001568068
407	57103	TIGAR	0.31	0.14	-1.146841	0.011824855
408	1848	DUSP6	47.37	21.36	-1.149062	2.43E-113
409	340526	RTL5	1.22	0.55	-1.149378	2.86E-04
410	4059	BCAM	11.64	5.23	-1.154208	5.04E-22
411	57146	TMEM159	7.46	3.35	-1.155015	5.99E-08
412	57699	CPNE5	8.93	4.01	-1.155058	3.00E-13
413	342892	ZNF850	2.63	1.18	-1.156276	1.52E-14
414	5031	P2RY6	0.96	0.43	-1.158698	0.036934955
415	10335	IRAG1	2.91	1.3	-1.162508	4.23E-13
416	11145	PLAAT3	6.83	3.05	-1.163076	5.92E-05
417	89858	SIGLEC12	21.33	9.52	-1.16385	1.39E-31
418	1056	CEL	2.74	1.22	-1.167295	5.87E-05
419	353345	GPR141	1.55	0.69	-1.1676	1.60E-05
420	84867	PTPN5	2.25	1	-1.169925	2.40E-05
421	2811	GP1BA	103.25	45.84	-1.171463	1.64E-169
422	286527	TMSB15B	6.5	2.88	-1.174371	0.041021345
423	57060	PCBP4	11.22	4.97	-1.174755	5.23E-14
424	84620	ST6GAL2	2.33	1.03	-1.177686	3.33E-11

425	100528030	POC1B-GALNT4	1.38	0.61	-1.177787	1.20E-05
426	6565	SLC15A2	1.54	0.68	-1.179324	2.98E-04
427	11247	NXPH4	11.4	5.02	-1.183275	2.06E-11
428	84649	DGAT2	2.96	1.3	-1.187086	1.73E-05
429	55799	CACNA2D3	1.96	0.86	-1.188445	5.92E-06
430	6569	SLC34A1	9.87	4.33	-1.188683	1.48E-16
431	57484	RNF150	1.05	0.46	-1.190684	2.28E-08
432	51177	PLEKHO1	11.72	5.13	-1.191942	8.01E-17
433	57493	HEG1	2.13	0.93	-1.195551	3.69E-15
434	54753	ZNF853	0.62	0.27	-1.199309	0.017271956
435	140706	CCM2L	1.22	0.53	-1.202817	0.006645225
436	388813	LOC388813	3.71	1.61	-1.204358	1.08E-04
437	8809	IL18R1	3.35	1.45	-1.208108	2.05E-08
438	84826	SFT2D3	3.43	1.47	-1.222392	5.13E-10
439	26137	ZBTB20	0.28	0.12	-1.222392	6.38E-07
440	285513	GPRIN3	0.21	0.09	-1.222392	0.012615043
441	28232	SLCO3A1	35.85	15.33	-1.225615	7.63E-88
442	222950	NYAP1	1.03	0.44	-1.227069	0.001985773
443	333926	PPM1J	3.12	1.33	-1.23012	2.75E-04
444	51301	GCNT4	0.47	0.2	-1.232661	0.017275179
445	8778	SIGLEC5	3.06	1.3	-1.23502	1.08E-05
446	576	ADGRB2	2.59	1.1	-1.235449	1.02E-09
447	116	ADCYAP1	2.31	0.98	-1.237039	6.39E-06
448	57514	ARHGAP31	1.18	0.5	-1.238787	1.44E-06
449	109504726	ERV3-1-ZNF117	1.68	0.71	-1.24257	2.09E-11
450	960	CD44	55.06	23.22	-1.245637	4.61E-144
451	1687	GSDME	0.95	0.4	-1.247928	0.010607947
452	100130311	C17orf107	1.31	0.55	-1.252063	6.95E-04
453	4081	MAB21L1	5.41	2.27	-1.252936	3.47E-12
454	2920	CXCL2	7.64	3.2	-1.255501	5.47E-06
455	55450	CAMK2N1	9.24	3.87	-1.255559	4.82E-16
456	26974	ZNF285	0.55	0.23	-1.257798	0.002871268
457	84868	HAVCR2	0.79	0.33	-1.259387	0.04458711
458	3770	KCNJ14	0.6	0.25	-1.263034	0.014910666
459	27143	PALD1	0.36	0.15	-1.263034	0.04457981
460	339488	TFAP2E	2.24	0.93	-1.268196	3.44E-07
461	10991	SLC38A3	0.7	0.29	-1.271302	0.021698266
462	84958	SYTL1	5.75	2.38	-1.2726	7.23E-09
463	201181	ZNF385C	3.02	1.25	-1.27262	2.59E-07
464	492307	PPDPFL	16.13	6.67	-1.273988	3.73E-14
465	112476	PRRT2	8.7	3.59	-1.277032	1.34E-19
466	9436	NCR2	2.67	1.1	-1.279336	0.004274216
467	9098	USP6	0.73	0.3	-1.282934	2.36E-05
468	3730	ANOS1	0.56	0.23	-1.283793	0.001708647
469	8530	CST7	2.82	1.15	-1.294061	0.026175946
470	79861	TUBAL3	14.6	5.95	-1.295007	1.50E-19
471	4638	MYLK	17.89	7.29	-1.295163	8.56E-55
472	11033	ADAP1	6.21	2.53	-1.295456	4.67E-11
473	8516	ITGA8	1.99	0.81	-1.296775	5.60E-08
474	1571	CYP2E1	5.07	2.06	-1.299341	7.79E-07
475	7177	TPSAB1	16.64	6.76	-1.29956	2.44E-13
476	100527943	TGIF2-RAB5IF	1.97	0.8	-1.300124	0.021953361
477	51738	GHRL	1.11	0.45	-1.302563	0.031539051
478	3026	HABP2	0.99	0.4	-1.307429	0.004275153
479	9915	ARNT2	4.39	1.77	-1.310472	2.71E-24
480	57057	TBX20	1.39	0.56	-1.311586	0.003137238
481	51171	HSD17B14	4.3	1.73	-1.313565	0.001027245
482	10855	HPSE	6.62	2.65	-1.320839	1.24E-25
483	7730	ZNF177	1.2	0.48	-1.321928	0.003605709
484	27185	DISC1	4.02	1.6	-1.329124	1.92E-21
485	90273	CEACAM21	1.66	0.66	-1.330645	0.012696578

486	6866	TAC3	34.22	13.59	-1.332294	6.06E-17
487	375775	PNPLA7	0.53	0.21	-1.335603	0.006139095
488	9469	CHST3	0.48	0.19	-1.337035	0.00123536
489	57168	ASPHD2	0.76	0.3	-1.341037	0.005146116
490	2213	FCGR2B	2.94	1.16	-1.341691	5.48E-05
491	89822	KCNK17	2.46	0.97	-1.342602	0.001479997
492	220164	DOK6	0.23	0.09	-1.353637	0.021694331
493	8787	RGS9	34.47	13.48	-1.354521	1.23E-75
494	85407	NKD1	1.14	0.44	-1.373458	2.66E-18
495	2251	FGF6	3.45	1.33	-1.37517	6.22E-08
496	400745	SH2D5	0.96	0.37	-1.375509	5.83E-04
497	57156	TMEM63C	0.26	0.1	-1.378512	0.045366682
498	2620	GAS2	1.12	0.43	-1.38109	0.010610048
499	7474	WNT5A	4.04	1.55	-1.382087	1.86E-21
500	339541	ARMH1	2.98	1.14	-1.386279	1.91E-05
501	58512	DLGAP3	1.1	0.42	-1.389042	8.37E-05
502	4883	NPR3	3.51	1.34	-1.389238	1.89E-23
503	100170229	SRRM5	0.63	0.24	-1.392317	0.045359284
504	946	SIGLEC6	56.09	21.36	-1.392832	3.21E-150
505	639	PRDM1	0.92	0.35	-1.394279	8.27E-05
506	9619	ABCG1	15.32	5.82	-1.396325	5.19E-47
507	10220	GDF11	36.71	13.93	-1.397978	5.58E-230
508	101928589	LOC101928589	6.09	2.31	-1.398549	2.71E-10
509	5027	P2RX7	0.95	0.36	-1.399931	4.01E-05
510	973	CD79A	8.01	3.03	-1.402484	3.56E-07
511	4902	NRTN	2.65	1	-1.405992	0.00245246
512	56956	LHX9	1.38	0.52	-1.408085	1.56E-05
513	101928095	LOC101928095	0.69	0.26	-1.408085	0.025203105
514	1413	CRYBA4	11.86	4.45	-1.414227	5.21E-07
515	4978	OPCML	0.4	0.15	-1.415037	0.002994724
516	254173	TTLL10	0.94	0.35	-1.425306	0.01512831
517	3135	HLA-G	6.96	2.59	-1.426135	1.39E-04
518	100532731	COMMD3-BMI1	7.07	2.62	-1.432143	1.70E-22
519	85329	LGALS12	7.17	2.65	-1.435981	8.81E-11
520	5971	RELB	16.62	6.12	-1.441317	8.83E-33
521	23764	MAFF	13.34	4.91	-1.441964	1.05E-28
522	256126	SYCE2	2.56	0.94	-1.445411	0.008038519
523	5287	PIK3C2B	0.82	0.3	-1.450661	8.15E-06
524	102723360	LOC102723360	0.41	0.15	-1.450661	3.46E-06
525	8736	MYOM1	0.41	0.15	-1.450661	0.005017897
526	90993	CREB3L1	5.92	2.16	-1.454566	4.50E-15
527	6271	S100A1	31.24	11.31	-1.465796	3.98E-10
528	114784	CSMD2	2.57	0.93	-1.466466	1.05E-33
529	89790	SIGLEC10	2.05	0.74	-1.470027	1.89E-04
530	1513	CTSK	1.83	0.66	-1.471306	0.002451887
531	10085	EDIL3	1.11	0.4	-1.472488	7.58E-06
532	9498	SLC4A8	1.61	0.58	-1.472936	1.83E-07
533	54852	PAQR5	1.59	0.57	-1.479993	4.75E-04
534	5592	PRKG1	0.31	0.11	-1.494765	0.006960643
535	5791	PTPRE	1.16	0.41	-1.500429	0.002548446
536	170082	TCEANC	0.68	0.24	-1.5025	0.00591238
537	389840	MAP3K15	2.05	0.72	-1.509555	2.14E-10
538	57191	VN1R1	1.14	0.4	-1.510962	0.021346453
539	4050	LTB	9.52	3.34	-1.511113	4.41E-07
540	3655	ITGA6	2.71	0.95	-1.512293	1.05E-16
541	51555	PEX5L	0.2	0.07	-1.514573	0.005464337
542	4804	NGFR	1.35	0.47	-1.522227	2.52E-05
543	5792	PTPRF	9.07	3.15	-1.525751	3.78E-72
544	1464	CSPG4	0.75	0.26	-1.528379	1.70E-07
545	375190	FAM228B	1.1	0.38	-1.533432	0.044156171
546	57408	LRTM1	2.61	0.9	-1.536053	3.73E-04

547	602	BCL3	56.64	19.49	-1.539087	7.27E-102
548	53347	UBASH3A	14.97	5.15	-1.53943	2.71E-33
549	29108	PYCARD	3.5	1.2	-1.544321	0.012220466
550	389084	SNORC	1.4	0.48	-1.544321	1.71E-06
551	285180	RUFY4	0.38	0.13	-1.547488	0.009103217
552	2735	GLI1	0.47	0.16	-1.554589	0.021342576
553	83716	CRISPLD2	1.09	0.37	-1.558731	1.19E-07
554	440730	TRIM67	0.77	0.26	-1.566347	2.89E-07
555	5551	PRF1	14.89	5.01	-1.571461	1.29E-38
556	54039	PCBP3	1.17	0.39	-1.584963	0.002899138
557	2015	ADGRE1	0.51	0.17	-1.584963	0.012723276
558	169792	GLIS3	0.39	0.13	-1.584963	0.003563785
559	642799	NPIPA2	9.58	3.16	-1.600101	9.42E-29
560	83483	PLVAP	18.22	6	-1.602489	1.33E-43
561	91584	PLXNA4	4.11	1.35	-1.606179	4.15E-60
562	89927	BMERB1	17.03	5.58	-1.609741	1.45E-37
563	1240	CMKLR1	0.58	0.19	-1.610053	3.37E-04
564	54492	NEURL1B	1.8	0.58	-1.633872	1.12E-13
565	26212	OR2B6	2.4	0.77	-1.640104	0.00955482
566	5837	PYGM	0.53	0.17	-1.640458	0.031416298
567	57449	PLEKHG5	2.78	0.89	-1.643208	3.52E-14
568	25960	ADGRA2	2.8	0.89	-1.65355	6.53E-24
569	971	CD72	1.92	0.61	-1.654225	0.001032745
570	8913	CACNA1G	1.89	0.6	-1.655352	8.27E-19
571	85414	SLC45A3	43.08	13.57	-1.666598	1.10E-167
572	3553	IL1B	92.11	29	-1.667305	1.58E-142
573	140685	ZBTB46	3.31	1.04	-1.670248	7.09E-17
574	2268	FGR	1.41	0.44	-1.68012	5.49E-05
575	349667	RTN4RL2	11.89	3.71	-1.680258	1.13E-29
576	113791	PIK3IP1	2.13	0.66	-1.690316	1.71E-06
577	83937	RASSF4	0.94	0.29	-1.696608	3.29E-04
578	22998	LIMCH1	1.2	0.37	-1.697437	1.54E-09
579	79690	GAL3ST4	0.69	0.21	-1.716207	0.011100759
580	56833	SLAMF8	8.65	2.63	-1.717637	1.04E-27
581	29799	YPEL1	0.56	0.17	-1.719892	8.87E-04
582	285	ANGPT2	0.66	0.2	-1.722466	1.11E-04
583	534	ATP6V1G2	3.3	0.99	-1.736966	1.81E-05
584	57639	CCDC146	1.2	0.36	-1.736966	1.18E-05
585	56265	CPXM1	0.7	0.21	-1.736966	0.011098571
586	122402	TDRD9	0.3	0.09	-1.736966	0.022357752
587	192683	SCAMP5	12.54	3.73	-1.74929	3.59E-41
588	7128	TNFAIP3	21.02	6.22	-1.756776	2.32E-129
589	57636	ARHGAP23	0.88	0.26	-1.758992	5.31E-07
590	345222	MSANTD1	0.17	0.05	-1.765535	0.015261412
591	113451	AZIN2	2.56	0.75	-1.771181	2.90E-09
592	2998	GYS2	1.71	0.5	-1.773996	1.63E-06
593	2150	F2RL1	1.13	0.33	-1.775785	1.67E-04
594	9254	CACNA2D2	0.62	0.18	-1.784271	5.50E-05
595	121340	SP7	0.69	0.2	-1.786596	0.002382075
596	4916	NTRK3	0.73	0.21	-1.797507	2.22E-07
597	117286	CIB3	2.6	0.74	-1.812914	0.022361796
598	2740	GLP1R	0.88	0.25	-1.815575	1.20E-05
599	9715	FAM131B	1.42	0.4	-1.827819	5.69E-09
600	4688	NCF2	0.71	0.2	-1.827819	0.008164977
601	10125	RASGRP1	0.75	0.21	-1.836501	6.15E-06
602	3084	NRG1	0.69	0.19	-1.860597	0.001936178
603	7476	WNT7A	1.57	0.43	-1.868356	1.94E-04
604	1949	EFNB3	4.44	1.2	-1.887525	6.50E-20
605	1846	DUSP4	1.87	0.5	-1.903038	1.52E-15
606	57586	SYT13	0.3	0.08	-1.906891	0.005241618
607	115701	ALPK2	0.15	0.04	-1.906891	0.015854848

608	9381	OTOF	0.38	0.1	-1.925999	0.004145769
609	388419	BTBD17	0.84	0.22	-1.932886	0.009101387
610	2661	GDF9	0.73	0.19	-1.941897	0.010329311
611	595	CCND1	25.73	6.59	-1.965101	6.54E-160
612	1577	CYP3A5	0.43	0.11	-1.966833	8.17E-04
613	114118903	ARHGAP11A-SCG5	0.55	0.14	-1.974005	0.003326955
614	102800317	TPTEP2-CSNK1E	0.63	0.16	-1.97728	4.77E-04
615	126364	LRRC25	0.83	0.21	-1.982722	0.007213342
616	100133941	CD24	7.88	1.98	-1.992695	3.46E-25
617	53637	S1PR5	0.72	0.18	-2	0.003327706
618	113146	AHNAK2	0.04	0	-2	0.015264306
619	58480	RHOU	1.89	0.47	-2.007654	8.32E-12
620	84329	HVCN1	2.76	0.68	-2.021062	9.78E-07
621	149428	BNIPL	0.41	0.1	-2.035624	0.043958081
622	29933	GPR132	1.04	0.25	-2.056584	1.84E-05
623	51268	PIPOX	0.46	0.11	-2.06413	0.04395086
624	114335	CGB1	2.95	0.7	-2.075288	0.002282734
625	4624	MYH6	0.38	0.09	-2.078003	2.79E-04
626	80128	TRIM46	2.34	0.55	-2.089005	1.60E-10
627	7481	WNT11	7.41	1.71	-2.115477	4.47E-23
628	124460	SNX20	1.91	0.44	-2.117997	4.13E-09
629	5452	POU2F2	4.35	1	-2.121015	2.87E-33
630	5553	PRG2	109.75	25.05	-2.131338	3.87E-222
631	5159	PDGFRB	0.57	0.13	-2.13245	5.23E-06
632	58473	PLEKHB1	1.06	0.24	-2.142958	8.09E-04
633	10148	EBI3	2.57	0.58	-2.147644	2.46E-05
634	1436	CSF1R	1.64	0.37	-2.148099	4.91E-11
635	283	ANG	0.89	0.2	-2.153805	0.043943642
636	50839	TAS2R10	1.34	0.3	-2.159199	0.027491719
637	2953	GSTT2	2.55	0.57	-2.161463	6.63E-05
638	3055	HCK	6.81	1.48	-2.202058	6.06E-23
639	399474	TMEM200B	0.97	0.21	-2.207595	1.11E-04
640	2814	GP5	0.37	0.08	-2.209453	0.006086592
641	3067	HDC	45.12	9.67	-2.222179	1.35E-176
642	64137	ABCG4	0.19	0.04	-2.247928	0.047040297
643	3604	TNFRSF9	6.86	1.44	-2.25214	4.17E-56
644	64218	SEMA4A	3.53	0.73	-2.2737	9.79E-19
645	151056	PLB1	0.49	0.1	-2.292782	8.81E-06
646	773	CACNA1A	0.05	0	-2.321928	0.036250079
647	388284	C16orf86	0.67	0.13	-2.365649	0.022365841
648	3955	LFNG	3.38	0.65	-2.378512	4.12E-14
649	2824	GPM6B	0.21	0.04	-2.392317	0.010019666
650	22997	IGSF9B	0.43	0.08	-2.426265	2.94E-15
651	56126	PCDHB10	0.22	0.04	-2.459432	0.047055538
652	2012	EMP1	0.11	0.02	-2.459432	0.046451415
653	92949	ADAMTSL1	0.11	0.02	-2.459432	0.047047917
654	170261	ZCCHC12	0.94	0.17	-2.467126	1.81E-04
655	51314	NME8	0.72	0.13	-2.469485	8.17E-04
656	1441	CSF3R	0.39	0.07	-2.478047	0.010294535
657	8862	APLN	0.39	0.07	-2.478047	0.003709889
658	152273	FGD5	0.62	0.11	-2.494765	9.23E-08
659	441155	LOC441155	0.17	0.03	-2.5025	0.046458965
660	330	BIRC3	6.04	1.06	-2.510484	1.26E-86
661	23430	TPSD1	7.4	1.29	-2.520154	6.67E-21
662	64499	TPSB2	12.37	2.07	-2.579143	7.75E-25
663	7143	TNR	0.06	0.01	-2.584963	0.026858501
664	23671	TMEFF2	0.69	0.11	-2.649093	0.001375477
665	23604	DAPK2	1.91	0.3	-2.670538	3.89E-09
666	100049587	SIGLEC14	1.53	0.24	-2.672425	1.29E-08
667	378948	RBMY1B	0.51	0.08	-2.672425	0.017025677
668	259307	IL4I1	5.46	0.85	-2.683366	1.77E-18

669	196883	ADCY4	0.58	0.09	-2.688056	1.07E-04
670	79899	PRR5L	0.39	0.06	-2.70044	7.54E-04
671	10288	LILRB2	1.12	0.17	-2.719892	9.58E-09
672	241	ALOX5AP	2.01	0.3	-2.744161	0.001301982
673	79955	PDZD7	0.48	0.07	-2.777608	0.00221226
674	7483	WNT9A	0.14	0.02	-2.807355	0.046474074
675	5923	RASGRF1	0.07	0	-2.807355	0.036237803
676	729877	TBC1D3H	0.5	0.07	-2.836501	1.85E-05
677	221188	ADGRG5	2.97	0.41	-2.856767	4.48E-23
678	222487	ADGRG3	0.97	0.13	-2.899473	1.07E-05
679	5724	PTAFR	3.61	0.46	-2.972293	2.71E-34
680	313	AOAH	0.24	0.03	-3	0.015255627
681	8748	ADAM20	0.08	0	-3	0.019233603
682	3689	ITGB2	3.5	0.43	-3.024946	8.20E-24
683	286436	H2BW2	0.33	0.04	-3.044394	0.046466518
684	102724265	LOC102724265	0.25	0.03	-3.058894	0.046436321
685	124602	KIF19	0.52	0.06	-3.115477	4.63E-05
686	6547	SLC8A3	0.53	0.06	-3.142958	6.84E-08
687	3887	KRT81	0.36	0.04	-3.169925	0.026849026
688	56924	PAK6	0.36	0.04	-3.169925	1.98E-04
689	56659	KCNK13	0.27	0.03	-3.169925	0.046481632
690	497189	TIFAB	0.18	0.02	-3.169925	0.0033927
691	51705	EMCN	0.18	0.02	-3.169925	0.015258519
692	56122	PCDHB14	0.18	0.02	-3.169925	0.026853763
693	4625	MYH7	0.09	0.01	-3.169925	0.046443867
694	54847	SIDT1	0.19	0.02	-3.247928	0.015267202
695	10090	UST	0.1	0	-3.321928	0.036219405
696	26034	IPCEF1	0.1	0	-3.321928	0.002788615
697	653489	RGPD3	1.72	0.17	-3.338802	2.88E-27
698	126147	NTN5	0.41	0.04	-3.357552	0.008554271
699	51725	FBXO40	0.11	0.01	-3.459432	0.026863242
700	81031	SLC2A10	0.34	0.03	-3.5025	7.54E-04
701	56127	PCDHB9	0.24	0.02	-3.584963	8.03E-04
702	4049	LTA	3.51	0.28	-3.647972	5.47E-11
703	924	CD7	4.12	0.32	-3.686501	2.56E-13
704	5940	RBMY1A1	1.77	0.13	-3.767166	3.78E-08
705	149345	SHISA4	1.5	0.11	-3.769387	5.52E-06
706	219537	SMTNL1	0.83	0.06	-3.790077	6.29E-04
707	157695	TDRP	0.15	0	-3.906891	0.019230062
708	26154	ABCA12	0.15	0.01	-3.906891	1.98E-04
709	79630	C1orf54	4.43	0.27	-4.036275	0.002662782
710	100529144	CORO7-PAM16	0.17	0	-4.087463	0.019237146
711	100529215	ZNF559-ZNF177	0.18	0	-4.169925	0.036225536
712	100423062	IGLL5	3.78	0.19	-4.314315	1.22E-13
713	2214	FCGR3A	0.21	0	-4.392317	0.03624394
714	100506164	HSFX1	0.28	0	-4.807355	0.019226522
715	440804	RIMBP3B	0.28	0.01	-4.807355	1.88E-06
716	3855	KRT7	0.3	0	-4.906891	0.036213276
717	102723737	CT45A8	0.98	0.03	-5.029747	0.005316333
718	27122	DKK3	0.33	0	-5.044394	7.56E-04
719	140691	TRIM69	0.37	0	-5.209453	0.010136111
720	553158	PRR5-ARHGAP8	0.43	0	-5.426265	7.56E-04
721	360200	TMPRSS9	0.45	0	-5.491853	2.74E-05
722	27232	GNMT	0.55	0	-5.78136	0.036231668
723	388372	CCL4L1	1.1	0	-6.78136	0.005318607
724	100526761	CCDC169-SOHLH2	1.36	0	-7.087463	3.29E-14
