# Supplementary material for: FLI1 induces erythroleukemia through opposing effects on UBASH3A and UBASH3B expression
Source: BMC Cancer. 2024 Mar 9;24:326. doi: 10.1186/s12885-024-12075-2 (PMC10925000; doi:10.1186/s12885-024-12075-2)
Supplement: Supplementary file 2 — Supplementary Materials 2. [file 12885_2024_12075_MOESM2_ESM.pdf]

Supplemental Table 2. Differential expression of genes in Scrambled and shUBASH3B

	Gene ID	Gene Symbol	Scrambled FPKM	shUBASH3B FPKM	log2FoldChange	Padj
1	58530	LY6G6D	0	6.38	9.3174126	5.38E-04
2	100529063	BCL2L2-PABPN1	0	6.29	9.2969162	1.88E-50
3	8363	H4C11	0	4.3	8.7481929	0.011871465
4	552891	DNAJC25-GNG10	0	1.16	6.857981	1.86E-06
5	9506	PAGE4	0	1.12	6.8073549	0.006434251
6	728689	EIF3CL	0	1.01	6.6582115	7.69E-13
7	100526832	PHOSPHO2-KLHL23	0	0.95	6.5698556	9.36E-17
8	102723680	CT45A9	0	0.48	5.5849625	0.021715748
9	725	C4BPB	0	0.46	5.523562	0.01187313
10	49	ACR	0	0.44	5.4594316	0.0118698
11	100528032	KLRC4-KLRK1	0	0.4	5.3219281	0.003477916
12	2353	FOS	2.53	86.76	5.0998208	0
13	105378260	LOC105378260	0	0.34	5.0874628	0.039601352
14	112268186	LOC112268186	0	0.33	5.0443941	0.03959149
15	140711	TLDC2	0	0.32	5	0.021701384
16	2826	CCR10	0	0.31	4.9541963	0.02171
17	3231	HOXD1	0	0.27	4.7548875	0.021721499
18	221749	PXDC1	0	0.25	4.6438562	0.021704255
19	5309	PITX3	0.06	1.48	4.6244909	1.02E-06
20	146212	KCTD19	0	0.24	4.5849625	0.003477379
21	90853	SPOCD1	0	0.23	4.523562	0.011874796
22	3200	HOXA3	0.02	0.46	4.523562	6.72E-05
23	729238	SFTPA2	0	0.2	4.3219281	0.039616155
24	353376	TICAM2	0	0.2	4.3219281	0.006436148
25	3196	TLX2	0	0.2	4.3219281	0.03959642
26	10083	USH1C	0	0.2	4.3219281	0.021724376
27	8875	VNN2	0.2	3.88	4.2779847	5.03E-23
28	106821730	BUB1B-PAK6	0	0.19	4.2479275	0.003478453
29	9071	CLDN10	0	0.19	4.2479275	0.039586561
30	10110	SGK2	0.04	0.75	4.2288187	3.66E-05
31	1943	EFNA2	0	0.18	4.169925	0.039635908
32	2272	FHIT	0	0.17	4.0874628	0.003476306
33	5156	PDGFRA	0	0.17	4.0874628	0.003476842
34	50509	COL5A3	0	0.16	4	5.38E-04
35	84466	MEGF10	0.01	0.14	3.8073549	6.75E-04
36	89866	SEC16B	0	0.14	3.8073549	0.011876463
37	143503	OR51E1	0	0.14	3.8073549	0.039626029
38	2886	GRB7	0.04	0.55	3.7813597	3.79E-04
39	2354	FOSB	1.29	17.24	3.7403168	1.25E-187
40	401563	C9orf139	0	0.13	3.7004397	0.021718623
41	57509	MTUS1	0	0.13	3.7004397	0.021712874
42	1959	EGR2	0.15	1.84	3.6166714	3.24E-15
43	79784	MYH14	0.01	0.12	3.5849625	0.006440135
44	80176	SPSB1	0.02	0.24	3.5849625	0.011164306
45	91608	RASL10B	0.02	0.24	3.5849625	0.011159583
46	10365	KLF2	0.03	0.35	3.5443205	0.002112542
47	139728	PNCK	0.04	0.45	3.4918531	0.011162731
48	5790	PTPRCAP	0.29	3.22	3.4729359	3.97E-07
49	2018	EMX2	0.12	1.33	3.4703199	8.29E-10
50	151790	WDR49	0	0.11	3.4594316	0.03964085
51	11067	DEPP1	0.36	3.93	3.4484605	1.43E-21
52	1958	ERG1	28.75	311.89	3.4394034	0
53	55287	TMEM40	0.14	1.51	3.4310498	2.12E-08
54	4160	MC4R	0.05	0.53	3.4059924	0.006442032
55	5166	PDK4	0.02	0.21	3.3923174	0.011161157
56	114132	SIGLEC11	0.21	2.17	3.3692338	1.12E-16
57	163732	CITED4	0.06	0.61	3.3457748	0.019111715
58	374786	EFCAB5	0	0.1	3.3219281	0.039611219

59	3777	KCNK3	0	0.1	3.3219281	0.006437096
60	100526783	ARPIN-AP3S2	0	0.1	3.3219281	0.006435199
61	130813	C2orf50	0	0.1	3.3219281	0.021707128
62	3491	CCN1	1.74	17.39	3.3210987	2.16E-98
63	1572	CYP2F1	0.09	0.88	3.2895066	1.17E-04
64	440503	PLIN5	0.03	0.29	3.2730185	0.019106589
65	59277	NTN4	0.64	6.18	3.271463	9.99E-58
66	9080	CLDN9	0.05	0.48	3.2630344	0.019109151
67	359	AQP2	0.02	0.19	3.2479275	0.006442982
68	146862	UNC45B	0.02	0.19	3.2479275	6.75E-04
69	11173	ADAMTS7	0.22	2.07	3.2340553	6.47E-31
70	123591	TMEM266	0.03	0.27	3.169925	0.032433861
71	133	ADM	0.11	0.98	3.1552782	6.05E-04
72	1277	COL1A1	0.31	2.73	3.1385608	1.82E-42
73	2669	GEM	0.04	0.34	3.0874628	0.019104027
74	3725	JUN	8.83	72.9	3.0454335	0
75	114794	ELFN2	0.02	0.16	3	7.95E-04
76	1278	COL1A2	0	0.08	3	0.039621091
77	57348	TTYH1	0.04	0.32	3	0.032442113
78	5743	PTGS2	1.14	9.08	2.9936585	1.70E-98
79	83715	ESPN	0.42	3.26	2.9564107	1.49E-25
80	199720	GGN	0.07	0.54	2.9475326	0.001771221
81	3399	ID3	1.78	13.64	2.9378945	1.53E-23
82	51754	TMEM8B	0.5	3.82	2.9335726	2.28E-21
83	148738	HJV	0.06	0.45	2.9068906	0.006441084
84	139221	PWWP3B	0.02	0.15	2.9068906	0.032437987
85	154091	SLC2A12	0.03	0.22	2.8744691	0.013831995
86	11155	LDB3	0.03	0.22	2.8744691	0.036637146
87	187	APLNR	1.69	12.27	2.8600401	8.37E-108
88	57644	MYH7B	0.31	2.22	2.8402196	2.98E-33
89	57159	TRIM54	0.08	0.57	2.83289	0.005026485
90	158038	LINGO2	0.01	0.07	2.8073549	0.023775469
91	92689	FAM114A1	0.81	5.64	2.7997014	7.49E-47
92	143098	MPP7	0.17	1.18	2.7951802	3.35E-06
93	121793	TEX29	0.14	0.96	2.7776076	0.036641751
94	127343	DMBX1	0.05	0.34	2.7655347	0.001320892
95	56666	PANX2	1.26	8.47	2.7489382	3.99E-55
96	2348	FOLR1	0.41	2.7	2.7192636	7.66E-05
97	107985381	LOC107985381	0.14	0.91	2.7004397	0.008368768
98	6383	SDC2	0.32	2.07	2.693487	1.79E-15
99	148398	SAMD11	0.07	0.45	2.6844982	0.002990439
100	55281	TMEM140	0.24	1.52	2.662965	1.21E-06
101	10123	ARL4C	0.06	0.38	2.662965	4.75E-04
102	55586	MIOX	0.05	0.31	2.6322682	0.021698514
103	23779	ARHGAP8	1.33	8.21	2.625956	2.51E-25
104	100113407	TMEM170B	0.02	0.12	2.5849625	0.005841081
105	6492	SIM1	0	0.06	2.5849625	0.039606285
106	116444	GRIN3B	0.1	0.6	2.5849625	9.36E-04
107	10579	TACC2	0.65	3.86	2.5700892	6.00E-43
108	112268237	LOC112268237	0.19	1.12	2.5594274	0.002186675
109	56704	JPH1	0.07	0.41	2.5501971	3.49E-04
110	2069	EREG	1.34	7.83	2.5467793	3.04E-73
111	163175	LGI4	0.06	0.35	2.5443205	0.013833906
112	51481	VCX3A	0.19	1.08	2.50696	0.022613119
113	126006	PCP2	2.09	11.8	2.497212	4.34E-13
114	100128569	C19orf71	0.34	1.91	2.489966	0.022610139
115	29948	OSGIN1	0.89	4.97	2.4813686	1.16E-17
116	197257	LDHD	0.46	2.53	2.4594316	4.79E-10
117	266727	MDGA1	0.1	0.54	2.4329594	3.22E-05
118	51296	SLC15A3	0.09	0.48	2.4150375	0.005840212
119	1364	CLDN4	0.13	0.69	2.4080847	0.005839343

120	3726	JUNB	88.13	467.09	2.4059954	0
121	2555	GABRA2	1.34	7.06	2.3974352	5.82E-86
122	220004	PPP1R32	0.81	4.26	2.3948596	1.52E-11
123	29119	CTNNA3	0.39	2.05	2.3940779	1.48E-12
124	554313	H4C15	2.81	14.58	2.3753487	1.51E-04
125	8370	H4C14	2.81	14.58	2.3753487	1.51E-04
126	347735	SERINC2	0.12	0.62	2.3692338	0.009398291
127	57662	CAMSAP3	0.26	1.34	2.3656495	6.54E-11
128	100293534	C4B_2	0.15	0.76	2.3410369	2.07E-08
129	6615	SNAI1	1.22	6.17	2.3383893	8.14E-18
130	136853	SSC4D	0.48	2.42	2.3339007	3.32E-12
131	51513	ETV7	0.34	1.71	2.3303897	1.50E-05
132	646951	MINDY4B	0.49	2.45	2.3219281	3.88E-09
133	79190	IRX6	0.08	0.4	2.3219281	0.00939695
134	166336	PRICKLE2	0.08	0.4	2.3219281	2.88E-07
135	51308	REEP2	0.18	0.88	2.2895066	6.41E-04
136	65078	RTN4R	0.24	1.17	2.2854022	1.69E-04
137	159686	CFAP58	0.07	0.34	2.2801079	0.009395609
138	9185	REPS2	0.07	0.34	2.2801079	2.46E-04
139	11189	CELF3	0.06	0.29	2.2730185	6.81E-04
140	652968	CASTOR1	0.65	3.14	2.2722529	6.04E-08
141	6640	SNTA1	1	4.8	2.2630344	4.49E-18
142	6521	SLC4A1	0.25	1.2	2.2630344	7.78E-08
143	333926	PPM1J	3.12	14.93	2.2585962	1.73E-40
144	114108587	ATF7-NPFF	0.49	2.33	2.2494763	5.27E-07
145	252884	ZNF396	0.11	0.52	2.2410081	0.022403966
146	100529215	ZNF559-ZNF177	0.18	0.85	2.2394659	1.69E-04
147	28999	KLF15	0.12	0.56	2.2223924	0.003899359
148	358	AQP1	23.84	110.9	2.2178032	0
149	57477	SHROOM4	0.08	0.37	2.2094534	4.32E-04
150	402778	IFITM10	0.1	0.46	2.2016339	0.00102651
151	54932	EXD3	1.03	4.71	2.1930827	1.10E-08
152	100532736	MICOS10-NBL1	0.11	0.5	2.1844246	0.015011171
153	2868	GRK4	0.73	3.29	2.1721192	8.60E-10
154	4585	MUC4	0.04	0.18	2.169925	1.11E-07
155	114792	KLHL32	0.45	2.01	2.1591986	6.94E-07
156	256949	KANK3	0.32	1.42	2.1497471	2.46E-07
157	107984638	LOC107984638	0.69	3.06	2.1488634	9.16E-07
158	7450	VWF	0.56	2.48	2.1468414	1.98E-37
159	26548	ITGB1BP2	0.27	1.19	2.1399303	0.006219393
160	65989	DLK2	0.15	0.66	2.1375035	0.035882325
161	4082	MARCKS	0.33	1.44	2.1255309	1.04E-10
162	222183	SRRM3	0.28	1.22	2.1233824	4.44E-08
163	3134	HLA-F	17.24	74.76	2.1165068	1.65E-116
164	8532	CPZ	0.66	2.86	2.1154772	6.59E-10
165	94240	EPSTI1	2.92	12.52	2.1001943	9.09E-26
166	7057	THBS1	1.42	6.08	2.0981804	3.00E-58
167	162461	TMEM92	0.42	1.79	2.0914984	0.003482978
168	102724473	GAGE10	0.69	2.9	2.0713846	0.037196509
169	102724219	LOC102724219	0.21	0.88	2.0671142	9.14E-06
170	374	AREG	1.6	6.7	2.0660892	1.96E-11
171	107985416	LOC107985416	0.44	1.84	2.0641303	0.009775612
172	151126	ZNF385B	0.75	3.13	2.0612002	1.11E-16
173	57333	RCN3	12.79	53.12	2.0542389	2.25E-106
174	160518	DENND5B	1.05	4.36	2.0539388	1.88E-16
175	440689	H2BC18	1.52	6.3	2.0512805	0.006294569
176	64285	RHBDF1	0.29	1.2	2.0489096	5.67E-06
177	5730	PTGDS	0.37	1.53	2.0479345	0.037205838
178	22924	MAPRE3	1.88	7.77	2.0471819	5.33E-21
179	54541	DDIT4	43.29	178.84	2.0465637	0
180	79605	PGBD5	0.21	0.86	2.0339473	1.51E-15

181	128506	OCSTAMP	0.21	0.85	2.0170735	0.001635806
182	2815	GP9	135.14	546.55	2.0158987	0
183	23678	SGK3	0.27	1.09	2.0132968	7.38E-08
184	4035	LRP1	0.61	2.46	2.0117772	3.03E-58
185	6649	SOD3	0.18	0.72	2	0.037187184
186	5155	PDGFB	0.52	2.08	2	2.68E-13
187	8609	KLF7	0.06	0.24	2	0.009697476
188	8681	JMJD7-PLA2G4B	0.84	3.33	1.9870609	3.22E-17
189	8357	H3C10	7.14	28.26	1.9847655	4.40E-10
190	100132074	FOXO6	0.64	2.53	1.9829936	3.25E-11
191	7538	ZFP36	37.83	149.5	1.9825428	0
192	9592	IER2	85.47	335.83	1.9742411	0
193	2	A2M	0.28	1.1	1.9740048	2.11E-08
194	4332	MNDA	0.19	0.74	1.9615259	0.015208319
195	55366	LGR4	3.27	12.73	1.9608699	1.85E-98
196	51655	RASD1	0.82	3.17	1.950787	5.07E-08
197	8970	H2BC11	8.94	34.56	1.9507565	1.40E-11
198	2672	GFI1	0.81	3.13	1.9501688	7.34E-22
199	221914	GPC2	1.06	4.05	1.9338576	9.35E-15
200	55138	FAM90A1	0.22	0.84	1.9328858	0.001072619
201	388588	SMIM1	46.16	175.69	1.9283169	2.23E-66
202	100129924	TMEM269	0.15	0.57	1.9259994	0.001675538
203	125965	COX6B2	0.22	0.83	1.9156078	0.044989928
204	150483	TEKT4	1.45	5.47	1.9154879	1.36E-11
205	3664	IRF6	0.25	0.93	1.8953026	6.77E-07
206	6899	TBX1	0.77	2.86	1.8930848	7.37E-08
207	122622	ADSS1	0.91	3.38	1.8930848	3.30E-08
208	7022	TFAP2C	0.1	0.37	1.8875253	0.035891366
209	553158	PRR5-ARHGAP8	0.43	1.59	1.8866182	3.29E-05
210	23542	MAPK8IP2	0.06	0.22	1.8744691	0.014800904
211	10391	CORO2B	0.09	0.33	1.8744691	0.023497379
212	376940	ZC3H6	0.41	1.5	1.8712667	1.09E-15
213	106865373	GET1-SH3BGR	0.29	1.06	1.8699395	0.015210398
214	283849	EXOC3L1	0.37	1.35	1.8673622	5.03E-05
215	22801	ITGA11	0.74	2.67	1.8512426	3.58E-36
216	645426	TMEM191C	0.28	1.01	1.8508566	0.023500466
217	130733	TMEM178A	0.73	2.59	1.8269837	1.36E-05
218	3303	HSPA1A	12.43	43.99	1.8233493	7.74E-132
219	5997	RGS2	2.99	10.57	1.821758	1.21E-16
220	285704	RGMB	0.76	2.68	1.8181617	2.91E-16
221	51266	CLEC1B	37.19	130.17	1.8074103	9.56E-105
222	102724965	LOC102724965	0.1	0.35	1.8073549	0.037201173
223	1950	EGF	2.06	7.2	1.8053526	2.22E-48
224	79148	MMP28	0.53	1.85	1.803461	0.009328118
225	1844	DUSP2	2.79	9.72	1.8006912	2.20E-20
226	201299	RDM1	3.18	11.04	1.7956415	6.12E-15
227	27134	TJP3	0.17	0.59	1.7951802	0.003880854
228	155382	VPS37D	0.8	2.77	1.7918141	5.57E-06
229	100129792	CCDC152	0.09	0.31	1.7842713	0.035886845
230	54760	PCSK4	0.85	2.92	1.7804336	4.01E-10
231	388284	C16orf86	0.67	2.3	1.7794009	0.003094907
232	10045	SH2D3A	0.51	1.75	1.7787858	3.22E-05
233	122416	ANKRD9	3.11	10.59	1.7677161	1.87E-73
234	3669	ISG20	0.42	1.43	1.7675539	4.99E-04
235	2104	ESRRG	0.05	0.17	1.7655347	0.023778589
236	199223	TTC21A	0.1	0.34	1.7655347	0.014024024
237	388403	YPEL2	1.03	3.5	1.7647106	4.93E-24
238	105375683	LOC105375683	2.55	8.65	1.7622029	4.44E-11
239	340390	WDR97	0.08	0.27	1.7548875	0.019191487
240	7164	TPD52L1	1.54	5.17	1.7472339	8.57E-05
241	5649	RELN	0.03	0.1	1.7369656	0.02240101

242	110354863	ZNF660-ZNF197	0.54	1.8	1.7369656	9.66E-19
243	1960	EGR3	0.06	0.2	1.7369656	0.037191846
244	729359	PLIN4	0.06	0.2	1.7369656	0.014025957
245	94241	TP53INP1	1.73	5.76	1.7352968	6.84E-41
246	10347	ABCA7	8.3	27.63	1.7350523	3.08E-203
247	100529144	CORO7-PAM16	0.17	0.56	1.7198921	0.003881446
248	55501	CHST12	6.05	19.89	1.7170362	4.60E-34
249	100310846	ANKRD61	0.35	1.15	1.716207	0.014022091
250	729438	CASTOR2	3.54	11.63	1.7160298	6.61E-28
251	8792	TNFRSF11A	0.11	0.36	1.7104934	0.0074848
252	4647	MYO7A	0.11	0.36	1.7104934	4.13E-04
253	5348	FXYD1	1.54	5.03	1.707628	0.008785321
254	2625	GATA3	0.35	1.14	1.703607	1.11E-04
255	3304	HSPA1B	31.6	102.73	1.7008611	4.02E-294
256	29993	PACSIN1	0.08	0.26	1.7004397	0.033586447
257	100462983	MTRNR2L3	0.55	1.78	1.6943737	0.014020159
258	57731	SPTBN4	0.86	2.78	1.6926763	2.85E-14
259	9871	SEC24D	11.77	37.99	1.6905054	5.95E-180
260	793	CALB1	0.45	1.45	1.688056	4.47E-05
261	604	BCL6	0.27	0.87	1.688056	1.69E-05
262	388327	C17orf100	0.6	1.93	1.6855664	2.48E-04
263	143686	SESN3	1.91	6.13	1.6823144	1.82E-72
264	664	BNIP3	14.98	48.02	1.6805978	4.39E-82
265	3084	NRG1	0.69	2.21	1.6793781	7.16E-04
266	84159	ARID5B	0.3	0.96	1.6780719	2.66E-10
267	57577	CCDC191	0.15	0.48	1.6780719	6.81E-04
268	6035	RNASE1	7.28	23.25	1.6752204	1.07E-19
269	3050	HBZ	101.47	322.15	1.6666793	5.05E-207
270	8337	H2AC18	45.83	145.42	1.6658615	3.68E-46
271	768211	RELL1	0.77	2.44	1.6639508	0.012724986
272	2255	FGF10	0.12	0.38	1.662965	0.00369253
273	92840	REEP6	48.95	154.75	1.6605586	8.34E-207
274	84722	PSRC1	11.24	35.52	1.6599895	8.36E-66
275	225	ABCD2	0.13	0.41	1.6571123	2.76E-04
276	27146	FAM184B	0.07	0.22	1.6520767	0.009326786
277	55150	C19orf73	0.86	2.7	1.6505508	0.020900884
278	50865	HEBP1	15.5	48.63	1.6495784	7.64E-53
279	3135	HLA-G	6.96	21.82	1.6484919	2.12E-15
280	23659	PLA2G15	3.88	12.13	1.644451	1.45E-35
281	7923	HSD17B8	1.9	5.93	1.6420327	6.77E-06
282	79990	PLEKHH3	5.77	18	1.6413537	1.31E-62
283	25822	DNAJB5	1.28	3.99	1.6402449	5.77E-12
284	2335	FN1	0.61	1.9	1.6391183	4.81E-18
285	54478	PIMREG	0.53	1.65	1.6384018	5.40E-04
286	144717	PHETA1	4.41	13.68	1.6332177	2.33E-37
287	150921	TCF23	0.1	0.31	1.6322682	0.013100946
288	221002	RASGEF1A	1.06	3.28	1.6296316	1.83E-06
289	718	C3	0.87	2.69	1.6285189	6.05E-17
290	120071	LARGE2	0.49	1.51	1.6236949	8.56E-05
291	1515	CTSV	0.74	2.28	1.6234366	5.10E-12
292	3162	HMOX1	1.36	4.19	1.6233436	2.98E-07
293	10509	SEMA4B	14.12	43.38	1.61929	5.45E-183
294	55357	TBC1D2	0.56	1.72	1.6189098	3.16E-06
295	8654	PDE5A	2.21	6.77	1.6151095	4.19E-49
296	339456	TMEM52	1.31	4.01	1.6140354	5.40E-04
297	26207	PITPNC1	3.1	9.48	1.6126188	6.35E-69
298	8646	CHRD	0.18	0.55	1.6114347	0.003693096
299	246721	POLR2J2	1.76	5.35	1.6039635	1.10E-11
300	8343	H2BC7	3.96	12.02	1.6018646	0.001263346
301	9026	HIP1R	6.32	19.17	1.6008539	7.05E-87
302	5794	PTPRH	1.93	5.84	1.5973675	1.00E-24

303	23493	HEY2	3.51	10.62	1.5972408	1.48E-29
304	282974	STK32C	6.32	19.11	1.5963313	1.34E-08
305	56139	PCDHA10	0	0.03	1.5849625	0.039630968
306	9249	DHRS3	10.96	32.88	1.5849625	9.88E-43
307	721	C4B	0.17	0.51	1.5849625	3.92E-04
308	374977	MROH7	0.12	0.36	1.5849625	0.01309912
309	1382	CRABP2	0.48	1.44	1.5849625	0.049676958
310	387763	C11orf96	10.37	31.09	1.5840347	3.70E-35
311	79873	NUDT18	1.62	4.84	1.5790132	1.04E-07
312	100128071	FAM229A	2.35	7	1.5746942	3.15E-04
313	3800	KIF5C	0.74	2.2	1.5719063	2.82E-15
314	84992	PIGY	17.83	52.87	1.5681426	4.29E-65
315	57561	ARRDC3	2.13	6.31	1.5667866	4.64E-29
316	84960	CCDC183	0.37	1.09	1.558731	0.012003445
317	4294	MAP3K10	2.19	6.44	1.5561298	7.47E-19
318	148534	TLCD4	0.98	2.88	1.5552152	4.53E-22
319	8153	RND2	0.16	0.47	1.5545889	0.005122062
320	9024	BRSK2	1.03	3.02	1.5519042	4.10E-14
321	57716	PRX	0.8	2.34	1.5484366	6.71E-12
322	8630	HSD17B6	0.63	1.84	1.546282	0.003124479
323	80235	PIGZ	0.21	0.61	1.5384199	0.006238667
324	55321	TMEM74B	1.51	4.38	1.5363823	1.18E-07
325	8577	TMEFF1	1.45	4.2	1.5343364	1.21E-11
326	79094	CHAC1	26.98	78.05	1.5325083	2.92E-108
327	8349	H2BC21	6.94	20.04	1.5298749	1.78E-43
328	467	ATF3	5.31	15.28	1.5248608	3.35E-29
329	8676	STX11	3.08	8.86	1.5243763	2.85E-38
330	117247	SLC16A10	1.92	5.52	1.523562	8.68E-07
331	84144	SYDE2	3.62	10.39	1.5211341	7.50E-53
332	113230	MISP3	2.57	7.37	1.5198963	8.53E-11
333	101928120	LOC101928120	0.45	1.29	1.5193742	0.022398055
334	2318	FLNC	2.45	7.01	1.5166327	8.80E-68
335	2264	FGFR4	3.39	9.67	1.5122306	7.77E-30
336	28227	PPP2R3B	4.53	12.9	1.5097881	1.06E-30
337	105372704	LOC105372704	0.57	1.62	1.50696	0.002687876
338	114822	RHPN1	4.07	11.56	1.5060407	1.68E-43
339	81027	TUBB1	3.78	10.72	1.5038468	2.00E-35
340	84073	MYCBPAP	0.53	1.5	1.5008982	1.01E-04
341	642658	SCX	0.88	2.49	1.5005703	0.002579604
342	2802	GOLGA3	6.49	18.36	1.5002757	5.03E-96
343	800	CALD1	4.27	12.06	1.4979219	1.04E-55
344	1821	DRP2	0.17	0.48	1.4974997	1.82E-04
345	3006	H1-2	228.3	644.52	1.4972943	0
346	6261	RYR1	0.5	1.41	1.4956952	1.89E-23
347	10297	APC2	0.11	0.31	1.4947647	6.04E-04
348	440087	SMCO3	0.11	0.31	1.4947647	0.023494293
349	28986	MAGEH1	7.01	19.74	1.4936356	5.46E-25
350	148641	SLC35F3	0.21	0.59	1.4903256	0.010908293
351	79741	CCDC7	0.26	0.73	1.4893848	0.019926555
352	150051	LOC150051	3.94	11.06	1.4890839	2.46E-07
353	10811	NOXA1	0.31	0.87	1.4887472	0.044995464
354	760	CA2	32.29	90.62	1.4887421	2.25E-122
355	726	CAPN5	2.72	7.62	1.4861843	1.99E-33
356	254013	ETFBKMT	0.1	0.28	1.4854268	0.015662699
357	8728	ADAM19	0.89	2.49	1.4842685	2.49E-17
358	212	ALAS2	9.78	27.34	1.4831069	6.97E-47
359	6909	TBX2	7.7	21.52	1.4827477	1.51E-71
360	2633	GBP1	1.07	2.99	1.4825347	3.04E-09
361	388228	SBK1	0.33	0.92	1.4791678	1.67E-05
362	140706	CCM2L	1.22	3.4	1.4786536	3.67E-09
363	51129	ANGPTL4	0.59	1.64	1.474909	0.001529599

364	3005	H1-0	668.07	1854.01	1.4725778	0
365	130271	PLEKHH2	1.33	3.69	1.4721946	8.47E-25
366	8341	H2BC15	5.45	15.12	1.47213	5.46E-05
367	8339	H2BC8	8.27	22.89	1.4687582	7.89E-06
368	10953	TOMM34	10.22	28.24	1.4663449	8.76E-50
369	55638	SYBU	0.29	0.8	1.4639471	0.00700816
370	7349	UCN	2.95	8.13	1.4625404	3.45E-05
371	4207	BORCS8-MEF2B	1.41	3.88	1.4603615	8.71E-06
372	23492	CBX7	1.07	2.94	1.4582054	2.94E-12
373	4137	MAPT	0.82	2.25	1.4562292	2.31E-13
374	2801	GOLGA2	13.04	35.67	1.4517673	1.25E-143
375	10699	CORIN	0.26	0.71	1.4493074	0.031118977
376	645	BLVRB	164.13	447.25	1.4462425	1.64E-233
377	5641	LGMN	2.83	7.71	1.4459288	1.53E-13
378	8924	HERC2	33.25	90.56	1.4455196	0
379	3299	HSF4	0.93	2.53	1.4438348	2.62E-07
380	144100	PLEKHA7	10.51	28.59	1.443748	1.06E-147
381	8635	RNASET2	13.31	36.18	1.4426818	1.24E-33
382	124790	HEXIM2	3.33	9.05	1.4423956	3.47E-08
383	3339	HSPG2	17.51	47.56	1.4415696	0
384	441150	C6orf226	7.75	21.04	1.4408665	1.04E-06
385	1768	DNAH6	0.07	0.19	1.4405726	0.005556477
386	8853	ASAP2	1.29	3.5	1.4399839	8.25E-19
387	57801	HES4	3.73	10.1	1.4371078	1.18E-08
388	126147	NTN5	0.41	1.11	1.4368639	0.012572614
389	1613	DAPK3	7.49	20.27	1.4363085	2.52E-39
390	267	AMFR	27.6	74.69	1.4362468	2.45E-264
391	2064	ERBB2	1.39	3.76	1.4356478	6.23E-18
392	22927	HABP4	3.15	8.51	1.4338073	2.47E-21
393	27175	TUBG2	2.34	6.32	1.433416	1.16E-08
394	135154	SDHAF4	3.24	8.75	1.4332892	9.81E-09
395	2308	FOXO1	0.58	1.56	1.4274212	1.59E-09
396	9820	CUL7	6.06	16.29	1.4265969	1.84E-78
397	136541	PRSS58	2.51	6.72	1.4207739	4.47E-05
398	29775	CARD10	0.4	1.07	1.4195389	7.06E-05
399	347733	TUBB2B	0.88	2.35	1.4170853	8.67E-05
400	1843	DUSP1	7.82	20.88	1.4168812	4.70E-36
401	9751	SNPH	1.51	4.03	1.4162313	2.01E-19
402	8412	BCAR3	0.18	0.48	1.4150375	0.025136947
403	55753	OGDHL	0.3	0.8	1.4150375	0.001379005
404	43	ACHE	0.36	0.96	1.4150375	0.005557307
405	2634	GBP2	0.44	1.17	1.4109331	2.82E-05
406	9643	MORF4L2	46.24	122.79	1.4089798	2.89E-190
407	65124	SOWAHC	0.23	0.61	1.4071754	0.001972895
408	55693	KDM4D	0.23	0.61	1.4071754	0.015666967
409	91828	EXOC3L4	2.32	6.13	1.4017623	1.59E-16
410	658	BMPR1B	0.19	0.5	1.3959287	0.002796079
411	647042	GOLGA6L10	0.97	2.55	1.3944406	9.81E-04
412	7286	TUFT1	0.85	2.23	1.391509	8.47E-07
413	23428	SLC7A8	2.57	6.74	1.3909802	1.48E-18
414	441525	SPANXN4	3.05	7.99	1.3893863	0.027962718
415	163259	DENND2C	0.68	1.78	1.3882706	3.38E-11
416	7755	ZNF205	1.81	4.73	1.3858505	5.09E-09
417	4192	MDK	146.68	383.1	1.3850489	2.49E-211
418	142678	MIB2	3.45	9.01	1.3849307	4.80E-23
419	110116772	ZNF765-ZNF761	0.54	1.41	1.3846639	8.71E-08
420	51390	AIG1	2	5.22	1.3840498	0.035670063
421	10501	SEMA6B	2.76	7.17	1.3773049	1.20E-24
422	8352	H3C3	4.35	11.3	1.3772355	0.00441693
423	27242	TNFRSF21	0.52	1.35	1.3763759	0.007582528
424	9658	ZNF516	1.78	4.62	1.3760156	5.02E-17

425	10669	CGREF1	0.37	0.96	1.3755091	0.017433583
426	123688	HYKK	2.17	5.63	1.3754399	3.37E-04
427	79778	MICALL2	9.43	24.37	1.3697766	7.68E-67
428	222962	SLC29A4	0.84	2.17	1.3692338	7.88E-06
429	6038	RNASE4	0.33	0.85	1.3649968	0.031739923
430	51252	FAM178B	5.83	15.01	1.3643562	4.31E-09
431	5129	CDK18	5.38	13.84	1.3631659	2.42E-36
432	146330	FBXL16	0.21	0.54	1.3625701	0.014044527
433	29906	ST8SIA5	0.58	1.49	1.3611875	0.008730019
434	154661	RUNDC3B	0.37	0.95	1.3604022	5.39E-05
435	84210	ANKRD20A1	1.13	2.89	1.3547467	2.03E-10
436	3270	HRC	1.71	4.37	1.353637	1.06E-08
437	100820829	MYZAP	110.09	281.12	1.3525027	0
438	100526772	STIMATE-MUSTN1	1.2	3.06	1.3504972	3.97E-04
439	1052	CEBPD	2.74	6.97	1.3469828	4.93E-07
440	79955	PDZD7	0.48	1.22	1.3457748	0.013190614
441	80119	PIF1	11.94	30.34	1.3454182	6.24E-66
442	23645	PPP1R15A	73.81	187.35	1.3438478	0
443	2913	GRM3	0.58	1.47	1.3416914	4.52E-05
444	1466	CSRP2	2.21	5.6	1.3413805	4.98E-04
445	140576	S100A16	0.75	1.9	1.3410369	0.019934536
446	54922	RASIP1	2.16	5.47	1.3405095	2.62E-15
447	6794	STK11	26.87	68.02	1.3399627	1.12E-184
448	3008	H1-4	2.34	5.92	1.3390886	0.001400645
449	6237	RRAS	34.85	88.16	1.3389656	2.88E-56
450	148229	ATP8B3	0.19	0.48	1.337035	0.015664833
451	112268350	LOC112268350	0.21	0.53	1.335603	0.040233183
452	928	CD9	55.08	138.93	1.3347577	5.58E-119
453	84787	KMT5C	12.6	31.74	1.3328784	1.35E-53
454	114134	SLC2A13	1.38	3.47	1.3302674	0.041840735
455	10309	CCNO	1.11	2.79	1.3297054	0.001400875
456	23409	SIRT4	0.43	1.08	1.3286227	0.044567499
457	255758	TCTEX1D2	2.66	6.68	1.3284219	0.00623959
458	83999	KREMEN1	2.58	6.46	1.3241631	4.75E-34
459	9510	ADAMTS1	1.43	3.58	1.3239444	3.11E-16
460	130951	M1AP	0.36	0.9	1.3219281	0.031743971
461	255394	TCP11L2	0.32	0.8	1.3219281	0.015561194
462	3782	KCNN3	0.12	0.3	1.3219281	2.82E-04
463	4783	NFIL3	4.36	10.89	1.3206039	8.46E-18
464	7328	UBE2H	6.05	15.07	1.3166724	1.12E-64
465	90390	MED30	25.13	62.59	1.3165215	1.51E-39
466	113452	TMEM54	14.21	35.38	1.3160275	1.10E-23
467	219833	C11orf45	0.6	1.49	1.3122779	4.52E-05
468	200879	LIPH	2.94	7.3	1.3120803	5.69E-22
469	1649	DDIT3	19.6	48.66	1.3118827	9.03E-29
470	64746	ACBD3	9.07	22.51	1.3113916	3.18E-65
471	64400	AKTIP	1.31	3.25	1.3108729	3.35E-05
472	55034	MOCOS	0.5	1.24	1.3103401	4.38E-07
473	5795	PTPRJ	0.82	2.03	1.3077839	6.67E-05
474	5333	PLCD1	3.58	8.86	1.3073471	1.62E-19
475	7869	SEMA3B	0.49	1.21	1.3041534	0.01319245
476	221908	PPP1R35	20.18	49.83	1.3040884	5.06E-30
477	9659	PDE4DIP	13.88	34.26	1.3035176	1.09E-64
478	8996	NOL3	2.47	6.09	1.3019312	2.42E-07
479	11092	SPACA9	1.3	3.2	1.2995603	0.011616631
480	10848	PPP1R13L	1.66	4.08	1.2973859	2.11E-09
481	203328	SUSD3	11.33	27.84	1.2970114	4.28E-23
482	100526761	CCDC169-SOHLH2	1.36	3.34	1.2962415	1.59E-07
483	146664	MGAT5B	0.11	0.27	1.2954559	0.045001002
484	51313	GASK1B	0.38	0.93	1.2912313	1.42E-04
485	8971	H1-10	138.57	337.81	1.2855971	0

486	399668	SMIM10L2A	0.16	0.39	1.2854022	0.015563315
487	23005	MAPKBP1	5.25	12.75	1.2801079	2.76E-74
488	55565	ZNF821	2.55	6.18	1.2771096	3.84E-09
489	146909	KIF18B	19.87	48.12	1.2760448	5.46E-151
490	7291	TWIST1	0.69	1.67	1.2751798	0.008699454
491	23753	SDF2L1	36.74	88.91	1.2749941	9.72E-41
492	284131	ENDOV	1.6	3.87	1.2742617	0.003284085
493	10484	SEC23A	10.13	24.46	1.2717902	2.40E-55
494	8795	TNFRSF10B	1.43	3.45	1.2705812	2.56E-11
495	3600	IL15	1.77	4.27	1.2704867	9.54E-08
496	1832	DSP	1.02	2.46	1.2700892	5.57E-18
497	347736	NME9	0.42	1.01	1.2658941	0.019478463
498	84258	SYT3	0.57	1.37	1.2651421	7.88E-04
499	7942	TFEB	3.71	8.9	1.2623861	2.19E-16
500	57648	KIAA1522	18.77	44.94	1.2595715	2.05E-130
501	58526	MID1IP1	41.48	99.22	1.2582151	1.61E-171
502	351	APP	1.23	2.94	1.2571578	3.00E-04
503	23149	FCHO1	3.13	7.48	1.2568756	1.72E-17
504	79363	CPLANE2	0.98	2.34	1.2556549	0.0024208
505	85302	FBF1	1.78	4.25	1.2555856	5.58E-16
506	23551	RASD2	0.21	0.5	1.2515388	0.019929215
507	3107	HLA-C	318.14	757.17	1.2509555	0
508	374887	YJEFN3	2.47	5.87	1.2488495	2.66E-04
509	90362	FAM110B	4.27	10.13	1.2463262	6.51E-26
510	112268052	LOC112268052	0.71	1.68	1.2425703	0.028034466
511	10817	FRS3	3.78	8.94	1.2418886	1.40E-12
512	151011	SEPTIN10	7.43	17.55	1.2400369	5.75E-34
513	7922	SLC39A7	60.03	141.37	1.2357204	1.61E-200
514	83874	TBC1D10A	2.76	6.49	1.2335502	1.41E-09
515	80325	ABTB1	3.42	8.04	1.2331992	1.51E-11
516	10298	PAK4	16.52	38.82	1.2325864	4.20E-81
517	9783	RIMS3	6.1	14.33	1.2321575	1.27E-79
518	63971	KIF13A	9.51	22.33	1.231466	7.89E-04
519	1757	SARDH	0.52	1.22	1.2302976	0.001053929
520	221	ALDH3B1	0.84	1.97	1.2297344	1.21E-04
521	339210	C17orf67	0.87	2.04	1.2294818	0.001053752
522	828	CAPS	0.64	1.5	1.2288187	0.021682265
523	27303	RBMS3	0.68	1.59	1.2254201	1.55E-07
524	11015	KDELR3	7.91	18.49	1.2249956	8.31E-15
525	100528064	NEDD8-MDP1	3.57	8.33	1.2223924	3.34E-04
526	153	ADRB1	0.36	0.84	1.2223924	0.010626041
527	84264	HAGHL	9.58	22.35	1.2221773	1.11E-17
528	51439	FAM8A1	7.49	17.44	1.2193624	7.02E-61
529	6352	CCL5	1.72	4	1.2175914	4.85E-04
530	8527	DGKD	18.06	41.99	1.2172479	3.87E-198
531	63924	CIDEC	0.96	2.23	1.2159374	0.034164111
532	51312	SLC25A37	95.26	221.25	1.215735	0
533	124401	ANKS3	2.96	6.87	1.2147129	1.02E-08
534	157378	TMEM65	6.2	14.38	1.2137236	2.67E-45
535	27338	UBE2S	130.6	302.8	1.2132103	0
536	8331	H2AC14	3.18	7.37	1.2126379	0.044572994
537	56413	LTB4R2	2.24	5.19	1.2122358	5.06E-06
538	8541	PPFIA3	6.96	16.1	1.2099015	1.23E-54
539	100316904	SAP25	4.51	10.43	1.2095398	2.56E-08
540	10468	FST	1.12	2.59	1.2094534	0.007513489
541	84261	FBXW9	3.17	7.33	1.2093304	9.36E-09
542	883	KYAT1	8.36	19.33	1.2092668	6.97E-25
543	2109	ETFB	37.77	87.3	1.2087409	6.49E-40
544	54997	TESC	128.64	296.95	1.2068807	8.72E-161
545	7263	TST	5.82	13.42	1.2052936	1.62E-09
546	5996	RGS1	2.36	5.44	1.2048198	2.33E-05

547	1051	CEBPB	17.42	40.12	1.203577	7.38E-49
548	57719	ANO8	3.01	6.93	1.2030919	9.49E-24
549	79144	PPDPF	114.52	263.54	1.2024224	4.54E-109
550	402573	C7orf61	1.5	3.45	1.2016339	0.00931381
551	9021	SOCS3	32.14	73.83	1.1998372	1.37E-137
552	92797	HELB	0.95	2.18	1.1983287	1.52E-05
553	92960	PEX11G	0.92	2.11	1.1975372	0.046856198
554	100996928	FMC1-LUC7L2	3.67	8.41	1.1963257	2.18E-16
555	54805	CNNM2	3.14	7.19	1.1952272	2.57E-16
556	9811	CTIF	3.28	7.51	1.1951171	2.58E-32
557	225689	MAPK15	0.38	0.87	1.195016	0.019931875
558	64856	VWA1	0.21	0.48	1.1926451	0.035504203
559	29899	GPSM2	6.56	14.97	1.1903065	6.77E-42
560	200958	MUC20	0.32	0.73	1.1898246	0.039028119
561	11093	ADAMTS13	0.18	0.41	1.187627	0.028030843
562	57511	COG6	8.48	19.3	1.1864647	1.91E-48
563	59084	ENPP5	1.2	2.73	1.1858665	5.82E-08
564	868	CBLB	1.92	4.36	1.1832218	3.98E-19
565	127687	C1orf122	32.7	74.13	1.1807669	2.42E-50
566	4804	NGFR	1.35	3.06	1.1805722	5.83E-08
567	1039	CDR2	11.69	26.48	1.1796282	1.60E-47
568	339122	RAB43	1.4	3.17	1.179056	5.40E-11
569	64064	OXCT2	1.29	2.92	1.1785973	3.11E-04
570	79845	RNF122	3.82	8.64	1.1774587	5.37E-11
571	51696	HECA	7.5	16.96	1.1771737	1.27E-67
572	101060321	TBC1D3G	1.15	2.6	1.1768778	5.49E-08
573	90249	UNC5A	0.23	0.52	1.1768778	0.029841303
574	3619	INCENP	20.74	46.84	1.1753252	2.70E-125
575	283152	CCDC153	3.3	7.45	1.1747744	9.82E-07
576	283248	RCOR2	3.93	8.87	1.1744048	6.80E-18
577	144608	C12orf60	1.39	3.13	1.1710778	0.024224126
578	1316	KLF6	2.23	5.02	1.1706437	1.47E-16
579	400916	CHCHD10	89.88	202.28	1.1702817	7.70E-62
580	115704	EVI5L	2.45	5.51	1.1692706	2.81E-15
581	5002	SLC22A18	1.37	3.08	1.1687545	5.48E-04
582	57821	CCDC181	0.45	1.01	1.1663584	0.046850465
583	64386	MMP25	0.33	0.74	1.1650592	0.021685139
584	9531	BAG3	12.93	28.96	1.1633393	3.13E-48
585	160364	CLEC12A	1.68	3.76	1.1622714	7.77E-04
586	25766	PRPF40B	1.65	3.69	1.1611548	3.82E-09
587	11037	STON1	2.33	5.21	1.1609534	9.78E-21
588	9060	PAPSS2	5.86	13.1	1.1605942	1.02E-32
589	2260	FGFR1	0.17	0.38	1.1604647	0.047809928
590	23224	SYNE2	0.34	0.76	1.1604647	0.007146877
591	7781	SLC30A3	14.17	31.63	1.1584538	4.07E-50
592	57524	CASKIN1	0.8	1.78	1.1538053	4.53E-08
593	10312	TCIRG1	26.01	57.87	1.1537492	7.06E-109
594	5027	P2RX7	0.95	2.11	1.1512436	2.25E-08
595	51379	CRLF3	16.14	35.83	1.1505275	3.01E-66
596	9605	VPS9D1	1.56	3.46	1.149226	2.57E-05
597	5238	PGM3	5.16	11.44	1.1486441	6.08E-28
598	22839	DLGAP4	18.86	41.8	1.1481733	2.17E-84
599	7137	TNNI3	9.63	21.33	1.1472763	2.71E-09
600	6303	SAT1	24.72	54.75	1.1471802	4.71E-33
601	6535	SLC6A8	2.33	5.15	1.1442425	5.28E-15
602	7262	PHLDA2	2.79	6.16	1.1426652	0.00116212
603	3977	LIFR	0.29	0.64	1.142019	1.22E-05
604	3490	IGFBP7	0.88	1.94	1.1404812	0.026846643
605	112268238	LOC112268238	1.38	3.04	1.1394031	6.57E-04
606	7220	TRPC1	3.07	6.76	1.1387846	1.17E-19
607	85456	TNKS1BP1	9.19	20.23	1.1383596	4.94E-79

608	55227	LRRC1	2.49	5.48	1.1380302	1.15E-12
609	56935	SMCO4	5.59	12.3	1.1377381	9.73E-06
610	1645	AKR1C1	4.2	9.24	1.1375035	3.82E-29
611	7439	BEST1	0.5	1.1	1.1375035	4.83E-04
612	8744	TNFSF9	10.1	22.21	1.1368541	1.24E-21
613	255783	INAFM1	13.48	29.64	1.136725	2.74E-12
614	131540	ZDHHC19	0.96	2.11	1.1361367	0.040758978
615	84727	SPSB2	6.01	13.2	1.135101	1.69E-10
616	583	BBS2	3.63	7.97	1.1346102	6.02E-15
617	29953	TRHDE	0.36	0.79	1.1338557	8.15E-04
618	10826	FAXDC2	13.09	28.71	1.1330882	3.07E-53
619	78990	OTUB2	0.26	0.57	1.1324503	0.022744278
620	10360	NPM3	25.82	56.59	1.1320581	7.09E-24
621	376497	SLC27A1	1.34	2.93	1.1286677	2.07E-07
622	501	ALDH7A1	0.97	2.12	1.1280076	2.07E-07
623	3014	H2AX	137.14	299.69	1.1278215	4.21E-269
624	200942	KLHDC8B	21.82	47.67	1.1274305	2.16E-56
625	653082	ZDHHC11B	0.38	0.83	1.1271119	0.049572999
626	10536	P3H3	9.24	20.18	1.1269614	3.47E-33
627	30812	SOX8	0.33	0.72	1.1255309	0.03086071
628	3913	LAMB2	8.2	17.89	1.1254576	9.50E-66
629	388272	C16orf87	6.35	13.85	1.1250575	4.56E-05
630	140606	SELENOM	18.74	40.83	1.1235086	2.94E-12
631	11076	TPPP	1.07	2.33	1.1227192	6.18E-10
632	8612	PLPP2	1.13	2.46	1.1223355	0.015066415
633	84717	HDGFL2	34.5	75.09	1.1220244	3.34E-103
634	80223	RAB11FIP1	5.14	11.18	1.1210799	3.31E-37
635	157638	LRATD2	0.23	0.5	1.1202942	0.013188777
636	145853	C15orf61	6.86	14.91	1.1199998	2.90E-40
637	2548	GAA	0.99	2.15	1.1188362	1.17E-05
638	158056	MAMDC4	0.41	0.89	1.1181814	0.006459459
639	27352	SGSM3	13.96	30.3	1.1180189	2.78E-56
640	400709	SIGLEC16	0.77	1.67	1.1169178	6.15E-05
641	3690	ITGB3	32.38	70.21	1.1165735	1.31E-268
642	54557	SGTB	2.44	5.28	1.1136568	3.15E-19
643	421	ARVCF	3.58	7.74	1.112374	1.26E-17
644	1028	CDKN1C	1.05	2.27	1.112303	0.00318419
645	84519	ACRBP	4.72	10.2	1.1117104	5.89E-12
646	8820	HESX1	0.5	1.08	1.1110313	0.036002072
647	84310	C7orf50	33.92	73.22	1.1101016	1.09E-53
648	93233	CCDC114	0.45	0.97	1.1080597	0.0059555
649	642475	MROH6	5.73	12.35	1.107904	8.30E-20
650	23242	COBL	0.39	0.84	1.1069152	1.79E-04
651	57568	SIPA1L2	0.13	0.28	1.1069152	0.047804099
652	2053	EPHX2	8.73	18.8	1.1066791	8.10E-24
653	92017	SNX29	0.85	1.83	1.1063089	0.032211507
654	10841	FTCD	0.33	0.71	1.105353	0.047815759
655	55529	PIP4P2	20.68	44.48	1.1049206	2.91E-18
656	5916	RARG	7.23	15.55	1.104847	1.99E-27
657	54540	FAM193B	23.54	50.58	1.1034527	8.29E-98
658	414060	TBC1D3C	1.55	3.33	1.103254	1.07E-06
659	4013	VWA5A	12.61	27.05	1.1010603	3.35E-33
660	152189	CMTM8	28.28	60.65	1.1007255	6.42E-35
661	2273	FHL1	0.49	1.05	1.0995357	0.019854817
662	79777	ACBD4	5.74	12.3	1.0995357	6.55E-12
663	254552	NUDT8	8.53	18.26	1.0980691	2.05E-06
664	57030	SLC17A7	1.43	3.06	1.0975165	2.62E-06
665	5660	PSAP	63.43	135.73	1.0975024	3.71E-220
666	64927	TTC23	3.15	6.73	1.0952547	6.21E-15
667	26575	RGS17	3.42	7.3	1.0939001	0.002941758
668	3155	HMGCL	9.15	19.53	1.0938483	3.54E-14

669	29942	PURG	1.64	3.5	1.0936591	3.49E-05
670	54058	C21orf58	35.95	76.72	1.093611	3.66E-81
671	338707	B4GALNT4	1.37	2.92	1.0917925	1.98E-07
672	26013	L3MBTL1	3.16	6.73	1.0906819	5.18E-14
673	57664	PLEKHA4	4.07	8.66	1.0893382	5.55E-16
674	83937	RASSF4	0.94	2	1.0892673	2.64E-05
675	7227	TRPS1	0.08	0.17	1.0874628	0.046844733
676	10603	SH2B2	0.64	1.36	1.0874628	0.009726817
677	6654	SOS1	23.9	50.75	1.0863972	9.83E-208
678	54492	NEURL1B	1.8	3.82	1.0855757	6.62E-17
679	84266	ALKBH7	40.9	86.76	1.0849292	1.22E-41
680	79746	ECHDC3	0.99	2.1	1.0848889	0.013360186
681	11119	BTN3A1	7.53	15.97	1.0846425	5.31E-33
682	116832	RPL39L	2.49	5.28	1.0843922	0.020289411
683	90353	CTU1	4.67	9.9	1.084006	1.23E-12
684	7423	VEGFB	36.08	76.43	1.0829397	2.14E-74
685	55502	HES6	14.14	29.92	1.0813281	1.86E-21
686	3758	KCNJ1	0.52	1.1	1.08092	0.023335893
687	1647	GADD45A	129.42	273.5	1.0794803	1.51E-186
688	23022	PALLD	4.5	9.5	1.0780025	2.44E-30
689	230	ALDOC	54.62	115.29	1.0777662	1.90E-99
690	333	APLP1	2.8	5.91	1.0777313	7.71E-10
691	57558	USP35	3.15	6.64	1.0758314	7.03E-16
692	129642	MBOAT2	25.86	54.46	1.0744747	6.00E-56
693	684	BST2	20.42	43	1.0743538	2.66E-20
694	125488	TTC39C	6.57	13.83	1.0738359	0.005259455
695	80139	ZNF703	0.68	1.43	1.0724085	0.001220767
696	6446	SGK1	0.99	2.08	1.0710831	0.001220565
697	9322	TRIP10	34.43	72.32	1.0707285	3.19E-82
698	10332	CLEC4M	0.6	1.26	1.0703893	0.041517569
699	5127	CDK16	31.19	65.48	1.0699708	2.57E-115
700	91523	PCED1B	3.8	7.97	1.0685803	2.40E-11
701	54795	TRPM4	5.19	10.86	1.0652177	3.22E-24
702	90326	THAP3	14.91	31.19	1.0648033	4.52E-20
703	2323	FLT3LG	14.58	30.49	1.0643454	4.13E-13
704	3911	LAMA5	8.02	16.77	1.0642085	9.77E-116
705	1292	COL6A2	2.84	5.93	1.0621412	3.97E-12
706	10954	PDIA5	10.88	22.69	1.0603781	9.76E-23
707	8969	H2AC11	5.12	10.66	1.0579917	0.027094725
708	84693	MCEE	10.16	21.15	1.0577573	3.78E-08
709	5698	PSMB9	94.7	197.08	1.057345	1.51E-89
710	871	SERPINH1	48.75	101.41	1.0567258	2.85E-111
711	29957	SLC25A24	4	8.32	1.0565835	3.50E-21
712	79161	TMEM243	2.21	4.59	1.0544478	3.55E-04
713	57156	TMEM63C	0.26	0.54	1.0544478	0.013362041
714	342918	C19orf81	12.89	26.75	1.0532866	1.01E-08
715	84667	HES7	5.09	10.56	1.0528723	5.72E-12
716	84278	MFSD14C	10.46	21.7	1.0528122	0.003808575
717	64748	PLPPR2	1.48	3.07	1.0526415	1.44E-05
718	7133	TNFRSF1B	1.08	2.24	1.0524674	1.94E-05
719	387787	LIPT2	0.95	1.97	1.0521962	0.00215065
720	8544	PIR	6.54	13.55	1.0509303	4.52E-09
721	55336	FBXL8	0.99	2.05	1.0501235	0.013358331
722	100302736	TMED7-TICAM2	1.28	2.65	1.0498485	1.05E-06
723	348	APOE	70.25	145.43	1.0497548	2.09E-79
724	6455	SH3GL1	27.19	56.26	1.0490334	2.26E-82
725	10221	TRIB1	24.72	51.14	1.0487734	9.48E-124
726	116931	MED12L	6.72	13.9	1.0485517	2.30E-72
727	114793	FMNL2	0.74	1.53	1.0479345	4.18E-06
728	9819	TSC22D2	5.04	10.42	1.0478596	3.22E-13
729	91404	SESTD1	8.09	16.72	1.0473632	3.44E-113

730	9723	SEMA3E	0.3	0.62	1.0473057	0.001633092
731	107986211	LOC107986211	0.3	0.62	1.0473057	0.003764567
732	23768	FLRT2	0.15	0.31	1.0473057	0.0233756
733	55367	PIDD1	8.76	18.1	1.0469869	1.22E-25
734	79788	ZNF665	0.31	0.64	1.0458037	0.026982191
735	29841	GRHL1	1.57	3.24	1.0452293	1.77E-07
736	282679	AQP11	0.95	1.96	1.0448542	0.005738568
737	130888	FBXO36	0.82	1.69	1.0433274	0.001065249
738	9253	NUMBL	5.61	11.56	1.0430687	1.16E-23
739	50488	MINK1	16.02	33.01	1.043029	4.26E-95
740	8110	DPF3	12.63	26	1.041657	1.19E-04
741	8312	AXIN1	24.87	51.13	1.0397635	1.24E-80
742	9518	GDF15	61.36	126.14	1.0396555	1.69E-70
743	102723713	LOC102723713	0.36	0.74	1.0395284	0.035853188
744	255738	PCSK9	7.28	14.95	1.0381351	8.01E-31
745	26502	NARF	35.09	72.05	1.0379385	8.35E-62
746	9649	RALGPS1	0.57	1.17	1.0374747	0.001640242
747	388341	LRRC75A	49.04	100.65	1.0373163	3.12E-178
748	3398	ID2	87.57	179.6	1.0362787	2.99E-109
749	83463	MXD3	20.73	42.51	1.0360821	4.53E-20
750	10802	SEC24A	9.89	20.28	1.0360152	2.69E-40
751	63941	NECAB3	14.84	30.42	1.0355291	4.45E-16
752	254531	LPCAT4	13.58	27.83	1.0351574	2.15E-27
753	4281	MID1	1.02	2.09	1.0349338	7.57E-06
754	6238	RRBP1	30.89	63.17	1.0320997	1.78E-182
755	79767	ELMO3	1.13	2.31	1.0315701	0.001880617
756	149233	IL23R	1.86	3.8	1.0306968	8.26E-07
757	8329	H2AC13	11.11	22.67	1.0289256	9.38E-04
758	115098	CCDC124	114.08	232.75	1.0287353	4.62E-97
759	80303	EFHD1	1.52	3.1	1.0281969	5.27E-04
760	168455	CCDC71L	1.6	3.26	1.0268001	2.37E-13
761	51171	HSD17B14	4.3	8.76	1.0265942	6.73E-05
762	8322	FZD4	2.45	4.99	1.0262581	1.12E-21
763	645051	GAGE13	12.8	26.02	1.0234772	5.87E-05
764	23414	ZFPM2	3.41	6.93	1.0230836	5.63E-18
765	29998	BICRA	3.63	7.37	1.0216951	2.26E-21
766	113675	SDSL	5.61	11.37	1.0191596	9.25E-08
767	3110	MNX1	2.8	5.67	1.0179219	3.56E-07
768	9351	SLC9A3R2	10.18	20.6	1.0169068	2.82E-23
769	147906	DACT3	4.76	9.63	1.0165742	1.85E-16
770	54845	ESRP1	0.44	0.89	1.0163018	0.011616535
771	79174	CRELD2	6.79	13.73	1.0158481	8.66E-08
772	9828	ARHGEF17	1.88	3.8	1.0152668	4.10E-18
773	2812	GP1BB	100.35	202.75	1.0146613	2.54E-79
774	10398	MYL9	5.18	10.46	1.0138588	6.69E-16
775	25805	BAMBI	19.66	39.69	1.0135122	7.08E-33
776	5033	P4HA1	9.87	19.88	1.0101958	5.16E-30
777	107987373	LOC107987373	6.44	12.97	1.0100459	2.69E-04
778	9950	GOLGA5	9.94	20.01	1.0094034	1.73E-30
779	200312	RNF215	3.09	6.21	1.0069864	4.79E-07
780	7692	ZNF133	2.4	4.82	1.0059987	0.022468929
781	7469	NELFA	40.12	80.57	1.0059212	2.45E-88
782	7461	CLIP2	7.45	14.96	1.0057978	2.81E-45
783	83719	YPEL3	34.75	69.7	1.0041457	1.64E-31
784	3720	JARID2	15	30.08	1.0038421	1.37E-66
785	440295	GOLGA6L9	3.83	7.68	1.0037619	1.76E-19
786	64077	LHPP	16.53	33.13	1.0030515	7.10E-24
787	122509	IFI27L1	10.48	21	1.0027506	7.59E-09
788	9479	MAPK8IP1	2.73	5.47	1.0026399	1.74E-09
789	9853	RUSC2	6.46	12.94	1.0022315	2.07E-26
790	79629	OCEL1	13.82	27.67	1.001565	1.80E-12

791	644815	FAM83G	0.31	0.62	1	0.011614903
792	5727	PTCH1	0.16	0.32	1	0.026971697
793	3293	HSD17B3	1.69	3.38	1	0.016386451
794	138428	PTRH1	2.59	5.18	1	0.005765284
795	100533496	TVP23C-CDRT4	0.67	1.34	1	0.007600948
796	115207	KCTD12	0.24	0.48	1	0.015343367
797	4291	MLF1	4.05	8.1	1	5.31E-10
798	399473	SPRED3	0.37	0.74	1	0.008728767
799	10156	RASA4	4.01	8.02	1	4.24E-11
800	400745	SH2D5	0.96	1.92	1	7.68E-05
801	8671	SLC4A4	0.42	0.21	-1	0.006483476
802	10184	LHFPL2	4.46	2.23	-1	9.52E-13
803	5125	PCSK5	0.3	0.15	-1	0.010881319
804	93953	GCNA	0.88	0.44	-1	0.022397437
805	4922	NTS	91.55	45.77	-1.000158	1.42E-51
806	101928589	LOC101928589	6.09	3.04	-1.002371	1.07E-06
807	10099	TSPAN3	84.81	42.29	-1.003918	4.33E-77
808	11154	AP4S1	5.62	2.8	-1.005143	2.13E-11
809	112694756	LOC112694756	47.89	23.81	-1.008157	4.36E-59
810	1571	CYP2E1	5.07	2.52	-1.008562	4.45E-05
811	51154	MRTO4	41.12	20.43	-1.009151	5.20E-43
812	2114	ETS2	102.18	50.7	-1.011055	4.71E-195
813	9816	URB2	3.81	1.89	-1.011405	1.42E-12
814	55703	POLR3B	4.92	2.44	-1.011777	2.87E-12
815	7071	KLF10	25.21	12.48	-1.014378	2.02E-40
816	79905	TMC7	0.79	0.39	-1.018379	0.030648686
817	55207	ARL8B	36.96	18.24	-1.018859	1.82E-58
818	150290	DUSP18	0.73	0.36	-1.0199	0.00361812
819	9057	SLC7A6	13.75	6.78	-1.020074	1.10E-48
820	129401	NUP35	22.29	10.97	-1.022833	7.89E-18
821	3093	UBE2K	43.26	21.28	-1.023536	7.41E-126
822	80746	TSEN2	7.91	3.89	-1.023908	2.14E-09
823	6569	SLC34A1	9.87	4.85	-1.025065	8.81E-12
824	56144	PCDHA4	0.53	0.26	-1.027481	3.05E-04
825	1521	CTSW	146.41	71.82	-1.027557	1.59E-88
826	1269	CNR2	0.98	0.48	-1.029747	1.75E-04
827	199	AIF1	1117.17	547.11	-1.029946	1.01E-168
828	92745	SLC38A5	36.37	17.79	-1.031682	4.63E-36
829	51527	GSKIP	6.09	2.97	-1.035979	9.70E-08
830	814	CAMK4	1.11	0.54	-1.039528	5.76E-09
831	6916	TBXAS1	41.97	20.38	-1.042204	6.31E-35
832	114885	OSBPL11	1.98	0.96	-1.044394	3.24E-06
833	339829	CCDC39	0.66	0.32	-1.044394	0.022394481
834	55008	HERC6	3.57	1.73	-1.045152	1.43E-08
835	9508	ADAMTS3	7.94	3.84	-1.048033	4.84E-29
836	100506127	GVQW3	3.27	1.58	-1.049366	5.50E-18
837	6700	SPRR2A	8.37	4.04	-1.050872	0.003839181
838	23219	FBXO28	5.37	2.59	-1.05197	9.54E-18
839	474344	GIMAP6	7.59	3.66	-1.052256	5.65E-16
840	340156	MYLK4	1.91	0.92	-1.053867	3.96E-07
841	79072	FASTKD3	9.52	4.58	-1.055614	1.53E-13
842	753	LDLRAD4	10.5	5.05	-1.056034	5.04E-55
843	83886	PRSS27	3.39	1.63	-1.056413	0.014790577
844	375341	C3orf62	3.4	1.63	-1.060663	2.59E-08
845	58496	LY6G5B	21.37	10.24	-1.061371	8.55E-08
846	123103	KLHL33	1.44	0.69	-1.061401	4.46E-05
847	147007	TMEM199	8.33	3.99	-1.061928	1.38E-15
848	140862	ISM1	6	2.87	-1.063912	1.59E-10
849	23007	PLCH1	23.06	11.03	-1.06396	1.20E-89
850	51703	ACSL5	12.18	5.82	-1.065423	4.04E-24
851	3691	ITGB4	6.51	3.11	-1.065743	5.28E-23

852	1524	CX3CR1	6.44	3.07	-1.068822	1.67E-12
853	285313	IGSF10	9.87	4.7	-1.070389	2.20E-49
854	113419	TEX261	28.02	13.31	-1.073946	3.10E-56
855	51225	ABI3	3.2	1.52	-1.074001	0.001026549
856	64785	GINS3	11.77	5.59	-1.074194	2.09E-14
857	22809	ATF5	12.74	6.04	-1.076745	5.24E-15
858	6876	TAGLN	1.29	0.61	-1.08049	3.70E-04
859	7481	WNT11	7.41	3.5	-1.082119	9.11E-10
860	10197	PSME3	73.55	34.74	-1.082128	2.07E-139
861	283377	SPRYD4	1.44	0.68	-1.082462	4.68E-11
862	10446	LRRN2	6.72	3.17	-1.083978	1.01E-14
863	285440	CYP4V2	6.11	2.88	-1.085104	5.88E-18
864	8224	SYN3	1.55	0.73	-1.0863	1.66E-08
865	54534	MRPL50	10.05	4.73	-1.087283	6.11E-21
866	25791	NGEF	2.04	0.96	-1.087463	0.001723101
867	7473	WNT3	1.87	0.88	-1.087463	1.20E-04
868	92340	PRR29	6.7	3.15	-1.088809	1.21E-04
869	7128	TNFAIP3	21.02	9.87	-1.090641	2.26E-65
870	112441434	SHLD3	3.28	1.54	-1.090765	0.001125608
871	55851	PSENEN	28.65	13.44	-1.092002	6.21E-17
872	10799	RPP40	12.61	5.91	-1.093338	1.19E-06
873	10962	MLLT11	18.39	8.61	-1.094836	1.17E-27
874	30819	KCNIP2	6.29	2.94	-1.097244	7.79E-09
875	10849	POLR1G	4.11	1.92	-1.098032	8.35E-08
876	6996	TDG	28.77	13.44	-1.098032	5.73E-56
877	93589	CACNA2D4	6.98	3.26	-1.098355	7.57E-26
878	10220	GDF11	36.71	17.14	-1.098806	0
879	6752	SSTR2	0.3	0.14	-1.099536	0.027590197
880	5409	PNMT	27.52	12.81	-1.10321	2.18E-14
881	11251	PTGDR2	8.84	4.11	-1.104908	2.18E-16
882	26122	EPC2	14.5	6.73	-1.107374	1.14E-37
883	22858	CILK1	2.85	1.32	-1.110424	6.49E-12
884	9572	NR1D1	6.37	2.95	-1.110578	3.69E-11
885	259197	NCR3	29.6	13.66	-1.11564	2.13E-16
886	54978	SLC35F6	9.2	4.24	-1.11757	1.08E-23
887	114836	SLAMF6	18.4	8.48	-1.11757	2.89E-31
888	60496	AASDHPPT	34.41	15.84	-1.119256	7.67E-60
889	107986453	LOC107986453	3.02	1.39	-1.119464	9.53E-09
890	10219	KLRG1	15.24	7.01	-1.120377	2.30E-17
891	54982	CLN6	62.65	28.8	-1.121246	5.78E-85
892	79865	TREML2	24.34	11.18	-1.122409	2.73E-59
893	85458	DIXDC1	3.18	1.46	-1.123058	1.52E-11
894	158880	USP51	1.09	0.5	-1.124328	2.02E-04
895	23046	KIF21B	13.77	6.31	-1.125817	4.61E-88
896	79184	BRCC3	7.16	3.28	-1.126264	1.21E-21
897	284098	PIGW	9.5	4.35	-1.126912	1.09E-15
898	57102	C12orf4	9.82	4.49	-1.129008	8.69E-23
899	118980	SFXN2	13.77	6.29	-1.130397	5.47E-32
900	284067	CFAP97D1	0.81	0.37	-1.130397	0.007785872
901	54491	OTULINL	7.27	3.32	-1.130772	2.15E-19
902	197358	NLRC3	9.11	4.16	-1.130868	2.44E-39
903	54738	FEV	246.4	112.5	-1.131077	2.34E-196
904	6571	SLC18A2	22.43	10.24	-1.131214	3.35E-56
905	29015	SLC43A3	169.91	77.44	-1.13362	2.48E-278
906	102723553	SMIM11B	9.7	4.42	-1.133938	1.44E-04
907	54937	SOHLH2	2.81	1.28	-1.134426	1.25E-04
908	8815	BANF1	383.29	174.57	-1.134631	1.23E-130
909	83992	CTTNBP2	14.81	6.74	-1.135751	2.37E-69
910	10346	TRIM22	4.29	1.95	-1.137504	9.13E-09
911	1769	DNAH8	0.11	0.05	-1.137504	0.038777863
912	107984203	LOC107984203	6.03	2.74	-1.137982	1.66E-12

913	79071	ELOVL6	15.09	6.85	-1.139417	1.18E-67
914	7453	WARS1	115.25	52.29	-1.14016	7.71E-190
915	340024	SLC6A19	31.14	14.12	-1.141029	2.72E-108
916	4883	NPR3	3.51	1.59	-1.142444	8.97E-12
917	125893	ZNF816	2.43	1.1	-1.143453	6.81E-05
918	2359	FPR3	4.93	2.23	-1.144544	3.18E-09
919	376267	RAB15	13.06	5.9	-1.146368	3.26E-30
920	134957	STXBP5	38.44	17.33	-1.149337	1.07E-260
921	4638	MYLK	17.89	8.06	-1.150302	1.22E-37
922	7699	ZNF140	6.47	2.91	-1.152747	1.25E-12
923	6275	S100A4	898.33	404.02	-1.152819	5.39E-166
924	79850	TLCD3A	12.01	5.4	-1.153205	8.58E-18
925	3683	ITGAL	3.47	1.56	-1.15339	7.77E-14
926	9436	NCR2	2.67	1.2	-1.153805	0.004221314
927	285381	DPH3	8.5	3.82	-1.15389	1.34E-23
928	3561	IL2RG	124.65	55.96	-1.155415	1.13E-109
929	338596	ST8SIA6	127.78	57.36	-1.155545	0
930	5935	RBM3	53.2	23.86	-1.156832	3.72E-85
931	4914	NTRK1	19.51	8.75	-1.156859	3.48E-34
932	28978	TMEM14A	11.13	4.99	-1.157342	1.00E-06
933	10349	ABCA10	0.29	0.13	-1.157541	0.041110623
934	84517	ACTRT3	2.01	0.9	-1.159199	0.005606434
935	109504726	ERV3-1-ZNF117	1.68	0.75	-1.163499	1.29E-10
936	3695	ITGB7	4.01	1.79	-1.163643	2.89E-08
937	56154	TEX15	2.29	1.02	-1.166778	1.46E-19
938	118461	C10orf71	0.18	0.08	-1.169925	0.02146945
939	55709	KBTBD4	3.96	1.76	-1.169925	3.44E-07
940	159	ADSS2	28.92	12.82	-1.173671	1.87E-49
941	123722	FSD2	0.79	0.35	-1.174498	1.25E-04
942	2047	EPHB1	2.71	1.2	-1.175258	9.64E-10
943	55520	ELAC1	1.83	0.81	-1.17585	0.001437197
944	5778	PTPN7	99.21	43.91	-1.175936	9.69E-213
945	22997	IGSF9B	0.43	0.19	-1.178337	4.34E-06
946	101060179	LOC101060179	2.22	0.98	-1.179706	3.02E-04
947	64231	MS4A6A	3.83	1.69	-1.180321	1.74E-04
948	64236	PDLIM2	17.28	7.62	-1.18124	3.48E-15
949	317671	RFESD	8.3	3.65	-1.185215	1.76E-15
950	7562	ZNF708	4.3	1.89	-1.18595	2.42E-09
951	83759	RBM4B	11.22	4.92	-1.189342	1.19E-14
952	6282	S100A11	353.49	154.96	-1.189774	1.24E-80
953	26027	ACOT11	19.08	8.36	-1.190486	4.02E-37
954	153770	PLAC8L1	1.05	0.46	-1.190684	0.014277553
955	56652	TWNK	19.86	8.69	-1.192438	5.25E-44
956	58489	ABHD17C	1.51	0.66	-1.194011	0.002654689
957	128178	EDARADD	3.02	1.32	-1.194011	2.01E-07
958	10320	IKZF1	22.85	9.98	-1.195082	2.08E-92
959	8530	CST7	2.82	1.23	-1.197037	0.02920032
960	8871	SYNJ2	14.77	6.44	-1.197537	3.38E-81
961	1861	TOR1A	29.38	12.81	-1.197564	2.17E-41
962	65260	COA7	33.49	14.6	-1.197762	1.54E-95
963	8323	FZD6	8.56	3.73	-1.198435	9.77E-24
964	100532731	COMMD3-BMI1	7.07	3.08	-1.19878	1.10E-17
965	85446	ZFHX2	1.86	0.81	-1.199309	8.38E-12
966	84288	EFCAB2	6.04	2.63	-1.199486	2.55E-11
967	200205	IBA57	1.47	0.64	-1.199672	1.23E-09
968	3791	KDR	7.58	3.3	-1.199732	5.16E-33
969	23308	ICOSLG	3.31	1.44	-1.200762	2.87E-12
970	202374	STK32A	0.69	0.3	-1.201634	0.001168707
971	10870	HCST	245.49	106.7	-1.202104	2.02E-50
972	23604	DAPK2	1.91	0.83	-1.202389	0.001095915
973	100133941	CD24	7.88	3.42	-1.204199	3.44E-12

974	83593	RASSF5	21.11	9.16	-1.204507	4.40E-56
975	112495	GTF3C6	88.23	38.26	-1.205432	2.19E-36
976	57143	ADCK1	1.87	0.81	-1.207044	0.01468959
977	1846	DUSP4	1.87	0.81	-1.207044	1.19E-08
978	7957	EPM2A	2.45	1.06	-1.208717	4.90E-08
979	84216	TMEM117	1.92	0.83	-1.209923	3.19E-04
980	85481	PSKH2	2.76	1.19	-1.213707	6.89E-06
981	8091	HMGA2	2.32	1	-1.214125	7.33E-05
982	9770	RASSF2	14.81	6.38	-1.214943	1.18E-60
983	3269	HRH1	0.35	0.15	-1.222392	0.030925687
984	960	CD44	55.06	23.57	-1.224053	8.43E-184
985	340526	RTL5	1.22	0.52	-1.230298	8.35E-05
986	6745	SSR1	20.06	8.53	-1.233704	4.52E-151
987	23251	MINAR1	2.99	1.27	-1.235317	8.47E-18
988	149840	SHLD1	6.6	2.8	-1.237039	1.50E-04
989	7627	ZNF75A	17.52	7.42	-1.239512	2.46E-30
990	3579	CXCR2	3.77	1.59	-1.245538	7.54E-08
991	57545	CC2D2A	3.32	1.4	-1.245756	2.25E-05
992	57002	YAE1	8.98	3.77	-1.252151	5.23E-05
993	2014	EMP3	436.82	183.27	-1.253068	7.93E-140
994	107984745	LOC107984745	3.2	1.34	-1.255839	4.43E-09
995	7544	ZFY	2.46	1.03	-1.256014	2.06E-10
996	389421	LIN28B	22.44	9.39	-1.256876	3.16E-96
997	27340	UTP20	5.07	2.12	-1.257921	2.78E-37
998	79626	TNFAIP8L2	20.36	8.5	-1.260203	6.63E-16
999	2124	EVI2B	1.01	0.42	-1.265894	0.025216001
1000	5877	RABIF	4.69	1.95	-1.266114	4.92E-12
1001	9289	ADGRG1	53.37	22.16	-1.268071	9.11E-169
1002	6871	TADA2A	14.21	5.9	-1.26812	5.95E-26
1003	23650	TRIM29	0.94	0.39	-1.269187	0.033350784
1004	11182	SLC2A6	10.45	4.33	-1.271064	1.92E-22
1005	1183	CLCN4	14.69	6.08	-1.272691	1.19E-80
1006	23498	HAAO	0.63	0.26	-1.27684	0.006704195
1007	27181	SIGLEC8	1.85	0.76	-1.283454	4.34E-05
1008	163131	ZNF780B	4.36	1.79	-1.284369	1.13E-25
1009	10093	ARPC4	60.76	24.91	-1.286397	2.40E-63
1010	6404	SELPLG	66.82	27.27	-1.292965	4.62E-133
1011	150	ADRA2A	7.33	2.99	-1.293668	1.36E-23
1012	55612	FERMT1	2.8	1.14	-1.296393	1.26E-12
1013	10795	ZNF268	1.5	0.61	-1.298081	5.56E-18
1014	121340	SP7	0.69	0.28	-1.30117	0.016700232
1015	2811	GP1BA	103.25	41.89	-1.301464	3.42E-202
1016	105374103	LOC105374103	8.21	3.33	-1.30186	3.25E-34
1017	5791	PTPRE	1.16	0.47	-1.303392	3.40E-04
1018	196264	MPZL3	1.36	0.55	-1.306103	4.16E-05
1019	23209	MLC1	24.22	9.79	-1.306818	2.63E-71
1020	7582	ZNF33B	5.5	2.22	-1.308872	3.25E-29
1021	8778	SIGLEC5	3.06	1.23	-1.314873	2.74E-07
1022	401089	FOXL2NB	1.52	0.61	-1.31719	0.013921774
1023	492307	PPDPFL	16.13	6.47	-1.317909	6.77E-16
1024	79818	ZNF552	0.7	0.28	-1.321928	0.009522571
1025	284306	ZNF547	0.95	0.38	-1.321928	0.00542305
1026	3742	KCNA6	0.1	0.04	-1.321928	0.044096696
1027	102723750	LOC102723750	0.65	0.26	-1.321928	7.88E-04
1028	1427	CRYGS	2.23	0.89	-1.325166	0.046658319
1029	283571	PROX2	0.78	0.31	-1.331206	4.54E-04
1030	8620	NPFF	3.68	1.46	-1.333737	0.037983733
1031	64218	SEMA4A	3.53	1.4	-1.334241	5.39E-10
1032	1414	CRYBB1	5.41	2.14	-1.338018	9.80E-05
1033	51729	WBP11	59.58	23.56	-1.338489	1.09E-233
1034	1359	CPA3	34.35	13.56	-1.340953	1.83E-47

1035	83483	PLVAP	18.22	7.19	-1.341459	2.69E-34
1036	192683	SCAMP5	12.54	4.94	-1.343954	7.54E-29
1037	79081	LBHD1	8.16	3.21	-1.345996	8.28E-13
1038	107987254	LOC107987254	2.39	0.94	-1.346278	1.03E-05
1039	11102	RPP14	2.27	0.89	-1.350815	2.35E-09
1040	51807	TUBA8	2.17	0.85	-1.35216	2.55E-04
1041	4144	MAT2A	246.36	96.32	-1.354861	0
1042	84502	JPH4	2.18	0.85	-1.358793	6.23E-09
1043	7099	TLR4	1.77	0.69	-1.359081	2.25E-21
1044	113455421	DERPC	31.8	12.39	-1.359851	3.44E-77
1045	9941	EXOG	5.48	2.12	-1.370112	1.03E-16
1046	53833	IL20RB	0.83	0.32	-1.375039	0.031813313
1047	54809	SAMD9	6.64	2.56	-1.375039	9.34E-42
1048	110599588	ASDURF	16.55	6.37	-1.377466	7.16E-07
1049	255877	BCL6B	15.6	6	-1.378512	6.41E-49
1050	401258	RAB44	17.74	6.78	-1.387649	4.53E-69
1051	6285	S100B	2.8	1.07	-1.387816	0.00310097
1052	152007	GLIPR2	10.56	4.03	-1.389758	4.14E-17
1053	1534	CYB561	9.24	3.52	-1.392317	3.06E-25
1054	147657	ZNF480	4.41	1.68	-1.392317	4.57E-20
1055	219621	CABCOCO1	2.39	0.91	-1.393072	0.003101936
1056	92922	CCDC102A	6.37	2.42	-1.396286	2.48E-14
1057	389015	SLC9A4	0.87	0.33	-1.398549	4.54E-04
1058	10203	CALCRL	0.58	0.22	-1.398549	3.05E-04
1059	8905	AP1S2	13.6	5.14	-1.403766	6.19E-25
1060	100528030	POC1B-GALNT4	1.38	0.52	-1.408085	3.36E-07
1061	55915	LANCL2	2.1	0.79	-1.410465	2.03E-09
1062	2312	FLG	0.16	0.06	-1.415037	0.006705179
1063	619279	ZNF704	0.08	0.03	-1.415037	0.020346657
1064	23098	SARM1	0.24	0.09	-1.415037	0.003101453
1065	313	AOAH	0.24	0.09	-1.415037	0.034292818
1066	921	CD5	0.59	0.22	-1.423211	0.012001875
1067	9498	SLC4A8	1.61	0.6	-1.424026	3.03E-15
1068	2015	ADGRE1	0.51	0.19	-1.424498	0.031817369
1069	411	ARSB	2.8	1.04	-1.428843	4.81E-13
1070	25853	DCAF12	40.53	15.05	-1.429227	7.65E-136
1071	51206	GP6	86.87	32.25	-1.429559	1.98E-174
1072	80741	LY6G5C	6.79	2.52	-1.429988	4.52E-04
1073	6850	SYK	45.98	17.06	-1.430389	2.75E-231
1074	55184	DZANK1	0.89	0.33	-1.431339	5.24E-04
1075	5551	PRF1	14.89	5.5	-1.43684	1.15E-34
1076	8372	HYAL3	67.67	24.99	-1.437165	2.03E-107
1077	285852	TREML4	1.95	0.72	-1.437405	2.15E-05
1078	56243	KIAA1217	0.76	0.28	-1.440573	2.93E-06
1079	374383	NCR3LG1	17.17	6.3	-1.446466	9.00E-106
1080	81603	TRIM8	130.12	47.73	-1.446874	0
1081	64747	MFSD1	16.55	6.06	-1.449442	5.97E-34
1082	285349	ZNF660	1.59	0.58	-1.454902	4.28E-10
1083	117286	CIB3	2.6	0.94	-1.467779	0.015211328
1084	9437	NCR1	6.01	2.17	-1.46967	3.14E-07
1085	3955	LFNG	3.38	1.22	-1.470142	2.23E-08
1086	55313	CPPED1	35.32	12.69	-1.476793	4.16E-218
1087	79815	NIPAL2	2.65	0.95	-1.479993	1.36E-12
1088	84174	SLA2	30.21	10.83	-1.479993	2.79E-79
1089	22890	ZBTB1	9.24	3.3	-1.485427	2.11E-35
1090	147949	ZNF583	1.99	0.71	-1.486878	2.60E-08
1091	57559	STAMBPL1	3.03	1.08	-1.488286	8.12E-06
1092	132724	TMPRSS11B	0.59	0.21	-1.490326	0.021466601
1093	27094	KCNMB3	3.77	1.34	-1.492332	8.14E-06
1094	59283	CACNG8	2.48	0.88	-1.494765	1.52E-23
1095	5592	PRKG1	0.31	0.11	-1.494765	0.004357358

1096	1233	CCR4	6.33	2.24	-1.498707	1.77E-17
1097	1511	CTSG	1.67	0.59	-1.501061	0.047072784
1098	11314	CD300A	38.07	13.42	-1.50427	3.06E-48
1099	107987269	LOC107987269	0.74	0.26	-1.509014	0.047067032
1100	3581	IL9R	22.12	7.76	-1.511223	9.54E-74
1101	7177	TPSAB1	16.64	5.83	-1.513088	3.75E-17
1102	51555	PEX5L	0.2	0.07	-1.514573	0.006580353
1103	56983	POGLUT1	9.39	3.28	-1.517429	1.82E-32
1104	80774	LIMD2	160.7	56.12	-1.517783	2.63E-189
1105	5971	RELB	16.62	5.8	-1.518796	5.65E-37
1106	147040	KCTD11	0.43	0.15	-1.519374	0.031154975
1107	9836	LCMT2	2.87	1	-1.521051	1.33E-21
1108	57574	MARCHF4	2.9	1.01	-1.521698	1.13E-15
1109	164045	HFM1	0.69	0.24	-1.523562	0.002110537
1110	57514	ARHGAP31	1.18	0.41	-1.525091	5.88E-09
1111	197021	LCTL	0.49	0.17	-1.527247	0.025941348
1112	170575	GIMAP1	0.49	0.17	-1.527247	0.005490605
1113	29933	GPR132	1.04	0.36	-1.530515	8.34E-05
1114	286204	CRB2	0.55	0.19	-1.533432	3.75E-04
1115	8340	H2BC13	10.3	3.54	-1.540823	0.004356698
1116	441273	SPDYE2	1.95	0.67	-1.541241	9.23E-09
1117	8809	IL18R1	3.35	1.15	-1.542527	4.07E-15
1118	57191	VN1R1	1.14	0.39	-1.547488	0.014279517
1119	80008	TMEM156	6.79	2.32	-1.549287	8.50E-15
1120	80320	SP6	0.82	0.28	-1.550197	4.38E-04
1121	2207	FCER1G	142.38	48.51	-1.553392	4.23E-53
1122	124460	SNX20	1.91	0.65	-1.555061	1.38E-06
1123	653857	ACTR3C	1.62	0.55	-1.55849	0.013923696
1124	55256	ADI1	39.92	13.55	-1.558819	9.39E-85
1125	6948	TCN2	0.59	0.2	-1.560715	0.031147019
1126	57099	AVEN	10.4	3.52	-1.562936	8.34E-12
1127	107986113	LOC107986113	0.65	0.22	-1.562936	0.007194892
1128	597	BCL2A1	4.06	1.37	-1.567304	0.002425826
1129	84941	HSH2D	1.58	0.53	-1.57586	1.10E-05
1130	57699	CPNE5	8.93	2.99	-1.578515	1.84E-21
1131	8001	GLRA3	0.15	0.05	-1.584963	0.047078537
1132	9806	SPOCK2	0.42	0.14	-1.584963	0.008134097
1133	91768	CABLES1	0.24	0.08	-1.584963	0.031150996
1134	85477	SCIN	0.12	0.04	-1.584963	0.047084291
1135	1464	CSPG4	0.75	0.25	-1.584963	5.98E-08
1136	26154	ABCA12	0.15	0.05	-1.584963	0.017111681
1137	2709	GJB5	8.3	2.74	-1.598935	1.47E-11
1138	4057	LTF	0.85	0.28	-1.602036	0.002425441
1139	8743	TNFSF10	0.85	0.28	-1.602036	0.025944729
1140	149345	SHISA4	1.5	0.49	-1.614109	0.005206126
1141	220	ALDH1A3	0.4	0.13	-1.621488	0.020343946
1142	124637	CYB5D1	3.09	1	-1.627607	6.23E-13
1143	2342	FNTB	13.01	4.2	-1.63116	1.41E-39
1144	57103	TIGAR	0.31	0.1	-1.632268	3.71E-04
1145	146722	CD300LF	6.95	2.21	-1.652967	7.41E-14
1146	79628	SH3TC2	0.38	0.12	-1.662965	3.13E-13
1147	1270	CNTF	1.11	0.35	-1.665133	0.003389296
1148	165140	OXER1	1.08	0.34	-1.667425	0.00615382
1149	105376526	LOC105376526	8.07	2.52	-1.679145	3.93E-38
1150	344	APOC2	99.85	31.16	-1.680067	1.23E-50
1151	26960	NBEA	5.26	1.64	-1.681367	6.88E-24
1152	112267876	LOC112267876	3.66	1.14	-1.68281	0.009573566
1153	719	C3AR1	5.96	1.85	-1.687787	7.25E-25
1154	221468	TMEM217	8.67	2.69	-1.688426	1.21E-17
1155	2213	FCGR2B	2.94	0.91	-1.691878	1.32E-05
1156	151516	ASPRV1	1.83	0.56	-1.708345	1.96E-05

1157	79895	ATP8B4	8.69	2.64	-1.718818	3.33E-58
1158	2706	GJB2	0.83	0.25	-1.731183	0.003958375
1159	7730	ZNF177	1.2	0.36	-1.736966	1.53E-04
1160	57586	SYT13	0.3	0.09	-1.736966	0.00719594
1161	79858	NEK11	0.64	0.19	-1.752072	0.004604315
1162	57727	NCOA5	12.45	3.69	-1.754453	5.05E-51
1163	554	AVPR2	0.54	0.16	-1.754888	0.04410214
1164	114784	CSMD2	2.57	0.76	-1.757697	6.74E-49
1165	9379	NRXN2	7.21	2.13	-1.759146	1.64E-40
1166	341350	OVCH1	0.17	0.05	-1.765535	0.031143042
1167	202333	CMYA5	0.17	0.05	-1.765535	5.64E-04
1168	23430	TPSD1	7.4	2.17	-1.76983	3.43E-14
1169	83445	GSG1	0.58	0.17	-1.770518	0.009574931
1170	776	CACNA1D	0.24	0.07	-1.777608	0.002549429
1171	5031	P2RY6	0.96	0.28	-1.777608	0.014556706
1172	3680	ITGA9	0.83	0.24	-1.790077	1.25E-09
1173	23262	PPIP5K2	31.52	9.08	-1.795503	5.29E-235
1174	84951	TNS4	1.36	0.39	-1.802061	3.13E-09
1175	283420	CLEC9A	0.6	0.17	-1.819428	0.044091253
1176	10936	GPR75	1.95	0.55	-1.825971	5.94E-06
1177	7157	TP53	40.83	11.41	-1.839331	1.68E-133
1178	4613	MYCN	26.9	7.41	-1.860061	2.59E-71
1179	946	SIGLEC6	56.09	15.45	-1.860137	1.98E-240
1180	85414	SLC45A3	43.08	11.84	-1.863349	3.62E-198
1181	100506115	PELATON	0.66	0.18	-1.874469	0.015213406
1182	10628	TXNIP	56.91	15.39	-1.886689	6.10E-223
1183	1043	CD52	45.82	12.37	-1.889132	1.10E-14
1184	388813	LOC388813	3.71	1	-1.891419	1.16E-08
1185	55911	APOBR	17.82	4.8	-1.892391	8.47E-95
1186	51733	UPB1	1.19	0.32	-1.894818	0.002031728
1187	64499	TPSB2	12.37	3.32	-1.89759	1.08E-17
1188	5287	PIK3C2B	0.82	0.22	-1.89812	2.06E-09
1189	330	BIRC3	6.04	1.62	-1.898555	8.85E-61
1190	6094	ROM1	0.86	0.23	-1.902703	0.027852942
1191	3903	LAIR1	11.32	3.01	-1.911039	4.30E-27
1192	105379561	LOC105379561	2.07	0.55	-1.912127	4.94E-04
1193	255809	C19orf38	1.28	0.34	-1.912537	0.01520925
1194	100131244	ANKRD63	2.62	0.69	-1.924899	9.63E-17
1195	317749	DHRS4L2	2.48	0.65	-1.931828	1.24E-04
1196	3356	HTR2A	0.84	0.22	-1.932886	2.97E-07
1197	388419	BTBD17	0.84	0.22	-1.932886	0.013180769
1198	7031	TFF1	16.63	4.34	-1.938021	1.04E-06
1199	9573	GDF3	4.38	1.12	-1.967432	1.29E-07
1200	374393	FAM111B	4.37	1.11	-1.977074	1.07E-22
1201	7294	TXK	13.46	3.41	-1.980835	4.48E-107
1202	2998	GYS2	1.71	0.43	-1.991588	1.40E-06
1203	284402	SCGB2B2	0.32	0.08	-2	0.027856544
1204	107987290	LOC107987290	2.3	0.57	-2.0126	0.005957904
1205	105372267	NFILZ	1.01	0.25	-2.014355	1.85E-08
1206	4050	LTB	9.52	2.35	-2.018301	3.84E-10
1207	1102	RCBTB2	6.73	1.66	-2.019423	6.82E-30
1208	107987457	LOC107987457	2.12	0.52	-2.027481	4.85E-10
1209	105379547	LOC105379547	0.37	0.09	-2.039528	0.031563321
1210	55799	CACNA2D3	1.96	0.47	-2.060121	1.45E-12
1211	127534	GJB4	2.17	0.52	-2.061112	5.79E-06
1212	54039	PCBP3	1.17	0.28	-2.06301	8.47E-05
1213	50624	CUZD1	1.43	0.34	-2.072408	5.18E-05
1214	259307	IL4I1	5.46	1.29	-2.08153	2.85E-14
1215	10677	AVIL	1.19	0.28	-2.087463	3.87E-05
1216	10269	ZMPSTE24	29.9	7	-2.094719	1.58E-139
1217	202309	GAPT	13.88	3.22	-2.107875	3.48E-55

1218	79861	TUBAL3	14.6	3.38	-2.110873	8.08E-38
1219	144453	BEST3	0.39	0.09	-2.115477	0.010713404
1220	3604	TNFRSF9	6.86	1.58	-2.118284	5.26E-68
1221	6503	SLA	4.23	0.97	-2.124601	4.52E-20
1222	84163	GTF2IRD2	1.71	0.39	-2.13245	2.03E-04
1223	5343	PLGLB1	0.57	0.13	-2.13245	7.65E-04
1224	2687	GGT5	1.5	0.34	-2.141356	3.56E-06
1225	129881	CCDC173	10.78	2.44	-2.143404	2.92E-35
1226	221143	EEF1AKMT1	9.34	2.1	-2.153033	2.02E-13
1227	9350	CER1	3.85	0.86	-2.16245	1.73E-07
1228	359710	BPIFB3	0.59	0.13	-2.182203	0.019216508
1229	654429	LRTM2	2.59	0.57	-2.183918	3.58E-20
1230	947	CD34	1.38	0.3	-2.201634	1.22E-05
1231	412	STS	0.56	0.12	-2.222392	1.79E-07
1232	91752	ZNF804A	7.3	1.53	-2.254365	2.78E-59
1233	9173	IL1RL1	6.27	1.31	-2.258899	8.40E-35
1234	2041	EPHA1	13.23	2.75	-2.26631	7.78E-77
1235	57408	LRTM1	2.61	0.54	-2.273018	1.46E-06
1236	84915	FAM222A	4.61	0.95	-2.278767	1.27E-30
1237	89790	SIGLEC10	2.05	0.42	-2.287163	6.46E-09
1238	79785	RERGL	0.88	0.18	-2.289507	0.034297158
1239	91584	PLXNA4	4.11	0.84	-2.290677	6.80E-102
1240	84959	UBASH3B	54.45	11.08	-2.296974	0
1241	9308	CD83	10.14	2.06	-2.299341	7.75E-41
1242	84868	HAVCR2	0.79	0.16	-2.303781	8.06E-04
1243	7225	TRPC6	1.64	0.33	-2.313158	8.23E-14
1244	80763	SPX	16.53	3.31	-2.320184	1.91E-65
1245	647174	SERPINE3	0.05	0.01	-2.321928	0.01909782
1246	57053	CHRNA10	0.4	0.08	-2.321928	0.006852312
1247	122402	TDRD9	0.3	0.06	-2.321928	0.006514205
1248	4049	LTA	3.51	0.7	-2.326044	3.37E-06
1249	924	CD7	4.12	0.81	-2.346651	4.71E-09
1250	5452	POU2F2	4.35	0.84	-2.372554	8.42E-47
1251	93978	CLEC6A	0.52	0.1	-2.378512	0.034301498
1252	100049587	SIGLEC14	1.53	0.29	-2.399407	1.82E-08
1253	6547	SLC8A3	0.53	0.1	-2.405992	5.79E-06
1254	100996939	PYURF	53.05	9.81	-2.435028	8.29E-115
1255	9381	OTOF	0.38	0.07	-2.440573	1.67E-04
1256	105374013	LOC105374013	8.96	1.64	-2.449803	5.53E-70
1257	8605	PLA2G4C	0.22	0.04	-2.459432	0.034117672
1258	2251	FGF6	3.45	0.62	-2.476256	3.84E-14
1259	1795	DOCK3	0.34	0.06	-2.5025	1.38E-04
1260	441155	LOC441155	0.17	0.03	-2.5025	0.034100386
1261	353345	GPR141	1.55	0.27	-2.521237	2.01E-13
1262	130367	SGPP2	0.46	0.08	-2.523562	3.42E-05
1263	3224	HOXC8	0.69	0.12	-2.523562	4.75E-04
1264	56833	SLAMF8	8.65	1.5	-2.527738	5.06E-46
1265	5553	PRG2	109.75	18.94	-2.534713	3.69E-277
1266	51301	GCNT4	0.47	0.08	-2.554589	1.16E-05
1267	5342	PLGLB2	1	0.17	-2.556393	4.65E-07
1268	2123	EVI2A	0.3	0.05	-2.584963	0.020169544
1269	595	CCND1	25.73	4.21	-2.611559	1.49E-229
1270	151056	PLB1	0.49	0.08	-2.61471	3.48E-07
1271	60437	CDH26	2.83	0.46	-2.621096	3.83E-24
1272	284338	PRR19	1.25	0.2	-2.643856	4.75E-04
1273	80086	TUBA4B	1.08	0.17	-2.667425	0.001354073
1274	100288413	ERVMER34-1	0.32	0.05	-2.678072	0.003818497
1275	6310	ATXN1	0.52	0.08	-2.70044	2.59E-13
1276	973	CD79A	8.01	1.22	-2.714921	1.38E-15
1277	285	ANGPT2	0.66	0.1	-2.722466	3.19E-08
1278	643	CXCR5	0.14	0.02	-2.807355	0.034096068

1279	440730	TRIM67	0.77	0.11	-2.807355	1.83E-16
1280	50839	TAS2R10	1.34	0.19	-2.818162	0.006724941
1281	642636	RAD21L1	0.3	0.04	-2.906891	0.034113349
1282	102723737	CT45A8	0.98	0.13	-2.91427	0.01909013
1283	8807	IL18RAP	1.31	0.17	-2.94596	7.21E-09
1284	3976	LIF	0.54	0.07	-2.947533	9.27E-06
1285	163154	PRR22	0.85	0.11	-2.949959	0.003819081
1286	85508	SCRT2	6.78	0.86	-2.978877	7.32E-52
1287	343035	RD3	0.64	0.08	-3	1.61E-06
1288	84627	ZNF469	0.08	0.01	-3	0.004017445
1289	51314	NME8	0.72	0.09	-3	1.41E-04
1290	89822	KCNK17	2.46	0.3	-3.035624	7.22E-09
1291	26212	OR2B6	2.4	0.28	-3.099536	4.37E-05
1292	653489	RGPD3	1.72	0.2	-3.104337	4.18E-28
1293	2707	GJB3	0.35	0.04	-3.129283	0.034104706
1294	11167	FSTL1	0.09	0	-3.169925	0.0264876
1295	56122	PCDHB14	0.18	0.02	-3.169925	0.019087569
1296	388372	CCL4L1	1.1	0.12	-3.196397	0.019100384
1297	3067	HDC	45.12	4.88	-3.208814	4.06E-269
1298	6276	S100A5	0.65	0.07	-3.215013	0.034109027
1299	150221	RIMBP3C	0.1	0	-3.321928	0.013642202
1300	283446	MYO1H	0.1	0.01	-3.321928	0.034126322
1301	57569	ARHGAP20	0.1	0.01	-3.321928	0.034121996
1302	241	ALOX5AP	2.01	0.2	-3.329124	2.03E-04
1303	200350	FOXD4L1	0.41	0.04	-3.357552	0.005793672
1304	102724594	U2AF1L5	44.17	4.27	-3.370759	1.65E-86
1305	440712	RHEX	1.5	0.14	-3.421464	1.11E-04
1306	160762	CCDC63	0.43	0.04	-3.426265	0.019085008
1307	51725	FBXO40	0.11	0.01	-3.459432	0.019095256
1308	390439	OR11G2	0.96	0.08	-3.584963	0.005794534
1309	6330	SCN4B	0.12	0	-3.584963	0.01364409
1310	150094	SIK1	0.13	0	-3.70044	0.006969598
1311	273	AMPH	0.14	0	-3.807355	0.026491047
1312	389337	ARHGEF37	0.14	0.01	-3.807355	0.019092693
1313	116511	MAS1L	0.88	0.06	-3.874469	0.001696433
1314	7691	ZNF132	0.15	0	-3.906891	0.026466939
1315	9915	ARNT2	4.39	0.28	-3.970722	9.13E-92
1316	107985687	LOC107985687	0.16	0.01	-4	0.005795397
1317	378948	RBMY1B	0.51	0.03	-4.087463	0.003139505
1318	3238	HOXD12	0.18	0	-4.169925	0.02647038
1319	2814	GP5	0.37	0.02	-4.209453	7.12E-05
1320	26290	GALNT8	0.2	0	-4.321928	0.026460059
1321	100423062	IGLL5	3.78	0.18	-4.392317	3.86E-14
1322	10205	MPZL2	0.22	0	-4.459432	0.026477266
1323	3589	IL11	0.22	0	-4.459432	0.013640313
1324	10288	LILRB2	1.12	0.05	-4.485427	8.56E-11
1325	100529241	HSPE1-MOB4	0.23	0	-4.523562	4.61E-04
1326	57834	CYP4F11	0.25	0	-4.643856	0.001812195
1327	30820	KCNIP1	0.27	0	-4.754888	0.013638426
1328	100506164	HSFX1	0.28	0.01	-4.807355	0.013632766
1329	219995	MS4A15	0.29	0	-4.857981	0.026463499
1330	5724	PTAFR	3.61	0.12	-4.910893	7.67E-52
1331	80231	CXorf21	0.31	0	-4.954196	0.013636538
1332	51676	ASB2	0.32	0	-5	0.001812489
1333	100526664	LY75-CD302	0.33	0	-5.044394	3.71E-13
1334	221188	ADGRG5	2.97	0.09	-5.044394	2.77E-36
1335	914	CD2	0.36	0	-5.169925	0.013645979
1336	653659	TMEM183B	0.37	0	-5.209453	0.013634652
1337	346171	ZFP57	0.38	0	-5.247928	0.003552389
1338	107984139	LOC107984139	0.38	0	-5.247928	0.026456621
1339	27033	ZBTB32	0.38	0	-5.247928	0.013647869

1340	9079	LDB2	0.4	0	-5.321928	1.17E-04
1341	102723360	LOC102723360	0.41	0	-5.357552	4.21E-10
1342	140	ADORA3	0.5	0	-5.643856	9.15E-04
1343	100533467	BIVM-ERCC5	0.53	0	-5.72792	6.18E-12
1344	107080644	CNPY3-GNMT	0.62	0	-5.954196	0.02648071
1345	1475	CSTA	0.85	0	-6.409391	0.026484154
1346	6779	STATH	0.99	0	-6.629357	0.026473822
1347	5940	RBMY1A1	1.77	0	-7.467606	1.03E-10
