# Supplementary material for: FLI1 induces erythroleukemia through opposing effects on UBASH3A and UBASH3B expression
Source: BMC Cancer. 2024 Mar 9;24:326. doi: 10.1186/s12885-024-12075-2 (PMC10925000; doi:10.1186/s12885-024-12075-2)
Supplement: Supplementary file 3 — Supplementary Materials 3. [file 12885_2024_12075_MOESM3_ESM.pptx]

## Slide 1
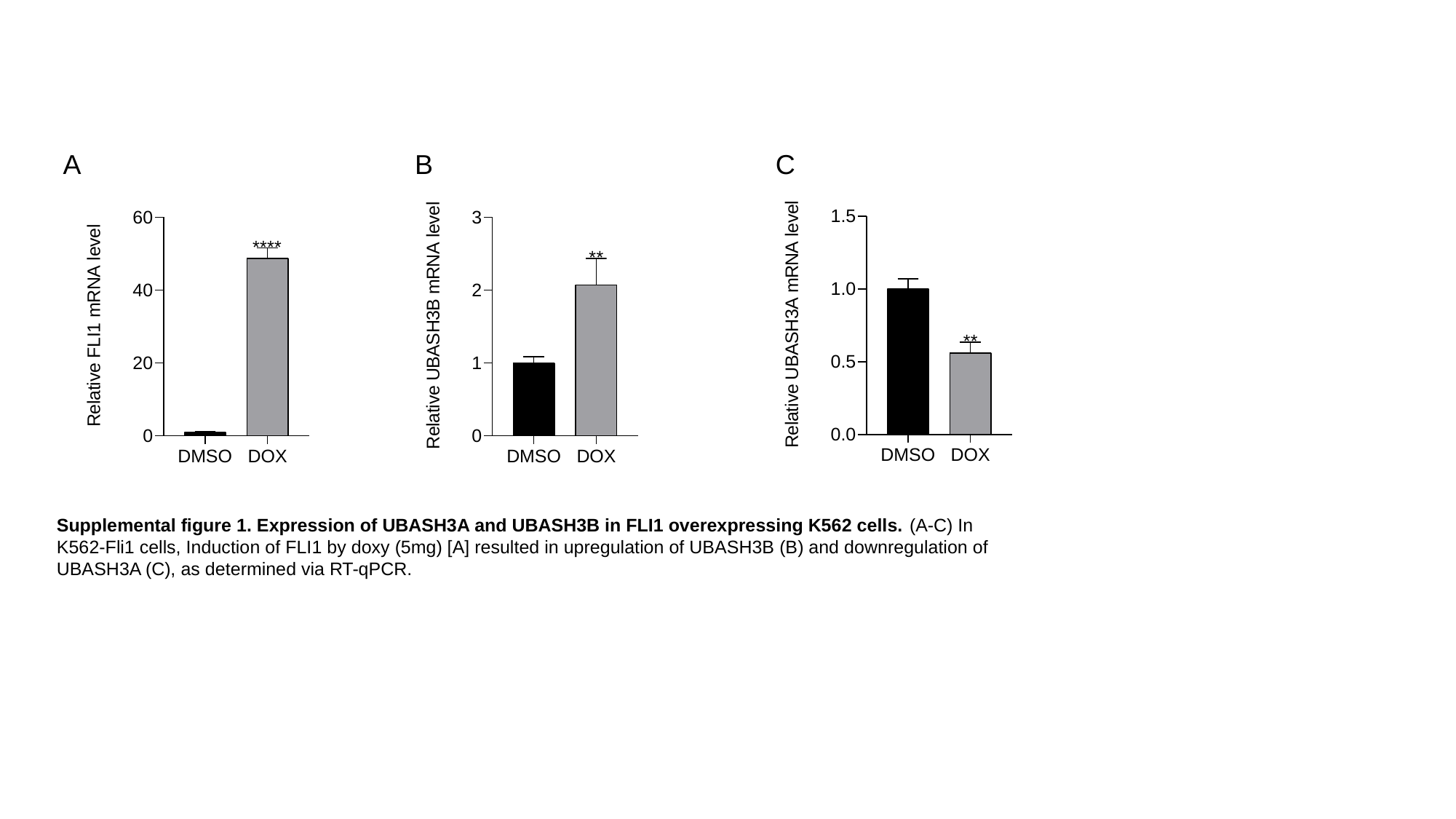

A B C
Supplemental figure 1. Expression of UBASH3A and UBASH3B in FLI1 overexpressing K562 cells. (A-C) In K562-Fli1 cells, Induction of FLI1 by doxy (5mg) [A] resulted in upregulation of UBASH3B (B) and downregulation of UBASH3A (C), as determined via RT-qPCR.

## Slide 2
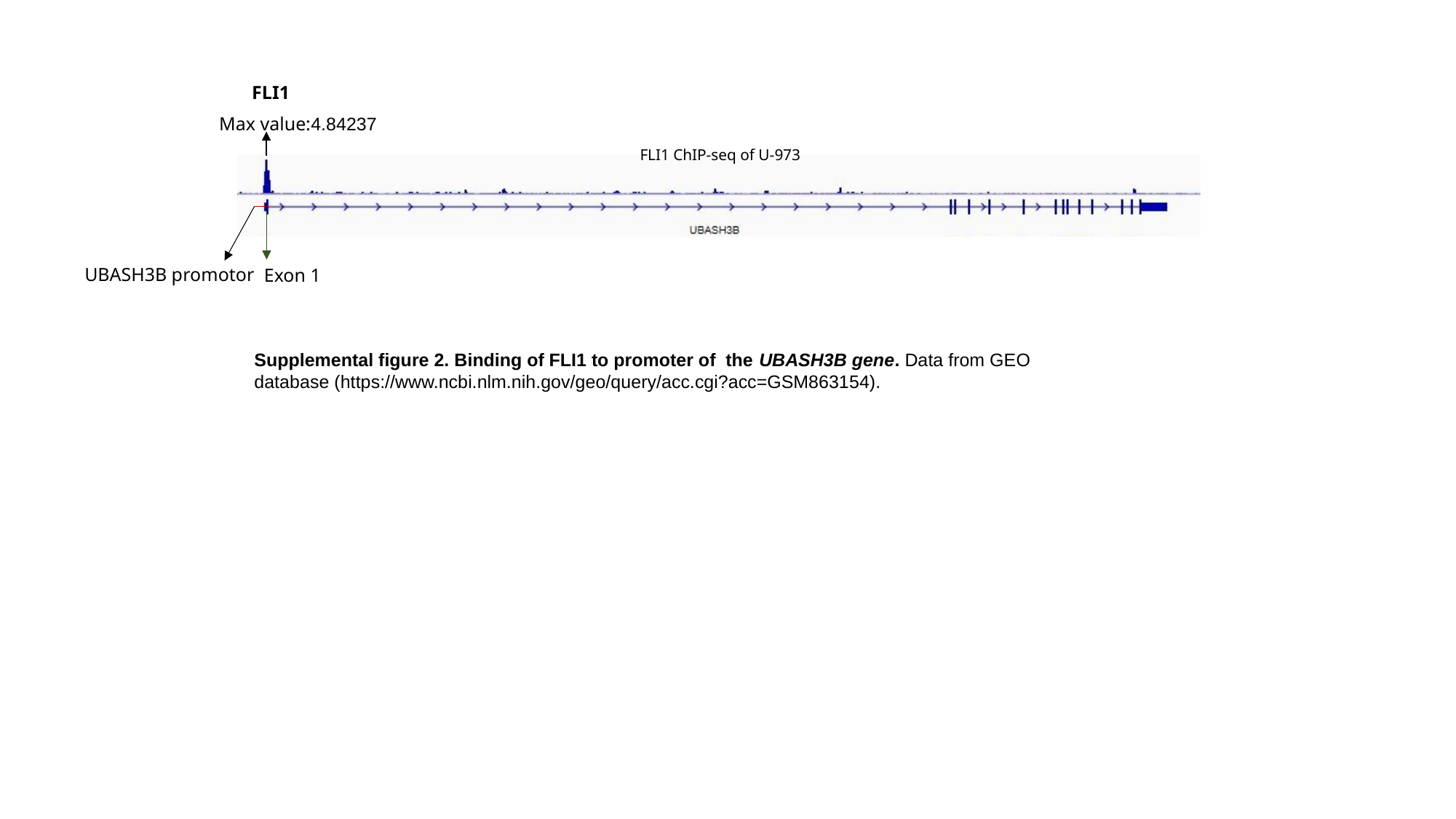

FLI1
Max value:4.84237
UBASH3B promotor
Exon 1
FLI1 ChIP-seq of U-973
Supplemental figure 2. Binding of FLI1 to promoter of the UBASH3B gene. Data from GEO database (https://www.ncbi.nlm.nih.gov/geo/query/acc.cgi?acc=GSM863154).

## Slide 3
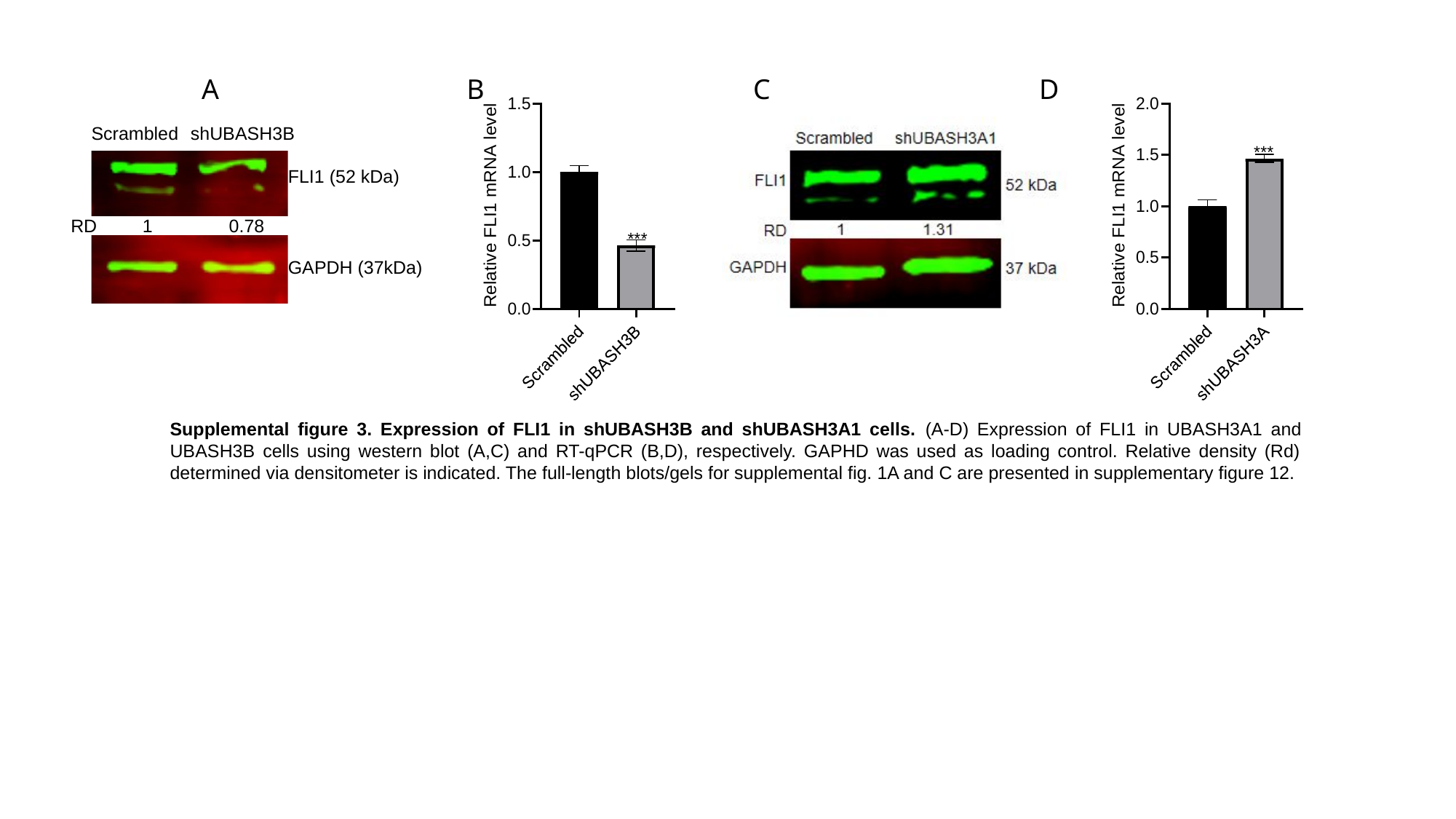

A B C D
Scrambled
shUBASH3B
FLI1 (52 kDa)
RD 1 0.78
GAPDH (37kDa)
Supplemental figure 3. Expression of FLI1 in shUBASH3B and shUBASH3A1 cells. (A-D) Expression of FLI1 in UBASH3A1 and UBASH3B cells using western blot (A,C) and RT-qPCR (B,D), respectively. GAPHD was used as loading control. Relative density (Rd) determined via densitometer is indicated. The full-length blots/gels for supplemental fig. 1A and C are presented in supplementary figure 12.

## Slide 4
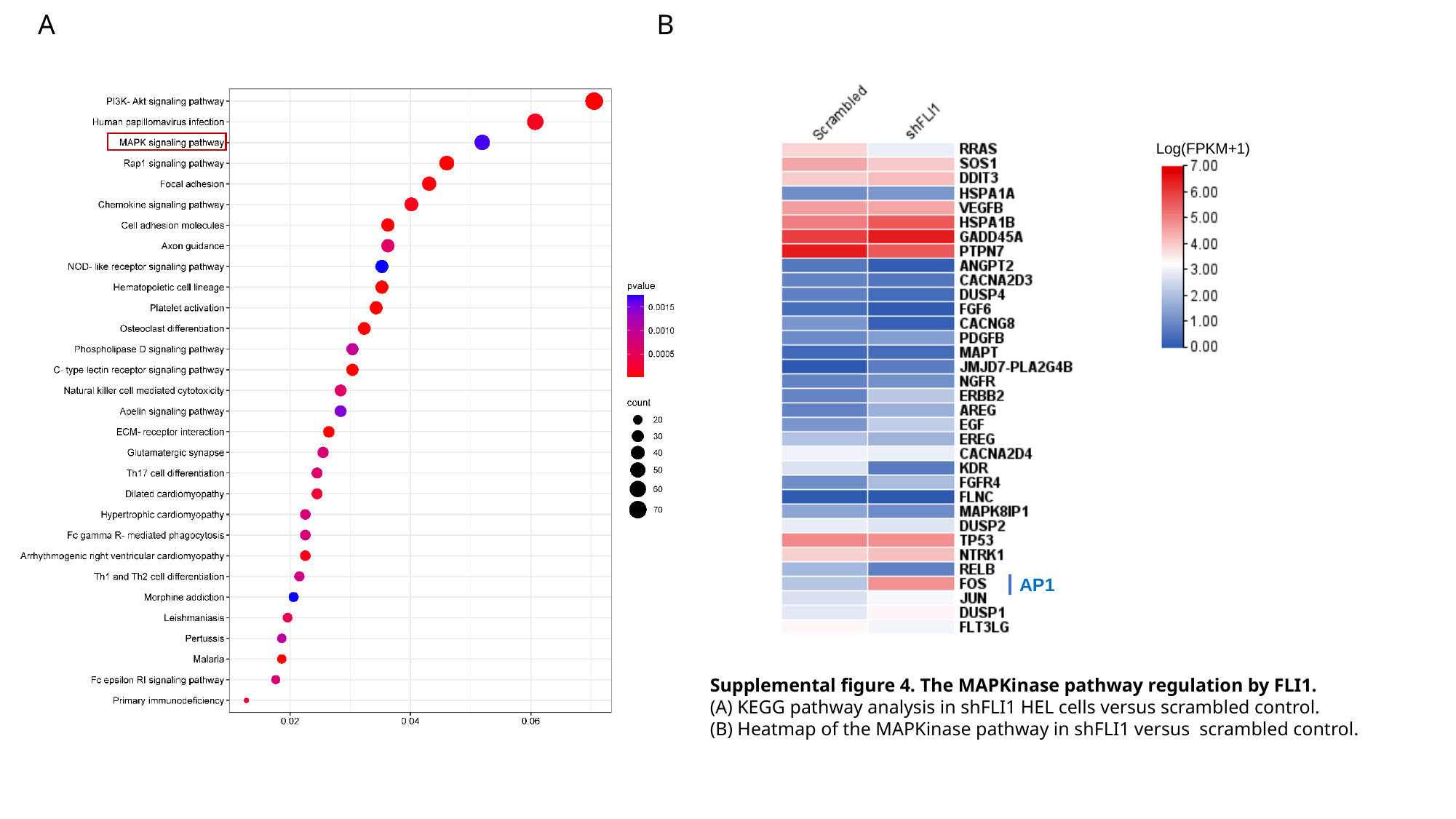

A B
Log(FPKM+1)
AP1
Supplemental figure 4. The MAPKinase pathway regulation by FLI1.
(A) KEGG pathway analysis in shFLI1 HEL cells versus scrambled control.
(B) Heatmap of the MAPKinase pathway in shFLI1 versus scrambled control.

## Slide 5
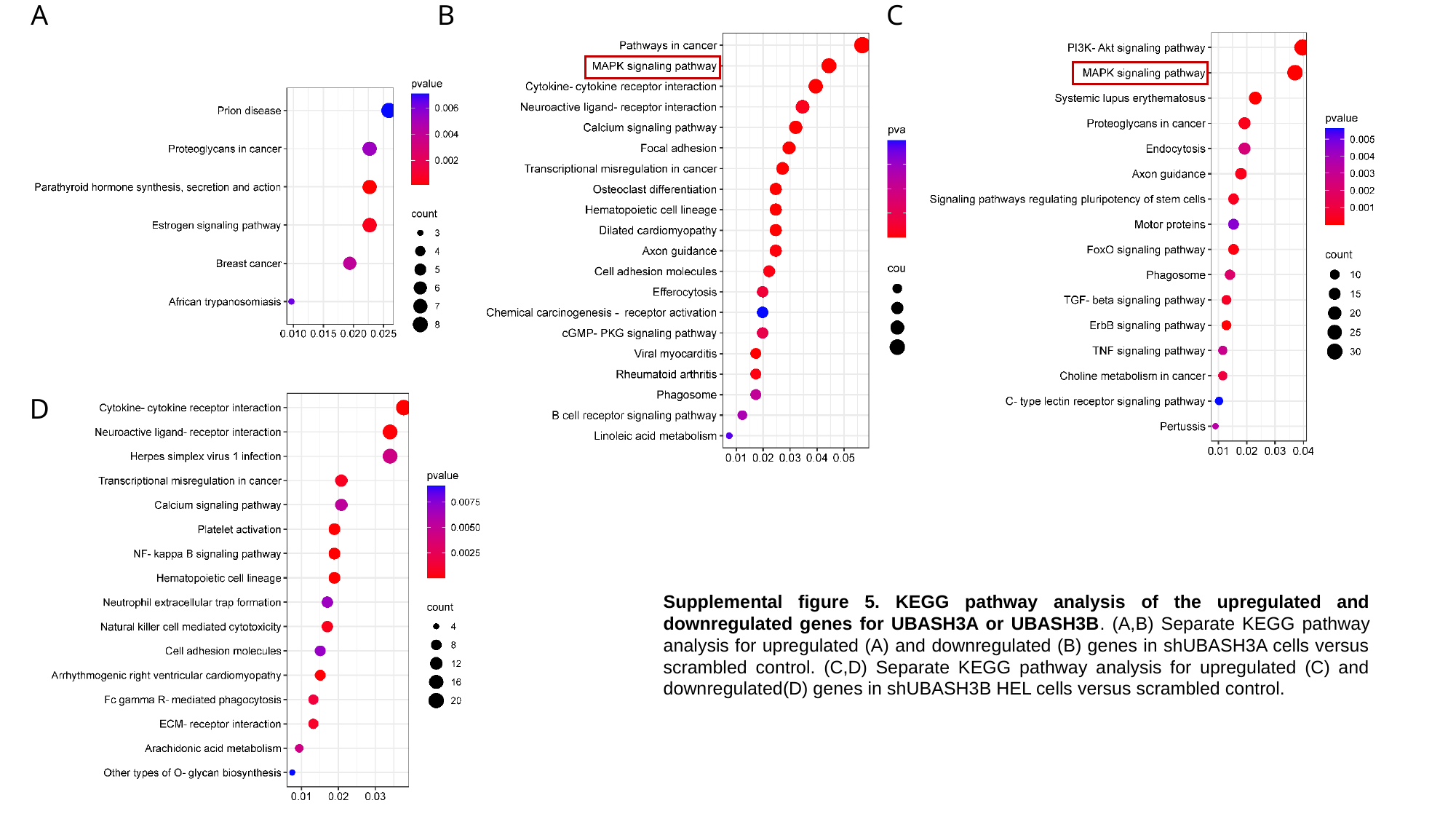

A B C
D
Supplemental figure 5. KEGG pathway analysis of the upregulated and downregulated genes for UBASH3A or UBASH3B. (A,B) Separate KEGG pathway analysis for upregulated (A) and downregulated (B) genes in shUBASH3A cells versus scrambled control. (C,D) Separate KEGG pathway analysis for upregulated (C) and downregulated(D) genes in shUBASH3B HEL cells versus scrambled control.

## Slide 6
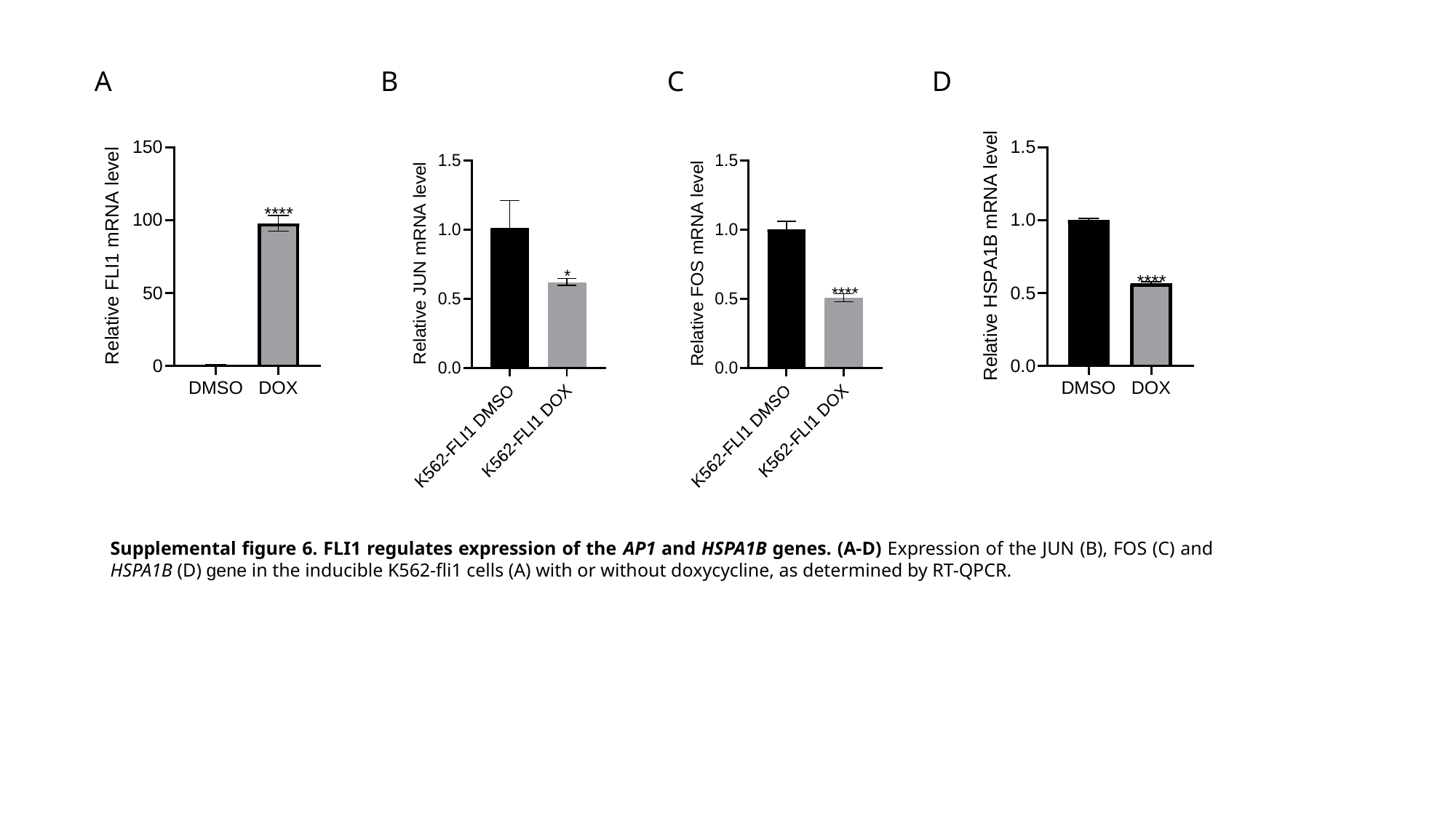

A B C D
Supplemental figure 6. FLI1 regulates expression of the AP1 and HSPA1B genes. (A-D) Expression of the JUN (B), FOS (C) and HSPA1B (D) gene in the inducible K562-fli1 cells (A) with or without doxycycline, as determined by RT-QPCR.

## Slide 7
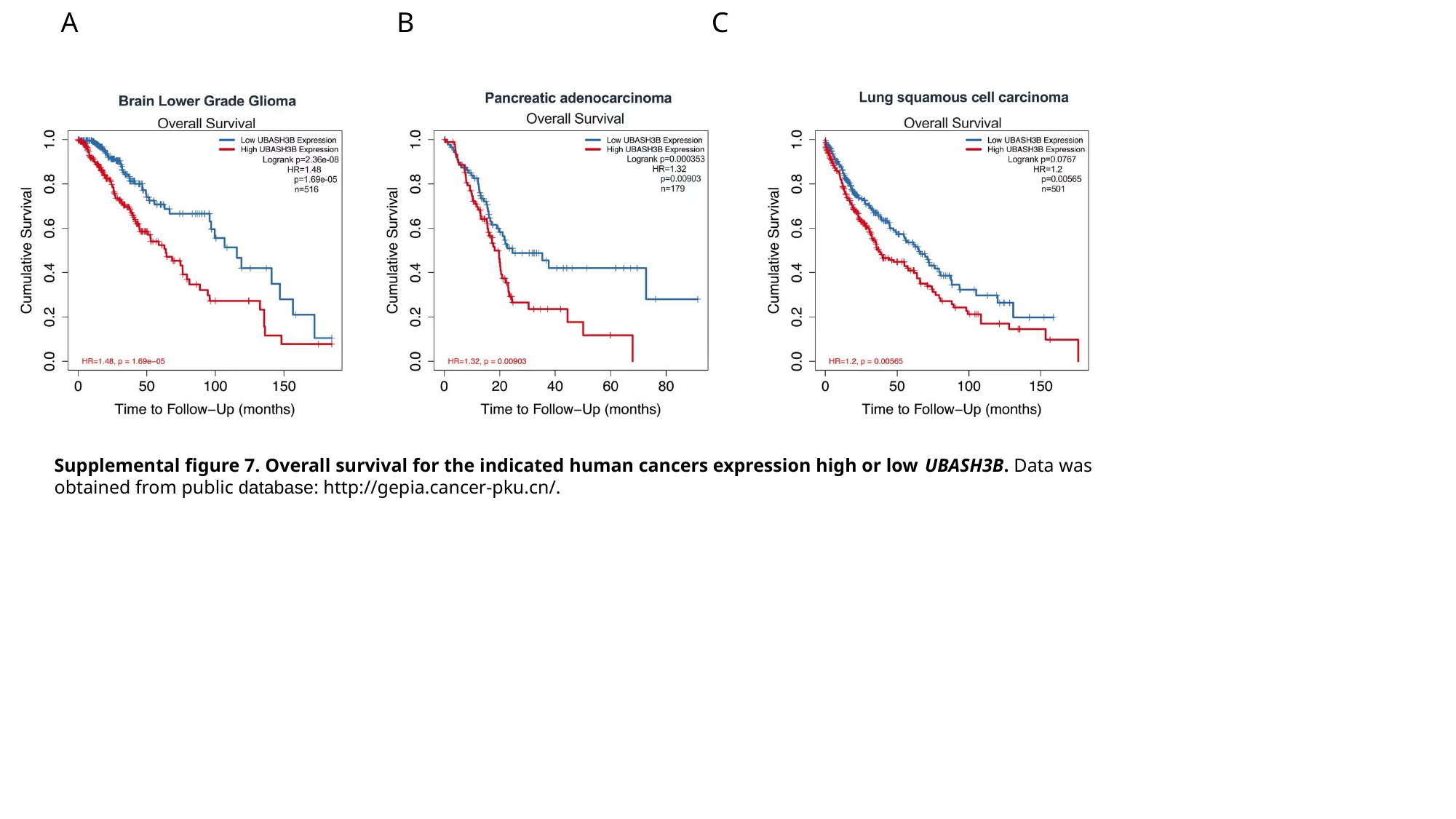

A B C
Supplemental figure 7. Overall survival for the indicated human cancers expression high or low UBASH3B. Data was obtained from public database: http://gepia.cancer-pku.cn/.

## Slide 8
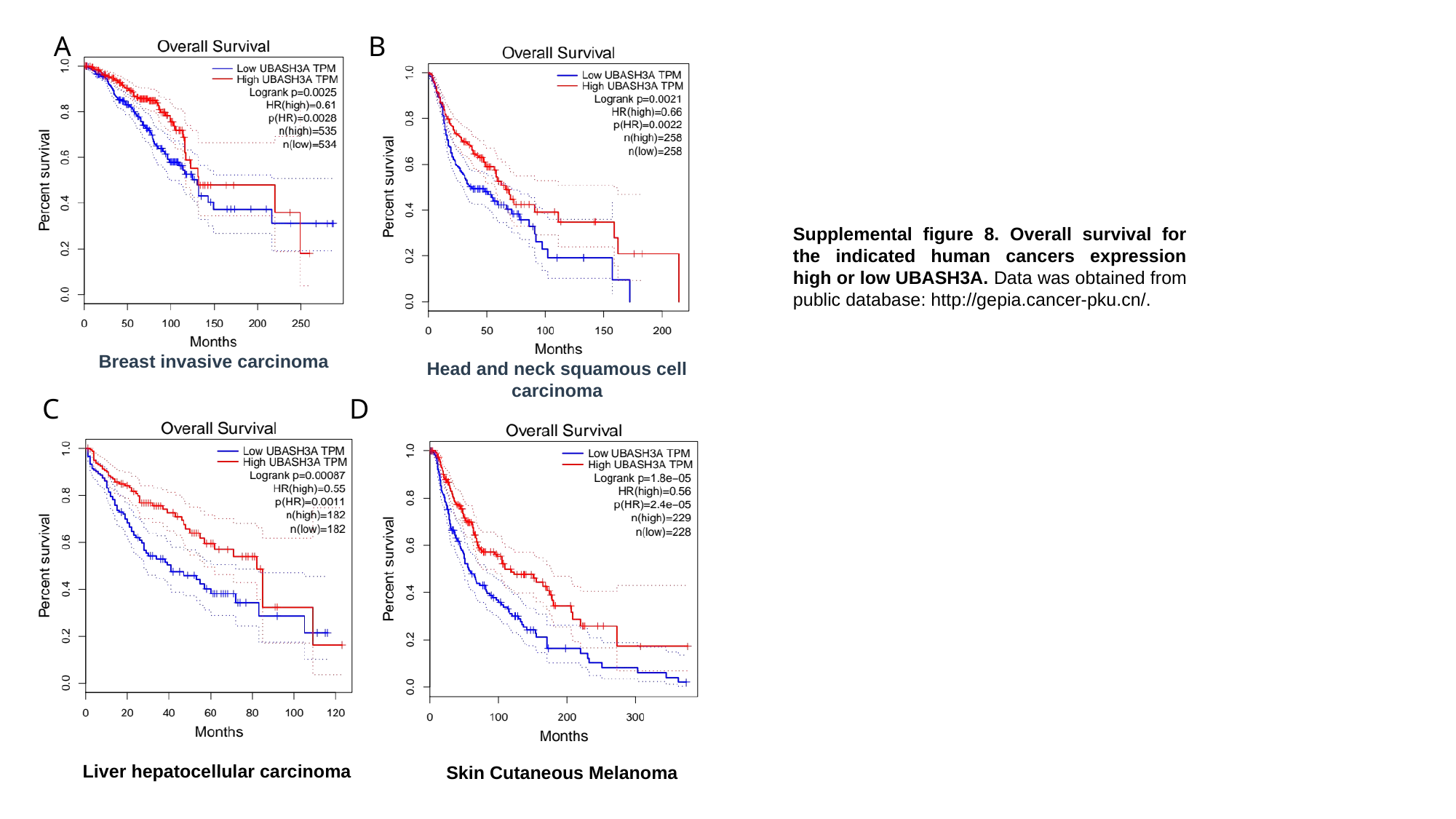

A B
Supplemental figure 8. Overall survival for the indicated human cancers expression high or low UBASH3A. Data was obtained from public database: http://gepia.cancer-pku.cn/.
Breast invasive carcinoma
Head and neck squamous cell carcinoma
C D
Liver hepatocellular carcinoma
Skin Cutaneous Melanoma

## Slide 9
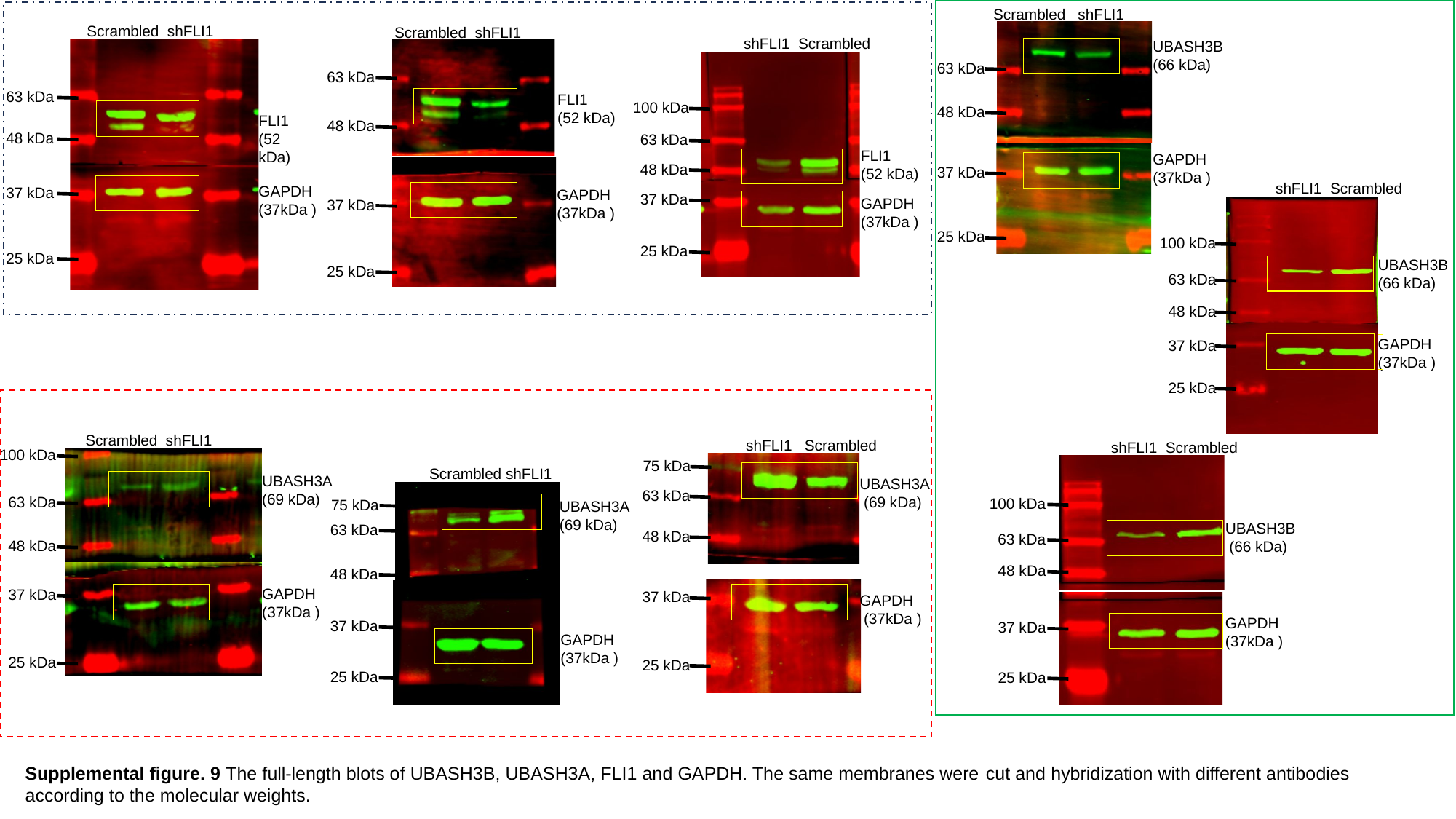

Scrambled shFLI1
Scrambled shFLI1
Scrambled shFLI1
shFLI1 Scrambled
UBASH3B
(66 kDa)
63 kDa
63 kDa
63 kDa
FLI1
(52 kDa)
100 kDa
48 kDa
FLI1
(52 kDa)
48 kDa
48 kDa
63 kDa
FLI1
(52 kDa)
GAPDH
(37kDa )
48 kDa
37 kDa
shFLI1 Scrambled
GAPDH
(37kDa )
37 kDa
GAPDH
(37kDa )
37 kDa
GAPDH
(37kDa )
37 kDa
25 kDa
100 kDa
25 kDa
25 kDa
UBASH3B
(66 kDa)
25 kDa
63 kDa
48 kDa
GAPDH
(37kDa )
37 kDa
25 kDa
Scrambled shFLI1
shFLI1 Scrambled
 shFLI1 Scrambled
100 kDa
75 kDa
Scrambled shFLI1
UBASH3A
(69 kDa)
UBASH3A
 (69 kDa)
63 kDa
63 kDa
100 kDa
75 kDa
UBASH3A
(69 kDa)
UBASH3B
 (66 kDa)
63 kDa
48 kDa
63 kDa
48 kDa
48 kDa
48 kDa
GAPDH
(37kDa )
37 kDa
37 kDa
GAPDH
 (37kDa )
GAPDH
(37kDa )
37 kDa
37 kDa
GAPDH
(37kDa )
25 kDa
25 kDa
25 kDa
25 kDa
Supplemental figure. 9 The full-length blots of UBASH3B, UBASH3A, FLI1 and GAPDH. The same membranes were cut and hybridization with different antibodies according to the molecular weights.

## Slide 10
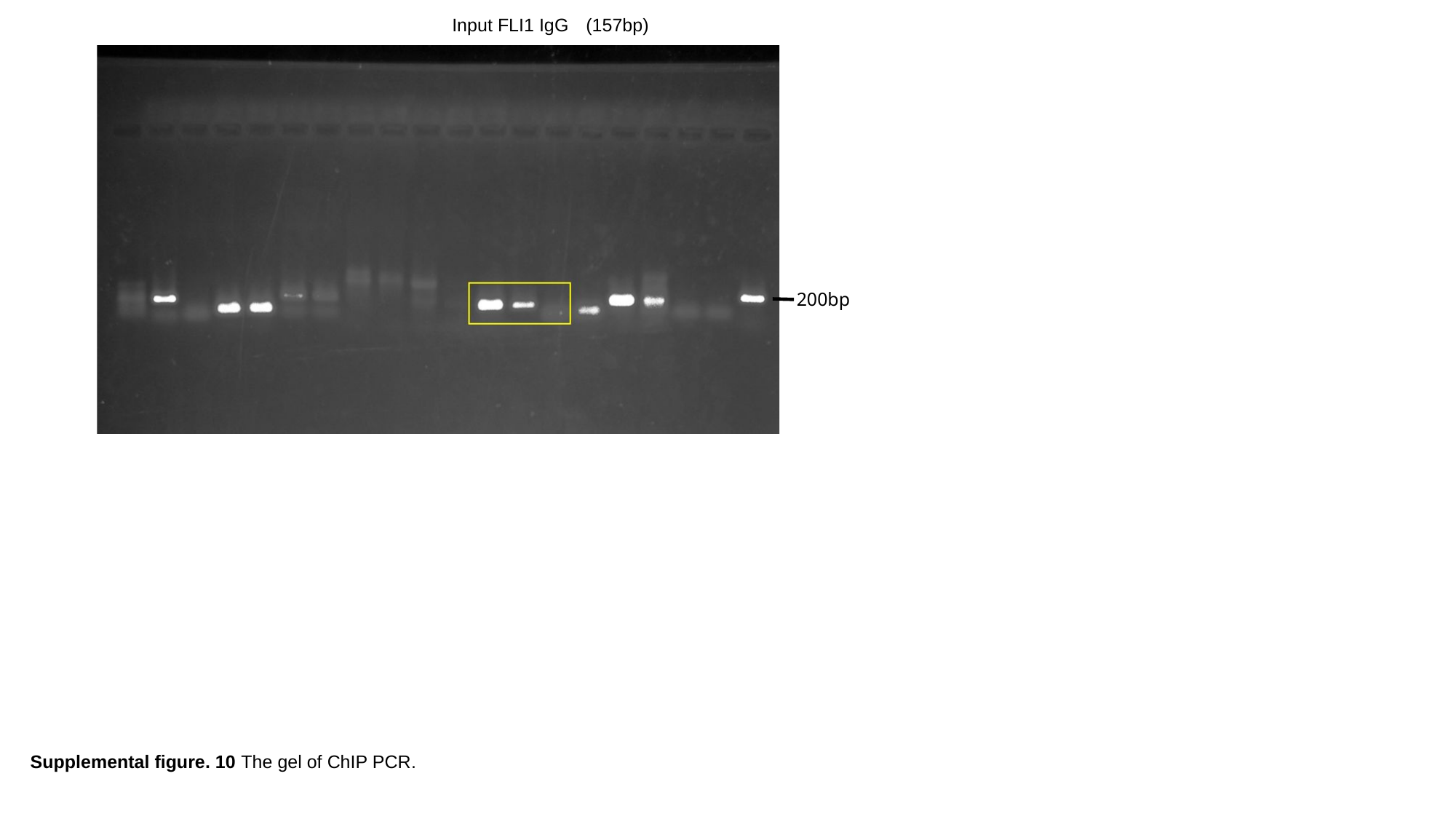

Input FLI1 IgG
(157bp)
200bp
Supplemental figure. 10 The gel of ChIP PCR.

## Slide 11
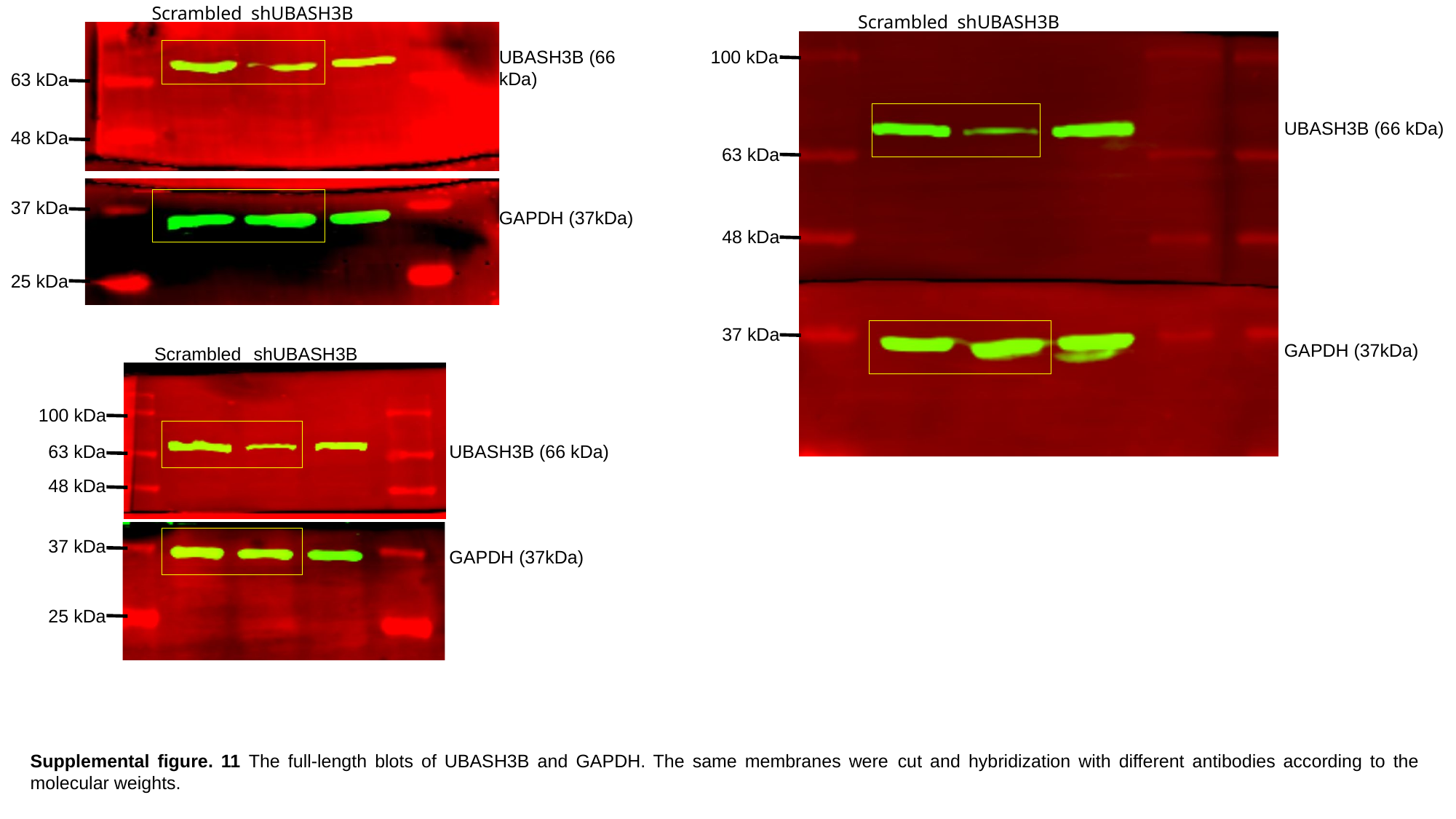

Scrambled
shUBASH3B
Scrambled
shUBASH3B
UBASH3B (66 kDa)
100 kDa
63 kDa
UBASH3B (66 kDa)
48 kDa
63 kDa
37 kDa
GAPDH (37kDa)
48 kDa
25 kDa
37 kDa
GAPDH (37kDa)
Scrambled
shUBASH3B
100 kDa
63 kDa
UBASH3B (66 kDa)
48 kDa
37 kDa
GAPDH (37kDa)
25 kDa
Supplemental figure. 11 The full-length blots of UBASH3B and GAPDH. The same membranes were cut and hybridization with different antibodies according to the molecular weights.

## Slide 12
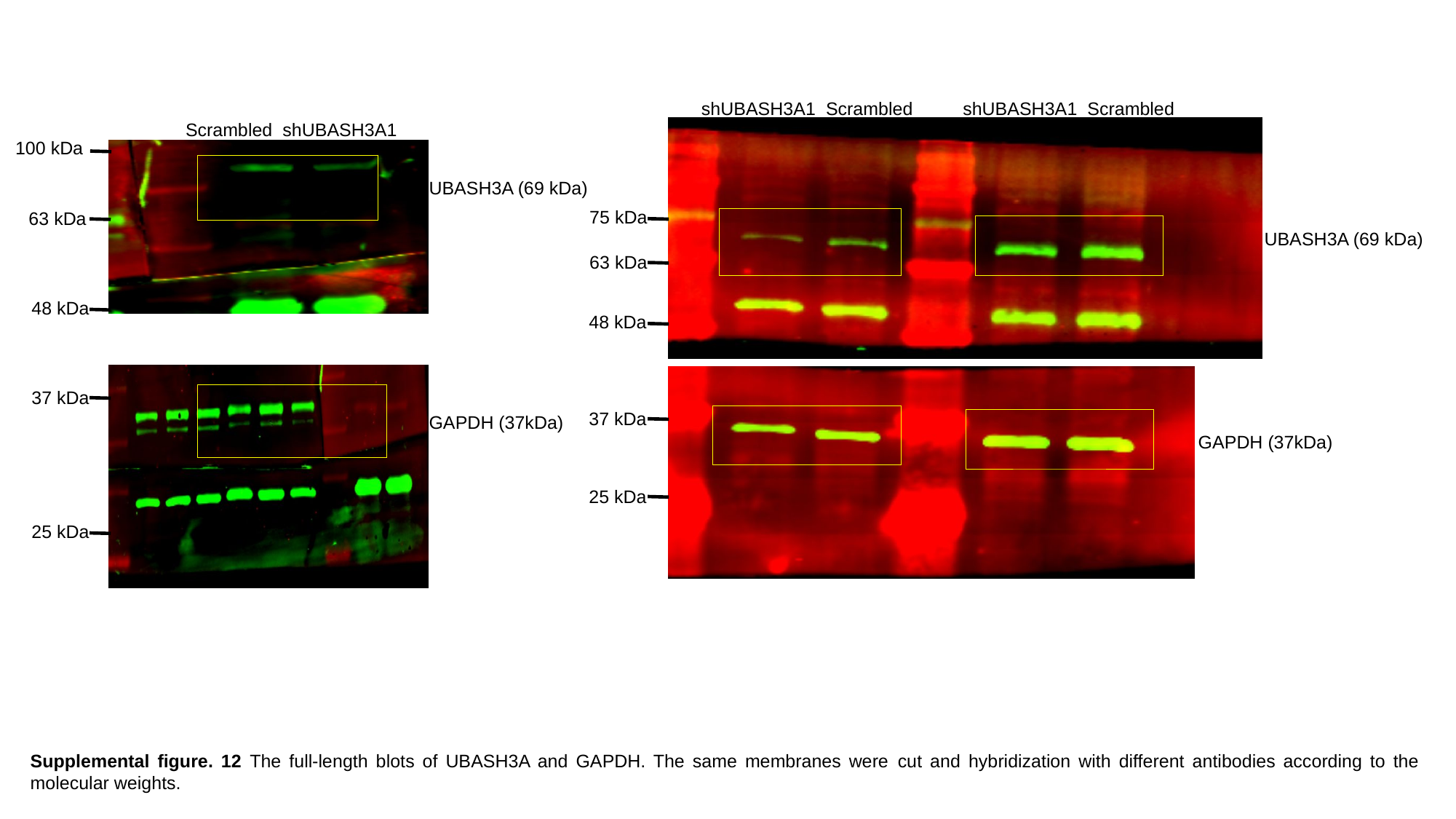

shUBASH3A1 Scrambled
shUBASH3A1 Scrambled
Scrambled shUBASH3A1
100 kDa
UBASH3A (69 kDa)
75 kDa
63 kDa
UBASH3A (69 kDa)
63 kDa
48 kDa
48 kDa
37 kDa
37 kDa
GAPDH (37kDa)
GAPDH (37kDa)
25 kDa
25 kDa
Supplemental figure. 12 The full-length blots of UBASH3A and GAPDH. The same membranes were cut and hybridization with different antibodies according to the molecular weights.

## Slide 13
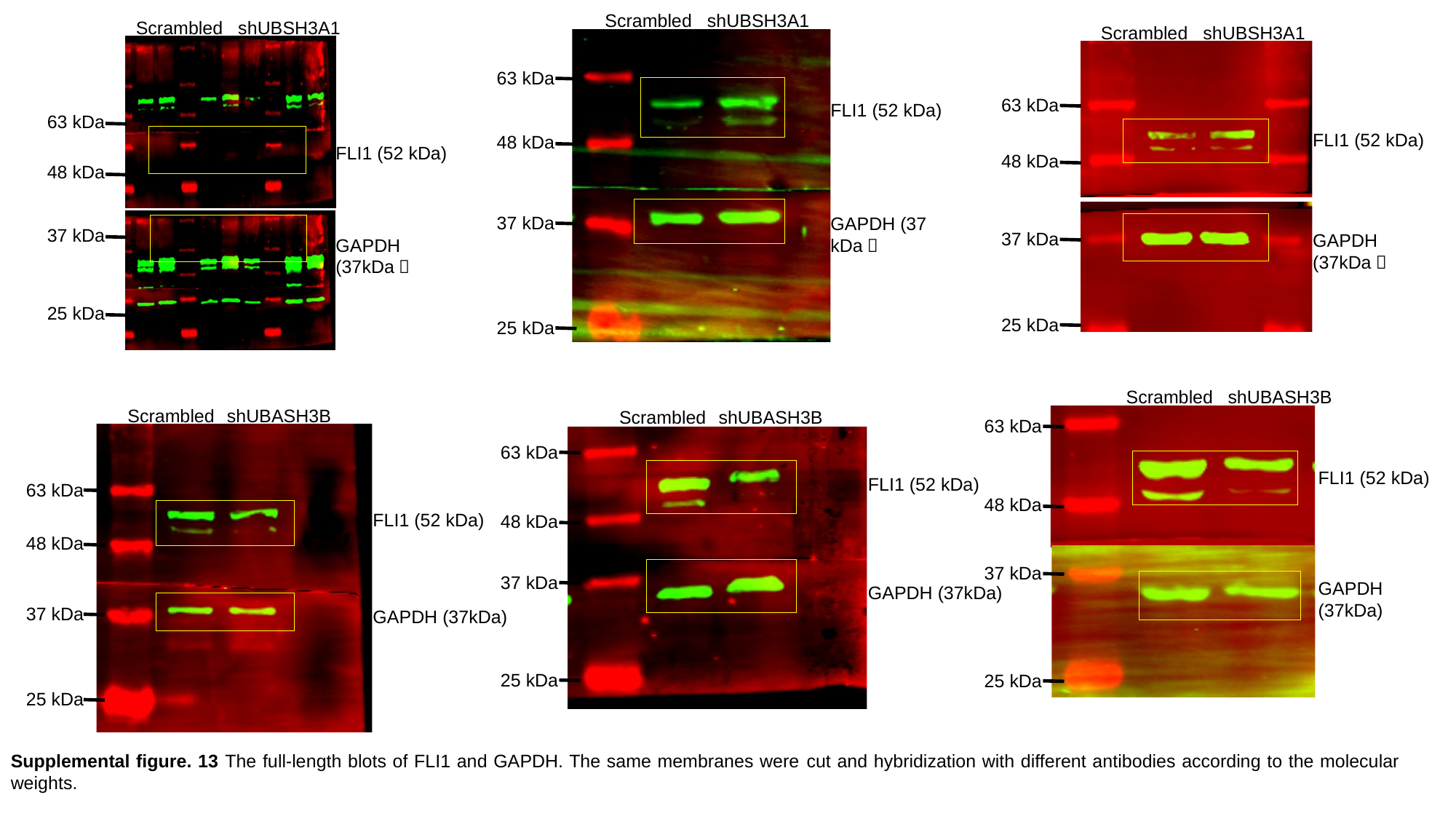

Scrambled shUBSH3A1
Scrambled shUBSH3A1
Scrambled shUBSH3A1
63 kDa
63 kDa
FLI1 (52 kDa)
63 kDa
FLI1 (52 kDa)
48 kDa
FLI1 (52 kDa)
48 kDa
48 kDa
37 kDa
GAPDH (37 kDa）
37 kDa
37 kDa
GAPDH (37kDa）
GAPDH (37kDa）
25 kDa
25 kDa
25 kDa
Scrambled
shUBASH3B
Scrambled
shUBASH3B
Scrambled
shUBASH3B
63 kDa
63 kDa
FLI1 (52 kDa)
FLI1 (52 kDa)
63 kDa
48 kDa
FLI1 (52 kDa)
48 kDa
48 kDa
37 kDa
37 kDa
GAPDH (37kDa)
GAPDH (37kDa)
37 kDa
GAPDH (37kDa)
25 kDa
25 kDa
25 kDa
Supplemental figure. 13 The full-length blots of FLI1 and GAPDH. The same membranes were cut and hybridization with different antibodies according to the molecular weights.
